# Publisher Correction: Pre-symptomatic Caspase-1 inhibitor delays cognitive decline in a mouse model of Alzheimer disease and aging

**DOI:** 10.1038/s41467-021-22789-7

**Published:** 2021-04-09

**Authors:** Joseph Flores, Anastasia Noël, Bénédicte Foveau, Olivier Beauchet, Andréa C. LeBlanc

**Affiliations:** 1 Bloomfield Center for Research in Aging, Lady Davis Institute for Medical Research, Jewish General Hospital, Montreal, Quebec Canada; 2 Department of Neurology and Neurosurgery, McGill University, Montreal, Quebec Canada; 3 Department of Medicine, Division of Geriatric Medicine, Sir Mortimer B. Davis - Jewish General Hospital, Montreal, Quebec Canada; 4 Lee Kong Chian School of Medicine, Nanyang Technological University, Singapore, Singapore; 5 Department of Anatomy and Cell Biology, McGill University, Montreal, Quebec Canada

Correction to: *Nature Communications* 10.1038/s41467-020-18405-9, published online 11 September 2020.

The original version of this Article contained an error in Fig. 5j, in which the *y* axis of the graphs on the top part of the panel was incorrectly labelled “2^−ΔCT^ *Casp6* (x10^−3^)” instead of “2^−ΔCT^ *Casp1* (x10^−3^)” This has been corrected in both the PDF and HTML versions of the Article.

