# Supplementary material for: Pre-symptomatic Caspase-1 inhibitor delays cognitive decline in a mouse model of Alzheimer disease and aging
Source: Nat Commun. 2020 Sep 11;11:4571. doi: 10.1038/s41467-020-18405-9 (PMC7486940; doi:10.1038/s41467-020-18405-9)
Supplement: Supplementary file 1 — Supplementary Information [file 41467_2020_18405_MOESM1_ESM.pdf]

Supplementary Information

for

Pre-symptomatic Caspase-1 inhibitor delays cognitive decline in a mouse model of Alzheimer disease and aging

Joseph Flores , Anastasia Noël , Bénédicte Foveau , Olivier Beauchet , Andréa C. LeBlanc*

*** Corresponding author:** Andrea LeBlanc, Ph.D., Bloomfield Center for Research in Aging, Lady Davis Institute for Medical Research, Sir Mortimer B Davis Jewish General Hospital, 3755 Ch. Côte-Ste-Catherine, Montréal, QC, Canada H3T 1E2. Tel.: +1 (514) 340 8222 ext. 4976. Fax: +1 (514) 340 8295. E-mail address: andrea.leblanc@mcgill.ca

Supplementary Figure 1. Flores et al., Delayed cognitive impairment in mouse model

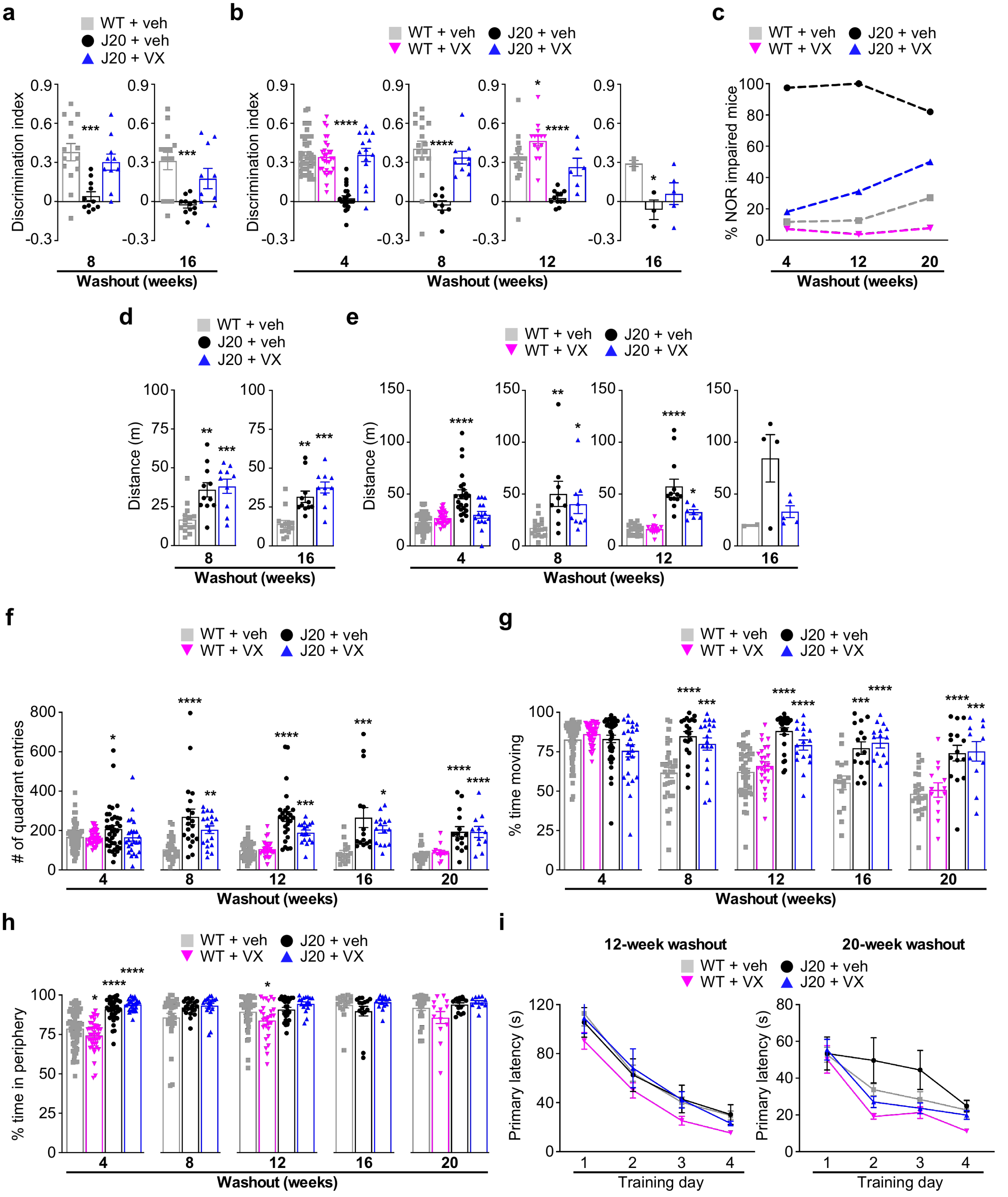

Supplementary Figure 1. Pre-symptomatic VX-765 treatment delays the onset of cognitive deficits in J20 mice. (a) NOR discrimination index of repeatedly tested vehicle-treated WT mice (■, n = 14), and

vehicle- (●, n = 11) and VX-765- (▲, n = 10) treated J20 mice at 8- and 16-week WO [8-week WO $F(2,32) = 9.192$, $p=0.0007$; 16-week WO $F(2,32) = 8.910$, $p=0.0013$, ANOVA, Dunnett's post-hoc compared to WT + vehicle]. (b) NOR discrimination index of remaining vehicle- and VX-765- (▼) treated WT, and vehicle- and VX-765-treated J20 mice utilized for post-mortem analyses at 4-week (n = 41 WT + veh, 29 WT + VX, 23 J20 + veh, 14 J20 + VX mice), 8-week (n = 17 WT + veh, 8 J20 + veh, 9 J20 + VX mice), 12-week (n = 21 WT + veh, 14 WT + VX, 12 J20 + veh, 6 J20 + VX mice), and 16-week (n = 3 WT + veh, 3 J20 + veh, 5 J20 + VX mice) WO [4-week WO $F(3,103) = 32.59$, $p=7x10^{-15}$; 8-week WO $F(2,31) = 13.38$, $p=6.47x10^{-5}$; 12-week WO $F(3,49) = 21.41$, $p=5.32x10^{-9}$; 16-week WO $F(2,8) = 4.431$, $p=0.0507$, ANOVA, Dunnett's post-hoc compared to WT + vehicle]. (c) Percentage of NOR impaired mice in each treatment group at 4-, 12-, and 20-week WO. (d) Open field distance traveled of repeatedly tested vehicle-treated WT mice (n = 14), and vehicle- (n = 11) and VX-765- (n = 10) treated J20 mice at 8- and 16-week WO [8-week WO $F(2,32) = 10.12$, $p=0.0004$; 16-week WO $F(2,32) = 16.35$, $p=1.28x10^{-5}$; ANOVA, Dunnett's post-hoc compared to WT + vehicle]. (e) Open field distance traveled of remaining mice utilized for post-mortem analyses at 4-week (n = 43 WT + veh, 29 WT + VX, 24 J20 + veh, 15 J20 + VX mice), 8-week (n = 17 WT + veh, 9 J20 + veh, 9 J20 + VX mice), 12-week (n = 20 WT + veh, 14 WT + VX, 13 J20 + veh, 7 J20 + VX mice), and 16-week (n = 2 WT + veh, 4 J20 + veh, 5 J20 + VX mice) WO [4-week WO $F(3,107) = 24.64$, $p=3.36x10^{-12}$; 8-week WO $F(2,32) = 6.587$, $p=0.0040$; 12-week WO $F(3,50) = 32.26$, $p=9.55x10^{-12}$; 16-week WO $F(2,8) = 4.619$, $p=0.0464$, ANOVA, Dunnett's post-hoc compared to WT + vehicle]. (f-h) Secondary open field measures of all mice at 4-week (n = 67 WT + veh, 42 WT + VX, 38 J20 + veh, 24 J20 + VX mice), 8-week (n = 31 WT + veh, 20 J20 + veh, 19 J20 + VX mice), 12-week (n = 44 WT + veh, 27 WT + VX, 27 J20 + veh, 17 J20 + VX mice), 16-week (n = 16 WT + veh, 15 J20 + veh, 15 J20 + VX mice), and 20-week (n = 24 WT + veh, 13 WT + VX, 15 J20 + veh, 11 J20 + VX mice) WO. (f) number of quadrant entries [4-week WO $F(3,167) = 2.971$, $p=0.0334$; 8-week WO $F(2,67) = 14.09$, $p=7.81x10^{-6}$; 12-week WO $F(3,111) = 35.43$, $p=1.0x10^{-15}$; 16-week WO $F(2,43) = 8.489$, $p=0.0008$; 20-week WO $F(3,59) = 13.90$, $p=5.78x10^{-7}$, ANOVA, Dunnett's post-hoc compared to

WT + vehicle]. (g) % time moving [4-week WO $F(3,167) = 3.526$, $p=0.0163$; 8-week WO $F(2,67) = 13.63$, $p=1.08x10^{-5}$; 12-week WO $F(3,111) = 23.14$, $p=1.04x10^{-11}$; 16-week WO $F(2,43) = 13.08$, $p=3.66x10^{-5}$; 20-week WO $F(3,59) = 11.66$, $p=4.23x10^{-6}$, ANOVA, Dunnett's post-hoc compared to WT + vehicle]. (h) thigmotaxis by % time spent in periphery [4-week WO $F(3,167) = 37.55$, $p=1.0x10^{-15}$; 8-week WO $F(2,67) = 3.721$, $p=0.0293$; 12-week WO $F(3,111) = 5.019$, $p=0.0027$; 20-week WO $F(3,59) = 3.013$, $p=0.0370$, ANOVA, Dunnett's post-hoc compared to WT + vehicle. (i) Barnes maze primary latency during learning acquisition phase of vehicle-treated WT (n = 22), VX-765-treated WT (n = 13), vehicle-treated J20 (n = 15), and VX-765-treated J20 (n = 10) mice at 12-week WO [treatment $F(3,224) = 3.093$, $p=0.0278$; training day $F(3,224) = 62.00$, $p=1.0x10^{-15}$, 2-way ANOVA, Dunnett's post-hoc compared to WT + vehicle], and vehicle-treated WT (n = 20), VX-765-treated WT (n = 13), vehicle-treated J20 (n = 11), and VX-765-treated J20 (n = 7) re-tested at 20-week WO [treatment $F(3,188) = 6.045$, $p=0.0006$; training day $F(3,188) = 20.81$, $p=1.10x10^{-11}$, 2-way ANOVA, Dunnett's post-hoc compared to WT + vehicle]. Data represents mean and s.e.m. *$p<0.05$, **$p<0.01$, ***$p<0.001$, ****$p<0.0001$ for all figure panels. Source data are provided as a Source Data file.

Supplementary Figure 2. Flores et al., Delayed cognitive impairment in mouse model

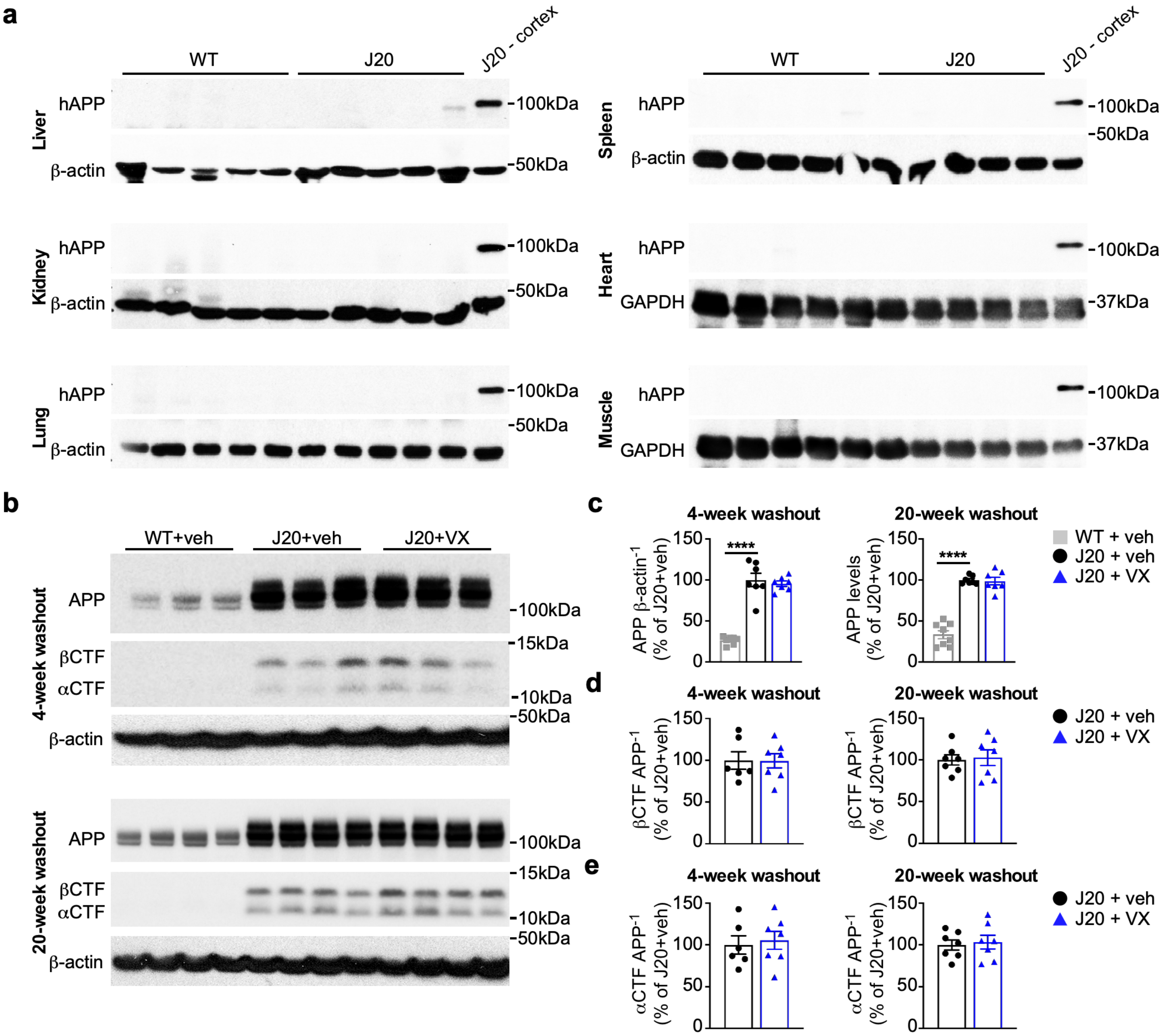

Supplementary Figure 2. APP processing is not altered after pre-symptomatic VX-765 treatment of J20 mice. (a) Human APP western blots in liver, kidney, lung, spleen, heart, and muscle peripheral tissues from 5 mice per group. (b) APP, βCTF, and αCTF western blots in hippocampus from 3 mice per group at 4- and 20-week WO using the anti-APP C-terminal antibody which detects both human and mouse protein. (c) Western blot quantification of APP at 4-week (n = 7 mice per group) and 20-week (n = 8 WT + veh, 7 J20 + veh, and 7 J20 + VX mice) WO in the hippocampus [4-week WO $F(2,18) = 64.07$, $p=6.52x10^{-9}$; 20-week WO $F(2,19) = 87.27$, $p=2.65x10^{-10}$, ANOVA, Dunnett's post-hoc compared to J20

+ vehicle]. (d-e) Western blot quantification of (d) βCTF and (e) αCTF in the hippocampus at 4-week (n = 6 J20 + veh and 7 J20 + VX mice) and 20-week (n = 7 mice per group) WO. Data represents mean and s.e.m. ****$p<0.0001$ for all figure panels. Source data are provided as a Source Data file.

Supplementary Figure 3. Flores et al., Delayed cognitive impairment in mouse model

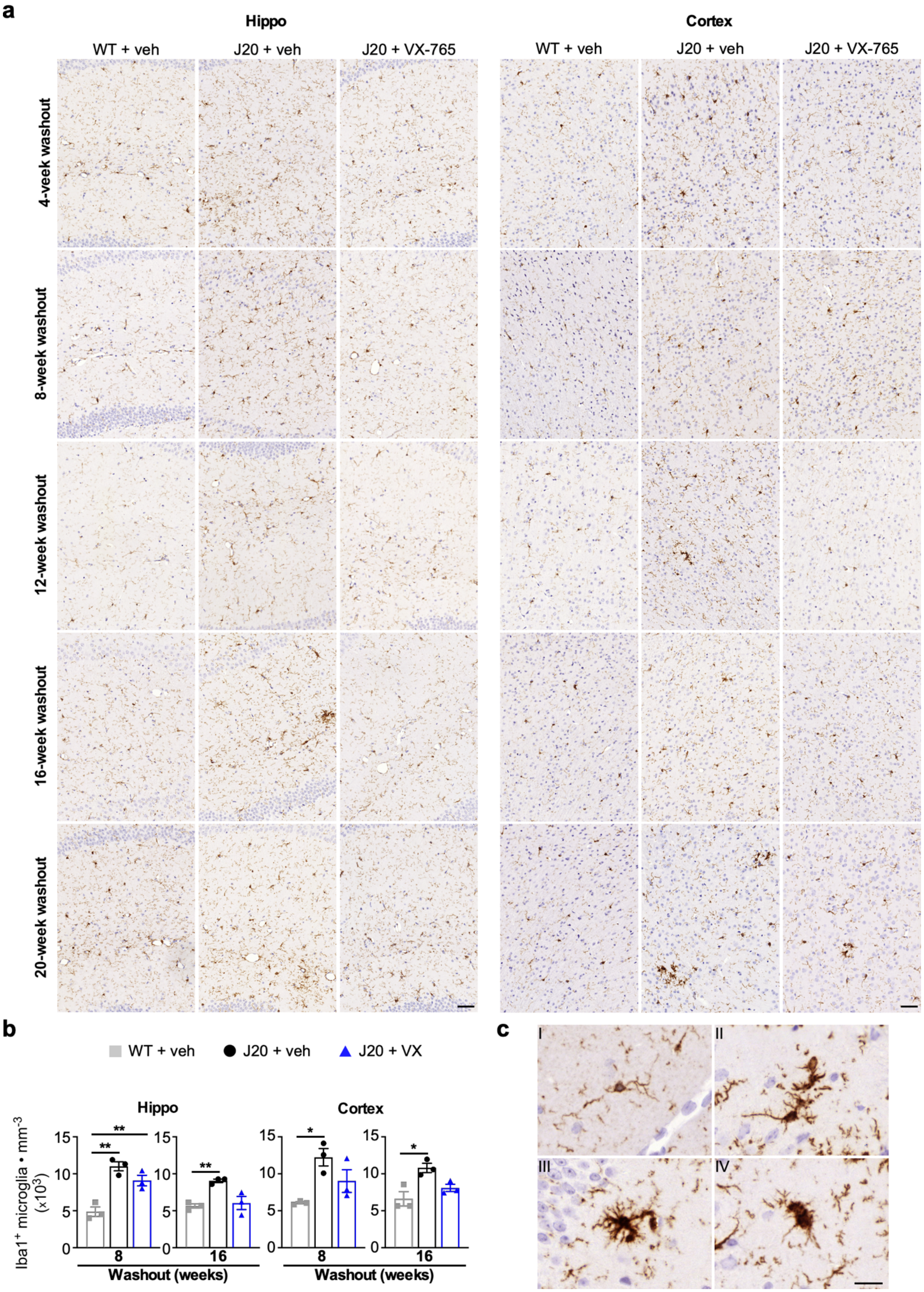

Supplementary Figure 3. Reduced hippocampal microglial Iba1 immunostaining in J20 mice after pre-symptomatic VX-765 treatment. (a) Representative micrographs of Iba1 positive microglia in hippocampal SLM (left panels) and cortical S1 region (right panels) from 3 mice per group. (b) Iba1 positive microglial quantification (n = 3 mice per group) from the pyramidal cell layer to the SLM in the hippocampal CA1 and cortical retrosplenial and S1 at 8- and 16-week WO [8-week WO hippo $F(2,6) = 22.58$, $p=0.0016$; 16-week WO hippo $F(2,6) = 11.33$, $p=0.0092$; 8-week WO cortex $F(2,6) = 7.549$, $p=0.0230$; 16-week WO cortex $F(2,6) = 8.631$, $p=0.0172$, ANOVA, Dunnett's post-hoc compare to WT + vehicle]. (c) Iba1 positive micrographs representing type I, II, III, and IV morphological subtypes of microglia. Scale bar in (a) = 50 μm, (c) = 20 μm. Data presented in (b) represents mean and s.e.m. *$p<0.05$, **$p<0.01$ for all figure panels. Source data are provided as a Source Data file.

Supplementary Figure 4. Flores et al., Delayed cognitive impairment in mouse model

a

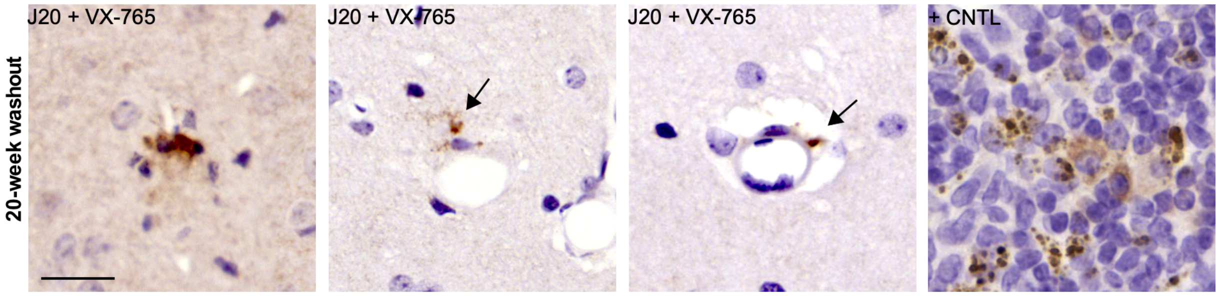

b

Animal ID	Treatment	CD68+ Hippo	CD68+ Cortex
160-3	J20+Veh	-	-
160-2	J20+Veh	-	-
151-2	J20+VX-765	+*	-
149-1	J20+VX-765	-	+
017-3	J20+VX-765	+*	-
017-6	J20+VX-765	+*	-
014-3	J20+Veh	-	-
016-6	J20+Veh	-	-
016-3	J20+VX-765	-	-
014-4	J20+Veh	-	-

*CD68 positive perivascular macrophage staining only

Supplementary Figure 4. No increase in CD68 positive staining in J20 mice after pre-symptomatic VX-765 treatment. (a) Representative micrographs from mice tested in (b) for CD68 positive staining resembling type IV microglia (left inset) and perivascular macrophages (black arrows, middle two insets) in VX-765-treated J20 mice at 20-week WO (CNTL right inset: spleen positive control). Scale bar = 20 μm. (b) Table showing mice positive for CD68 staining at 20-week WO.

Supplementary Figure 5. Flores et al., Delayed cognitive impairment in mouse model

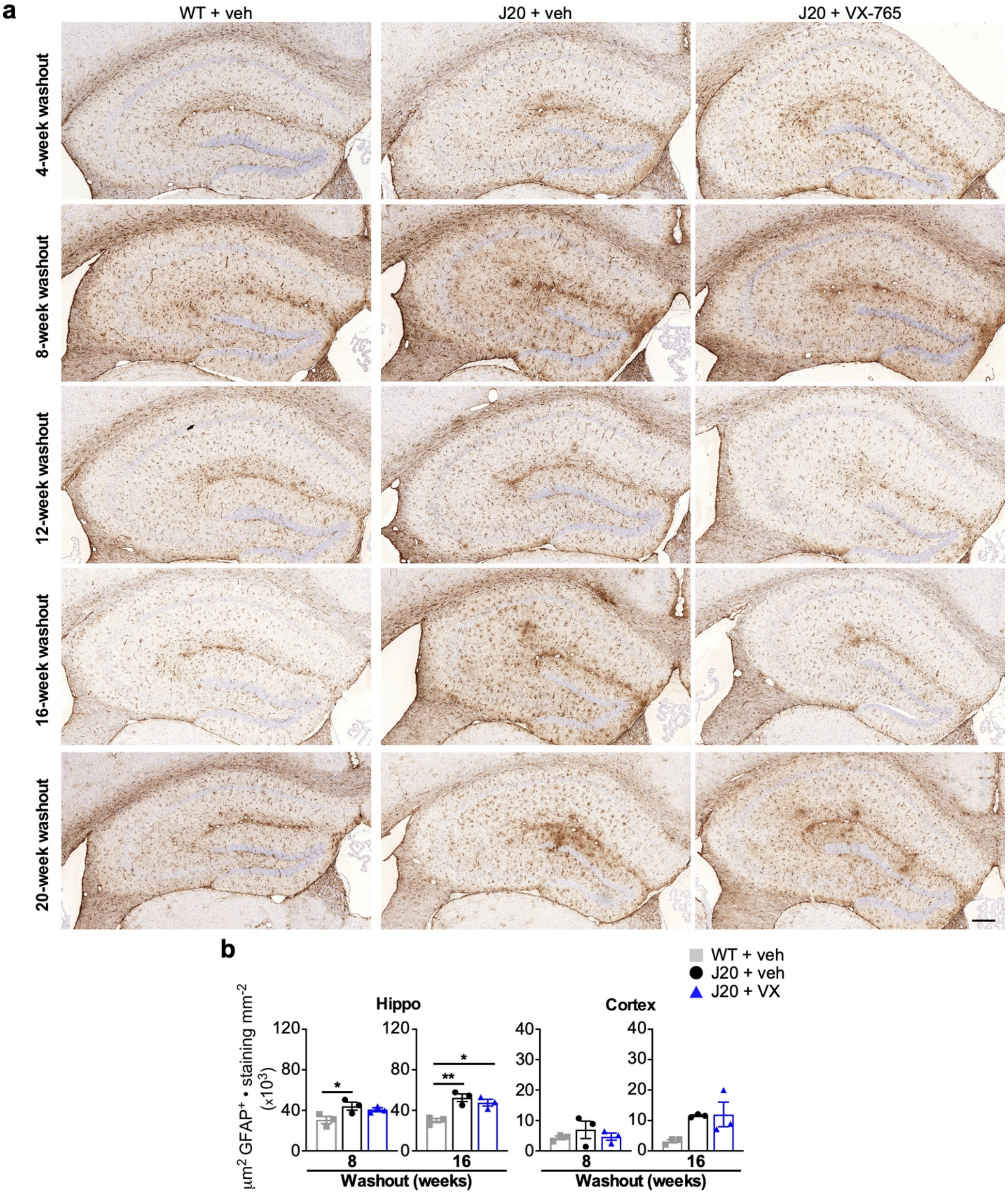

Supplementary Figure 5. Early reduction in hippocampal GFAP immunostaining in J20 mice after pre-symptomatic VX-765 treatment. (a) Representative micrographs from 3 mice brains tested per group for GFAP-positive astrogliosis in hippocampus at each WO. Scale bar = 200 μm. (b) GFAP positive

immunostaining density (n = 3 mice per group) in the CA1 hippocampus and retrosplenial and S1 cortex at 8- and 16-week WO [16-week WO hippo $F(2,6) = 13.72$, $p=0.0058$, ANOVA, Dunnett's post-hoc compared to WT + vehicle]. Data represents mean and s.e.m. *$p<0.05$, **$p<0.01$ for all figure panels. Source data are provided as a Source Data file.

Supplementary Figure 6. Flores et al., Delayed cognitive impairment in mouse model

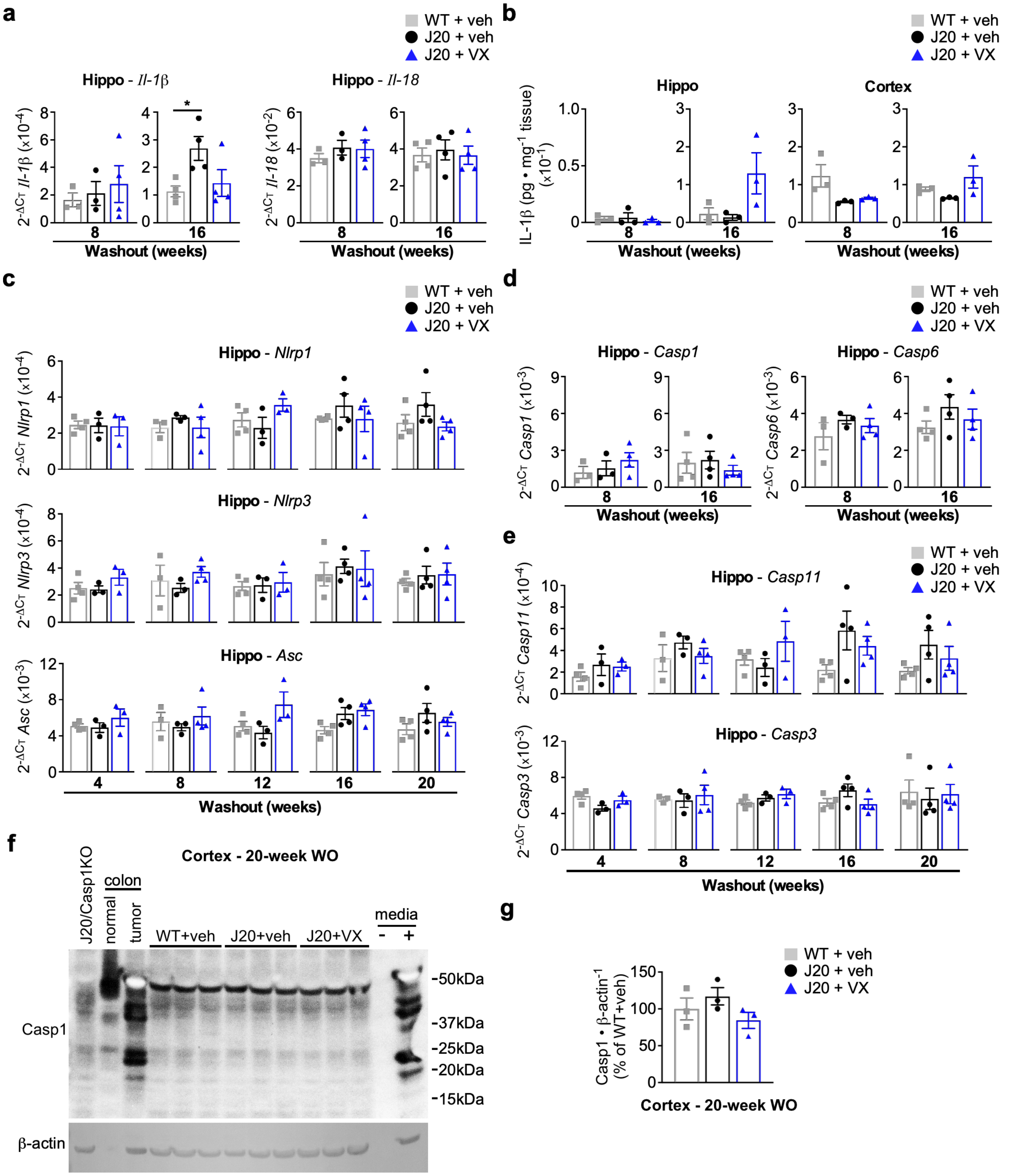

Supplementary Figure 6. Pre-symptomatic VX-765 treatment alters IL-1β but not other cytokine, inflammasome, or caspase levels in J20 mice. (a) Hippocampal *Il-1β* and *Il-18* mRNA levels at 8- and

16-week WO. (b) Total IL-1β protein levels in hippocampus and cortex (n = 3 mice per group) at 8- and 16-week WO. (c) *Nlrp1*, *Nlrp3*, and *Asc* mRNA levels in the hippocampus. (d) Hippocampal *Casp1* and *Casp6* mRNA levels at 8- and 16-week WO. (e) *Casp11* and *Casp3* mRNA levels in hippocampus. (f) Casp1 western blot (n = 3 mice per group) and (g) quantification in the cortex of vehicle-treated WT and J20 and VX-765-treated J20 mice (n = 3 mice per group). Positive controls: colon tumor from azoxymethane/dextran sulfate sodium-treated WT mouse and culture media precipitated proteins from LPS/nigericin-treated J774A.1 cells (+). Negative controls: J20/Casp1 KO cortex, normal colon from WT mouse, and culture media precipitated proteins from untreated J774A.1 cells (-). In (a) and (d) n = 3 WT + veh, 3 J20 + veh, 4 J20 + VX mice at 8-week WO; n = 4 mice per group at 16-week WO. In (c) and (e) n = 4 WT + veh, 3 J20 + veh, 3 J20 + VX at 4-week WO; n = 3 WT + veh, 3 J20 + veh, 4 J20 + VX at 8-week WO; n = 4 WT + veh, 3 J20 + veh, 3 J20 + VX at 12-week WO; n = 4 mice per group at 16-week WO; n = 4 mice per group at 20-week WO. Data represents mean and s.e.m. *$p<0.05$ for all figure panels. Source data are provided as a Source Data file.

Supplementary Figure 7. Flores et al., Delayed cognitive impairment in mouse model

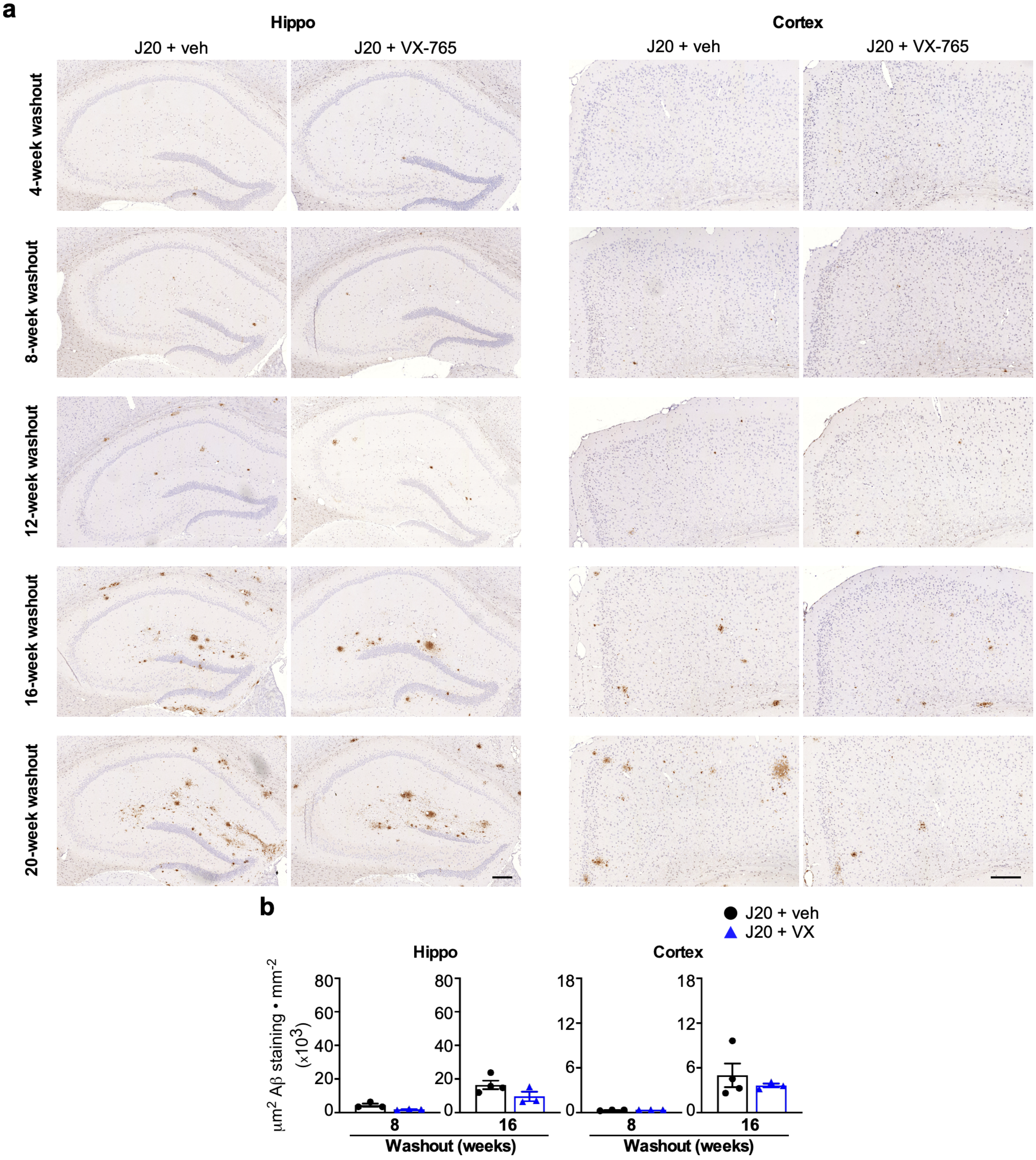

Supplementary Figure 7. Pre-symptomatic VX-765 treatment does not alter Aβ immunostaining in J20 mice. (a) Representative micrographs from mice (n is indicated in b panel) for Aβ immunostaining in hippocampus and retrosplenial area of the cortex at each WO. Scale bar = 200 µm. (b) Quantitative Aβ immunostaining density in the CA1 hippocampus and retrosplenial and S1 cortex at 8- and 16-week WO.

n = 3 mice per group at 8-week WO and n = 4 J20 + veh, 3 J20 + VX mice at 16-week WO. Data represents mean and s.e.m. Source data are provided as a Source Data file.

Supplementary Figure 8. Flores et al., Delayed cognitive impairment in mouse model

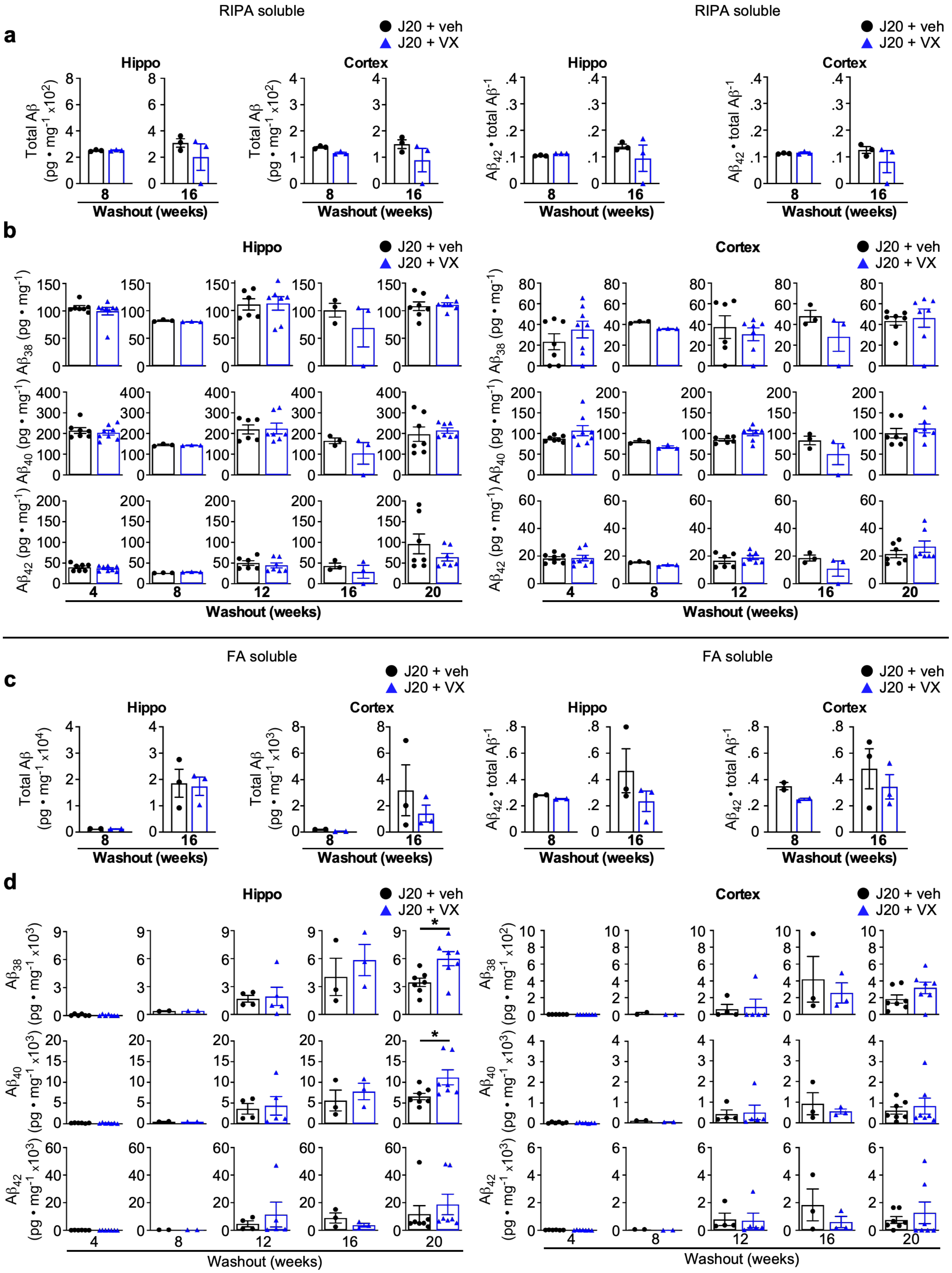

Supplementary Figure 8. Pre-symptomatic VX-765 treatment does not alter Aβ protein levels in J20 mice. RIPA-soluble (a) total Aβ and $Aβ_{42}$/total Aβ ratio in hippocampus and cortex (n = 3 mice per group) at 8- and 16-week WO, and (b) $Aβ_{38}$, $Aβ_{40}$, and $Aβ_{42}$ in hippocampus and cortex. In (b), n = 7 J20 + veh and 8 J20 + VX mice at 4-week WO; n = 3 per group at 8-week WO; n = 6 J20 + veh and 7 J20 + VX mice at 12-week WO; n = 3 mice per group at 16-week WO; n = 7 mice per group at 20-week WO. FA-soluble (c) total Aβ levels and $Aβ_{42}$/total Aβ ratio in hippocampus and cortex (n = 3 mice per group) at 8- and 16-week WO, and (d) $Aβ_{38}$, $Aβ_{40}$, and $Aβ_{42}$ levels in hippocampus and cortex. In (d),n = 6 J20 + veh and 7 J20 + VX mice at 4-week WO; n = 2 mice per group at 8-week WO; n = 4 J20 + veh and 5 J20 + VX mice at 12-week WO; n = 3 mice per group at 16-week WO; n = 7 mice per group at 20-week WO. Data represents mean and s.e.m. *$p<0.05$ for all figure panels. Source data are provided as a Source Data file.

Supplementary Figure 9. Flores et al., Delayed cognitive impairment in mouse model

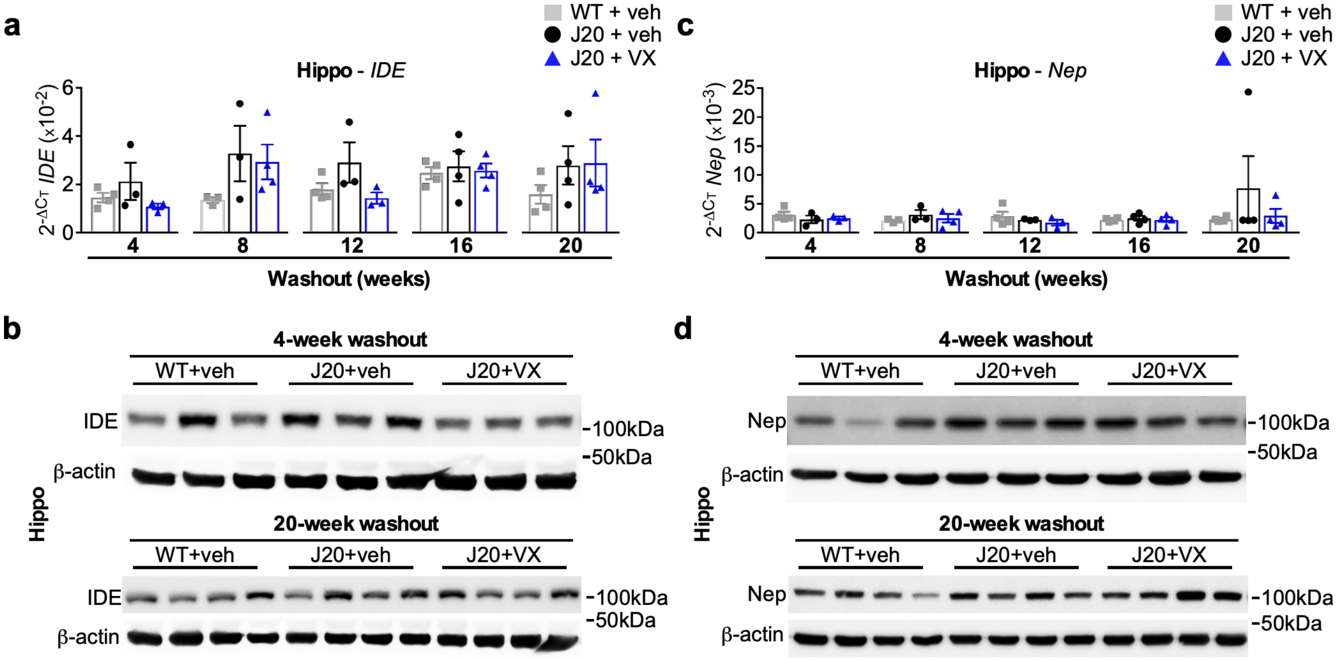

Supplementary Figure 9. Pre-symptomatic VX-765 treatment does not affect Aβ degrading enzymes. (a) *IDE* mRNA and (b) protein levels in hippocampus. (c) *Nep* mRNA and (d) protein levels in hippocampus. In (a) and (c), n = 3 mice per group at 4-week WO; n = 3 WT + veh, 3 J20 + veh, and 4 J20 + VX mice at 8-week WO; n = 3 mice per group at 12-week WO; n = 4 mice per group at 16-week WO; n = 4 mice per group at 20-week WO. Data represents mean and s.e.m. Source data are provided as a Source Data file.

Supplementary methods

Real-time PCR - primers

HPRT: *5′*-GTAATGATCAGTCAACGGGGGAC-3′; 5′-CCAGCAAGCTTGCAACCT

TAACCA-3*'*

18s: 5'-GTAACCCGTTGAACCCCAT-3'; 5'-CCATCCAATCGGTAGTAGCG-3'

hAPP: 5'-AACCAGTGACCATCCAGAAC-3'; 5'-ACTTGTCAGGAACGAGAAGG-3'

IDE: 5'-ACTAACCTGGTGGTGAAG-3'; 5'-GGTCTGGTATGGGAAATG-3'

Nep: 5'-TCTTGTAAGCAGCCTCAGCC-3'; 5'-CTCCCCACAGCATTCTCCAT-3'

Il-1β: 5'-CGGCACACCCACCCTG-3'; 5'-AAACCGCTTTTTCCATCTTCTTCT-3'

Il-18: 5'-GCTGTGACCCTCTCTGTGAA-3'; 5'-GGCAAGCAAGAAAGTGTCCT-3'

Casp11: 5'-CAACCCCACATCACTTGTCC-3'; 5'-ATGGTGGGCATCTGGGAATG-3'

Nlrp1: 5'-CACTGCCCAAGATTGCTACA-3'; 5'-CTTCACTCAGCACCAGACCA-3'

Nlrp3: 5'-GTGGTGACCCTCTGTGAGGT-3'; 5'-TCTTCCTGGAGCGCTTCTAA-3'

Asc: 5'-CCAGTGTCCCTGCTCATAGT-3'; 5'-TCATCTTGTCTTGGCTGGTG-3'

Casp3: 5'-TTCAGAGGCGACTACTGCCG-3'; 5'-ACAGGAAGTCAGCCTCCACC-3
