# Supplementary material for: Pre-symptomatic Caspase-1 inhibitor delays cognitive decline in a mouse model of Alzheimer disease and aging
Source: Nat Commun. 2020 Sep 11;11:4571. doi: 10.1038/s41467-020-18405-9 (PMC7486940; doi:10.1038/s41467-020-18405-9)
Supplement: Supplementary file 3 — Reporting Summary [file 41467_2020_18405_MOESM3_ESM.pdf]

nature research

Corresponding author(s): Andrea LeBlanc

Last updated by author(s): Aug 7, 2020

Reporting Summary

Nature Research wishes to improve the reproducibility of the work that we publish. This form provides structure for consistency and transparency in reporting. For further information on Nature Research policies, see Authors & Referees and the Editorial Policy Checklist .

Statistics

For all statistical analyses, confirm that the following items are present in the figure legend, table legend, main text, or Methods section.

n/a	Confirmed	
[ ]	[x]	The exact sample size (*n*) for each experimental group/condition, given as a discrete number and unit of measurement
[ ]	[x]	A statement on whether measurements were taken from distinct samples or whether the same sample was measured repeatedly
[ ]	[x]	The statistical test(s) used AND whether they are one- or two-sided<br>*Only common tests should be described solely by name; describe more complex techniques in the Methods section.*
[ ]	[x]	A description of all covariates tested
[ ]	[x]	A description of any assumptions or corrections, such as tests of normality and adjustment for multiple comparisons
[ ]	[x]	A full description of the statistical parameters including central tendency (e.g. means) or other basic estimates (e.g. regression coefficient) AND variation (e.g. standard deviation) or associated estimates of uncertainty (e.g. confidence intervals)
[ ]	[x]	For null hypothesis testing, the test statistic (e.g. *F*, *t*, *r*) with confidence intervals, effect sizes, degrees of freedom and *P* value noted<br>*Give P values as exact values whenever suitable.*
[x]	[ ]	For Bayesian analysis, information on the choice of priors and Markov chain Monte Carlo settings
[x]	[ ]	For hierarchical and complex designs, identification of the appropriate level for tests and full reporting of outcomes
[ ]	[x]	Estimates of effect sizes (e.g. Cohen's *d*, Pearson's *r*), indicating how they were calculated

Our web collection on statistics for biologists contains articles on many of the points above.

Software and code

Policy information about availability of computer code

Data collection	Mesoscale Discovery (MSD) Discovery Workbench 4.0.12 (LSR_4_0_12) - ELISA plate reader and analysis.<br>ImageJ 1.52q Java 1.8.9_172 (64-bit) - GFAP and AB area density. No additional custom code or plug-ins were used.<br>Dako autostainer V4.4.1 - automated histological staining<br>Mirax Scan 1.12.90.2 MIL 9 edition - digital imaging of whole brain slides
Data analysis	GraphPad Prism version 8.4.3 (471) - statistical analysis software<br>Image Gauge Science Lab 2003 Version 4.2 - western blot analysis<br>Mirax Viewer V1.12.22.1 - micrograph image viewer and analysis software

For manuscripts utilizing custom algorithms or software that are central to the research but not yet described in published literature, software must be made available to editors/reviewers. We strongly encourage code deposition in a community repository (e.g. GitHub). See the Nature Research guidelines for submitting code & software for further information.

Data

Policy information about availability of data

All manuscripts must include a data availability statement. This statement should provide the following information, where applicable:

- Accession codes, unique identifiers, or web links for publicly available datasets
- A list of figures that have associated raw data
- A description of any restrictions on data availability

The authors declare that all data of this study are available within the manuscript and its supplementary file. A source data file, including uncropped or unprocessed blots, for all relevant figures is provided with the paper. Microscope slides and/or digital scans of immunohistological staining, including access to software to view scans, are available upon request and provision of a depository site with sufficient memory to accept the files. Any additional information is available upon request.

Field-specific reporting

Please select the one below that is the best fit for your research. If you are not sure, read the appropriate sections before making your selection.

☒ Life sciences ☐ Behavioural & social sciences ☐ Ecological, evolutionary & environmental sciences

For a reference copy of the document with all sections, see nature.com/documents/nr-reporting-summary-flat.pdf

Life sciences study design

All studies must disclose on these points even when the disclosure is negative.

Sample size	Sample sizes were based on behavioural performance set in previous pilot studies showing the minimum number of mice needed to produce a deficit in the J20 experimental line.
Data exclusions	No data was excluded from the analyses
Replication	Behavioural analyses were done on 5 different cohorts within a three-year period. Each cohort was generated at different times and produced both WT and J20 mice. Therefore, our behavioural experiments are a combination of data taken from 5 separate experiments. In addition, both our immunohistochemical and biochemical experiments are combined animals from these 5 separate cohorts processed and analysed at different times. All attempts at replicating results between our different cohorts were successful
Randomization	Animals were randomly assigned to vehicle or VX-765 treatment group independent of behavioural performance. Further, animals sacrificed for immunohistological or biochemical analysis at specific washout times were chosen prior to and independent of any behavioural analyses.
Blinding	At the beginning of the experiment, animals were randomly assigned to the vehicle or VX-765 treatment independent of behavioural performance. During behavioural testing, the tester was blind to the mouse genotype and treatment group; each mouse was identified strictly by their tail markings.<br>All histological and biochemical analyses were also done blind to genotype and treatment group.

Reporting for specific materials, systems and methods

We require information from authors about some types of materials, experimental systems and methods used in many studies. Here, indicate whether each material, system or method listed is relevant to your study. If you are not sure if a list item applies to your research, read the appropriate section before selecting a response.

Materials & experimental systems

n/a	Involved in the study	
☐	☒	Antibodies
☒	☐	Eukaryotic cell lines
☒	☐	Palaeontology
☐	☒	Animals and other organisms
☒	☐	Human research participants
☒	☐	Clinical data

Methods

n/a	Involved in the study	
☒	☐	ChIP-seq
☒	☐	Flow cytometry
☒	☐	MRI-based neuroimaging

Antibodies

Antibodies used

1. Monoclonal mouse anti-beta amyloid (BioLegend 803001, Clone 6E10), Lot # B198895.

2. Polyclonal rabbit anti-amyloid precursor protein, C-terminal (Sigma A8717), Lot # 037M4808V.

3. Polyclonal rabbit anti-Iba1 (Wako 019-19741, synthetic peptide corresponding to C-terminus of Iba1), Lot # WEP0389, LKN4801, PTR2404.

4. Polyclonal rabbit anti-GFAP (Dako Z-0334), Lot # 20044021.

5. Polyclonal rabbit anti-IL-1-beta (Abcam ab9722), Lot # GR308444-1.

6. Polyclonal rabbit anti-beta amyloid 1-40 (F25276) (laboratory developed), Lot # 03/06/92 3rd boost.

7. Polyclonal rabbit anti-insulin degrading enzyme (IDE) (Abcam 32216), Lot # GR276929-1.

8. Monoclonal rabbit anti-CD10 (Neprilysin [EPR2997]) (Abcam 79423), Lot # GR313142-2.

9. Monoclonal rat anti-CD68, clone FA-11 (BioRad MCA1597), Lot #148456

10. Monoclonal rat anti-Caspase-1, clone 4B4.2.1 (Gift provided by Genentech, San Francisco, CA, USA)

11. Monoclonal mouse anti-beta actin (Sigma A5441), Lot # 115M4835V.

12. Polyclonal rabbit anti-GAPDH (Cell Signalling 2118) Lot 10.

13. HRP-conjugated goat anti-mouse secondary antibody (Jackson ImmunoResearch 115-035-00), Lot #133499

14. HRP-conjugated swine anti-rabbit secondary antibody (Dako P0217), Lot #20020160

Validation

1. Monoclonal mouse anti-beta amyloid (BioLegend 803001, Clone 6E10).
Reactivity: Human; Application: WB - Quality tested.
Validation stated on supplier's website: https://www.biolegend.com/en-us/products/purified-anti-beta-amyloid--1-16-antibody-11228

2. Polyclonal rabbit anti-amyloid precursor protein, C-terminal (Sigma A8717).
Reactivity: human, mouse, rat; Application: 1:4,000 using rat brain extract
Validation stated on supplier's website: https://www.sigmaaldrich.com/catalog/product/sigma/a8717 lang=en&region=CA&gclid=CjwKCAjw1ZbaBRBUEiwA4VQCIVRtQ1OsLLO6Sln5rXRqJxVoc4dQK6nM2wsr2MQATlhY8FfJKwBUdhoCF-gQAvD_BwE

3.Polyclonal rabbit anti-Iba1 (Wako 019-19741).
Tissue: mouse and rat hippocampus neighborhood; Section: paraffin section
Validation statement on supplier's website: http://www.wako-chem.co.jp/english/labchem/product/life/AntiIba1/index.htm

4. Polyclonal rabbit anti-GFAP (Dako Z-0334).
Validation stated on supplier's website: https://www.agilent.com/store/productDetail.jsp?catalogId=Z033429-2

5. Polyclonal rabbit anti-IL-1-beta (Abcam ab9722)
Specificity: Anti-IL-1 beta antibody is expected to react with both the mature and pro form of IL-1 beta; Suitable for: WB; Reacts with: Mouse, Rat, Humann
Validation stated on supplier's website: https://www.abcam.com/il-1-beta-antibody-ab9722.html

6. Polyclonal rabbit anti-beta amyloid 1-40 (F25276) (laboratory developed).
Refer to Golde TE, Estus S, Younkin LH, Selkoe DJ, Younkin SG, Science 255(5045), 728-30 (1992) for antibody validation.

7. Polyclonal rabbit anti-insulin degrading enzyme (IDE) (Abcam 32216).
KO Validated; Suitable for: WB; Reacts with: Mouse, Rat, Human
Validation stated on supplier's website: https://www.abcam.com/insulin-degrading-enzyme-ide-antibody-ab32216.html

8. Monoclonal rabbit anti-CD10 (Neprilysin [EPR2997]) (Abcam 79423).
Suitable for: WB; Reacts with: Mouse, Rat, Human
Validation stated on supplier's website: https://www.abcam.com/CD10-antibody-EPR2997-ab79423.html

9. Monoclonal rat anti-CD68, clone FA-11 (BioRad MCA1597)
Clone FA-11 recognizes mouse macrosialin which is a homolog of human CD68, which is classified as a unique scavenger receptor (ScR) family member. CD68 is considered a pan macrophage marker expressed on the intracellular lysosomes of tissue macrophages.
Validation done in lab (see Supplemental Figure 4) and on supplier's website:(https://www.bio-rad-antibodies.com/monoclonalmouse-cd68-antibody-fa-11-mca1957.htmlf=purified &JSESSIONID_ STERLING= 91A961E343A29FBB0 BF88390D637D1C7.ecommerce1&evCntryLang=CA-en&cntry=CA&thirdPartyCookieEnabled=true)

10. Monoclonal rat anti-Caspase-1, clone 4B4.2.1 (Gift provided by Genentech, San Francisco, CA, USA)
Validation done in lab (see Supplemental Figure 6c) and in previous publication (Heneka et al., Nature 493, 2013)

11. Monoclonal mouse anti-beta actin (Sigma A5441).
species reactivity: mouse; Application: western blot
Validation stated on supplier's website: https://www.sigmaaldrich.com/catalog/product/sigma/a5441?lang=en&region=CA&gclid=Cj0KCQiAvJXxBRCeARIsAMSkAprfna2m-MA4MLu7NhGeDlwNztlVzLBHx2QcysNDs_LRHfBZ3RLQoSAaAgbnEALw_wcB

12. Polyclonal rabbit anti-GAPDH (Cell Signalling 2118)
Validation stated on supplier's website: https://www.cellsignal.com/products/primary-antibodies/gapdh-14c10-rabbit-mab/2118?gclid=CjwKCAjw97P5BRBQEiwAGflV6VIlvFFo6ov3KhO3_ksciKJlctmspJKVznOliODFGSUeNsC2X6g5-BoC8n0QAvD_BwE&gclsrc=aw.ds&utm_campaign=prg&utm_content=gapdh+-+prg&utm_conv=mon&utm_medium=cpc&utm_region=hq&utm_source=google&utm_stage=ous&utm_strategy=lev&utm_tacti

c=ppc&utm_term=cell+signaling+2118

13. HRP-conjugated goat anti-mouse secondary antibody (Jackson ImmunoResearch 115-035-00)
Validation stated on supplier's website: https://www.jacksonimmuno.com/catalog/products/115-035-003

14. HRP-conjugated swine anti-rabbit secondary antibody (Dako P0217
Validation stated on supplier's website: https://www.agilent.com/en/product/immunohistochemistry/antibodies-controls/secondary-antibodies/swine-anti-rabbit-immunoglobulins-hrp-(solid-phase-absorbed)-153568

Animals and other organisms

Policy information about studies involving animals; ARRIVE guidelines recommended for reporting animal research

Laboratory animals	The following mouse strains were used in these studies:<br>1) B6.Cg-Zbtb20Tg(PDGFB-APPSwInd)20Lms/2Mmjax, commonly known as J20<br>2) C57BL/6J, or wild-type littermate controls<br>The J20 transgenic mice express a mutant form of the human amyloid protein precursor bearing both the Swedish (K670N/M671L) and the Indiana (V717F) mutations (APPSwInd).<br>Both J20 mice generated and their WT littermates were used alongside for our experiments. Male and female WT and J20 mice were used starting at 2 months of age and behaviourally tested anywhere from 4 to 8 months of age.
Wild animals	This study did not involve wild animals
Field-collected samples	This study did not involve field-collected samples
Ethics oversight	All study protocols and procedures followed the Canadian Council on Animal Care guidelines and approved by the McGill University Animal Care committee.

Note that full information on the approval of the study protocol must also be provided in the manuscript.
