# Supplementary material for: Pre-symptomatic Caspase-1 inhibitor delays cognitive decline in a mouse model of Alzheimer disease and aging
Source: Nat Commun. 2020 Sep 11;11:4571. doi: 10.1038/s41467-020-18405-9 (PMC7486940; doi:10.1038/s41467-020-18405-9)
Supplement: Supplementary file 4 — Source Data [file 41467_2020_18405_MOESM4_ESM.pdf]

Figure 2b - NOR DI

Figure 2c - OF distance

		NOR DI Score			Open field distance (m)		
Genotype	Treatment	4-week WO	12-week WO	20-week WO	4-week WO	12-week WO	20-week WO
WT	vehicle	0.48	-0.14	0.00	24.99	9.19	6.92
WT	vehicle	0.33	0.50	0.07	12.67	9.21	10.63
WT	vehicle	0.71	0.71	0.27	13.14	9.30	9.37
WT	vehicle	0.55	0.45	0.20	12.71	10.74	10.97
WT	vehicle	0.33	0.43	0.07	20.71	7.77	9.65
WT	vehicle	0.37	0.33	0.50	16.06	16.03	15.02
WT	vehicle	0.43	0.40	0.33	18.89	15.54	11.72
WT	vehicle	0.70	0.50	0.27	19.25	21.64	14.51
WT	vehicle	0.60	0.00	0.33	33.06	13.86	11.68
WT	vehicle	0.36	0.56	0.11	33.01	27.60	7.40
WT	vehicle	0.36	0.50	0.33	40.33	21.82	27.41
WT	vehicle	0.50	0.71	0.00	18.38	9.98	9.94
WT	vehicle	0.45	0.60	0.33	4.11	8.28	8.04
WT	vehicle	0.50	0.00	0.45	18.15	2.14	21.28
WT	vehicle	0.33	0.33	0.33	26.91	23.72	10.40
WT	vehicle	0.44	0.43	0.33	26.89	20.12	9.68
WT	vehicle	0.29	0.20	0.20	20.10	14.32	3.06
WT	vehicle	0.47	0.27	0.22	27.42	9.08	20.08
WT	vehicle	0.38	0.27	0.33	29.92	17.29	8.75
WT	vehicle	0.24	0.25	0.40	39.77	10.25	13.80
WT	vehicle	0.22	0.20	0.27	29.84	10.44	12.88
WT	vehicle	0.28	0.29	0.38	33.58	6.90	12.93
WT	vehicle	0.23	0.38	0.43	30.39	26.10	12.06
WT	vehicle	0.31	0.25	0.33	26.73	9.77	20.22
WT	VX-765	0.24	0.43	0.25	18.66	17.99	8.19
WT	VX-765	0.33	0.43	0.33	16.01	13.08	13.16
WT	VX-765	0.30	0.50	0.43	22.89	16.55	12.18
WT	VX-765	0.25	0.50	0.33	19.89	14.54	6.42
WT	VX-765	0.33	0.54	0.33	20.73	13.12	11.55
WT	VX-765	0.60	0.60	0.67	32.47	14.79	14.14
WT	VX-765	0.50	0.50	0.67	22.27	18.03	10.76
WT	VX-765	0.22	0.50	0.54	35.73	5.92	12.38
WT	VX-765	0.22	0.33	0.62	21.78	12.78	15.75
WT	VX-765	0.33	0.38	0.22	27.83	17.62	15.09
WT	VX-765	0.35	0.40	0.44	26.68	12.97	20.23
WT	VX-765	0.07	0.48	0.60	38.87	16.55	13.95
WT	VX-765	0.36	0.33	0.14	27.56	10.81	30.47
J20	vehicle	0.02	-0.05	0.45	23.16	19.72	20.08
J20	vehicle	0.00	0.03	-0.08	54.17	39.39	26.69
J20	vehicle	-0.02	0.06	0.38	29.05	27.08	21.96
J20	vehicle	0.13	-0.14	0.00	21.06	20.47	21.80
J20	vehicle	-0.04	0.11	0.04	57.68	48.48	66.58
J20	vehicle	0.05	0.03	-0.25	28.05	53.97	8.58
J20	vehicle	0.15	0.08	0.00	50.49	43.15	28.85
J20	vehicle	0.00	0.00	-0.04	32.67	47.68	33.21
J20	vehicle	0.08	-0.07	-0.11	21.82	50.48	31.38
J20	vehicle	0.00	0.04	0.07	93.08	49.59	33.40
J20	vehicle	-0.06	0.11	0.00	59.40	20.81	18.95
J20	vehicle	-0.07	-0.06	0.03	33.96	47.87	53.37
J20	vehicle	0.00	0.06	-0.20	48.32	48.30	32.89
J20	vehicle	-0.07	0.06	0.02	42.49	33.29	65.20
J20	VX-765	0.47	0.38	0.08	23.46	39.55	14.52
J20	VX-765	0.33	0.21	0.09	33.33	40.13	37.02
J20	VX-765	0.58	0.50	0.40	24.25	27.93	27.43
J20	VX-765	0.38	0.41	0.20	15.13	29.62	46.23
J20	VX-765	0.33	0.23	0.50	12.55	32.9	23.74
J20	VX-765	0.40	0.28	0.00	34.62	13.83	14.08
J20	VX-765	0.00	0.13	0.06	27.13	38.97	46.87
J20	VX-765	0.53	0.26	0.33	18.94	32.32	30.32
J20	VX-765	0.40	0.23	0.03	26.91	27.53	37.96
J20	VX-765	0.33	0.44	0.08	26.37	34.09	34.72

Figure 2d,e,f - Barnes maze 12-week WO

		Learning acquisition # or errors				Probe		Probe # of pokes														
Genotype	Treatment	Day 1	Day 2	Day 3	Day 4	Latency (s)	Errors	-9	-8	-7	-6	-5	-4	-3	-2	-1	T	+1	+2	+3	+4	+5
WT	Vehicle	5	9	6	3	9	1	0	0	0	17	0	17	17	17	17	17	0	0	0	0	0
WT	Vehicle	4	8	3	5	9	3	5	3	3	3	3	3	5	10	13	13	10	5	3	3	3
WT	Vehicle	6	3	2	2	85	19	4	8	4	8	4	4	4	8	8	13	8	4	8	4	0
WT	Vehicle	10	5	4	4	12	3	0	3	3	7	7	3	0	0	3	10	7	7	7	7	3
WT	Vehicle	5	3	6	2	60	5	3	7	7	3	0	3	7	7	14	14	10	7	0	0	0
WT	Vehicle	8	6	8	4	12	3	6	6	6	0	0	0	0	0	0	6	6	6	11	11	11
WT	Vehicle		4	1	5	8	2	6	9	6	3	6	9	9	6	12	9	6	0	3	6	0
WT	Vehicle	3	9	8	2	34	5	13	0	0	0	0	0	0	0	0	13	13	0	13	13	13
WT	Vehicle	5	10	5	3	12	2	4	4	0	4	4	0	4	13	13	13	4	4	4	9	9
WT	Vehicle	11	11	10	6	26	7	3	0	0	3	3	3	3	7	10	10	10	3	10	10	0
WT	Vehicle	7	9	5	2	17	5	0	0	0	0	5	5	5	5	15	15	10	10	5	10	5
WT	Vehicle	8	14	6	6	37	10	0	0	0	0	4	4	7	11	14	14	14	14	7	0	4
WT	Vehicle	10	4	5	3	7	0	0	0	0	5	0	5	10	10	10	10	10	5	10	5	10
WT	Vehicle	9	4	5	3	8	2	5	3	5	3	0	3	5	11	8	14	11	5	8	5	5
WT	Vehicle	8	4	4	2	13	3	0	0	0	0	0	0	0	14	14	14	14	14	14	14	0
WT	Vehicle	14	4	3	3	6	0	0	0	0	0	0	2	5	12	10	17	15	15	7	5	0
WT	Vehicle	11	5	6	4	16	5	0	0	0	7	7	7	7	7	14	14	14	14	7	0	0
WT	Vehicle	6	15	5	5	50	7	0	0	0	4	4	4	4	4	8	8	8	8	8	13	8
WT	Vehicle	10	8	8	7	34	7	0	0	3	3	3	3	6	6	10	13	13	16	3	3	3
WT	Vehicle	10	8	9	4	40	5	3	3	3	3	3	6	6	6	6	9	9	9	9	6	6
WT	Vehicle	2	3	5	1	10	2	4	4	4	4	8	4	4	8	12	12	12	8	4	4	0
WT	Vehicle	6	6	2	4	22	5	0	0	0	0	0	10	10	0	5	5	5	20	10	20	5
WT	VX-765	6	5	4	3	26	7	7	7	4	4	7	4	4	7	7	7	7	4	7	4	4
WT	VX-765	6	5	3	2	6	0	5	0	0	0	0	0	5	5	10	24	14	14	0	0	5
WT	VX-765	9	5	3	1	7	0	0	0	0	0	4	4	4	7	11	18	18	7	0	4	7
WT	VX-765	8	6	3	3	7	0	0	0	0	0	0	5	14	14	14	18	9	5	5	5	0
WT	VX-765	8	8	3	0	7	1	0	4	4	4	0	0	8	8	19	19	8	8	4	4	4
WT	VX-765	2	5	2	2	5	0	3	3	0	0	3	3	3	3	6	19	13	10	6	6	6
WT	VX-765	10	4	3	3	12	2	0	5	0	0	0	0	5	5	9	18	18	9	5	5	5
WT	VX-765	8	7	3	2	14	1	0	0	0	0	0	6	0	0	18	18	18	6	6	12	6
WT	VX-765	8	8	4	3	5	2	3	3	3	3	3	3	3	10	14	14	14	10	3	0	0
WT	VX-765	8	6	5	3	4	0	0	0	3	0	3	3	3	10	13	21	13	10	5	5	3
WT	VX-765	8	3	1	1	5	0	0	0	0	4	4	4	4	8	20	20	16	8	4	0	0
WT	VX-765	8	5	3	2	10	0	0	0	0	0	0	0	0	0	13	30	13	6	6	6	0
WT	VX-765	5	8	5	3	15	6	5	2	2	2	0	2	5	5	9	9	9	7	7	7	5
J20	vehicle	6	12	6	10	22	8	3	3	3	3	4	6	6	8	8	6	6	8	6	6	4
J20	vehicle	6	6	9	5	37	14	4	4	4	4	5	5	5	5	5	5	7	7	4	4	3
J20	vehicle	12	12	12	5	32	15	4	5	3	4	3	4	4	5	7	5	7	7	5	7	5
J20	vehicle	9	4	5	7	86	11	5	4	4	4	4	5	6	5	8	5	5	4	4	5	5
J20	vehicle			1	7	19	4	5	5	5	5	5	4	4	5	5	5	5	4	5	5	5
J20	vehicle	10	9	9	9	16	9	6	3	3	5	5	3	5	8	10	10	8	8	6	3	5
J20	vehicle	13	7	9	5	52	9	0	0	0	0	0	5	5	8	5	13	13	15	10	10	10
J20	vehicle		8	7	15	20	14	4	6	6	4	4	6	8	10	8	8	6	4	6	2	2
J20	vehicle	13	7	10	6	15	2	0	0	0	0	0	18	18	18	18	9	9	9	0	0	0
J20	vehicle	10	6	15	9	17	8	0	0	0	0	0	14	14	14	14	14	14	14	0	0	0
J20	vehicle	17	10	7	2	15	8	7	7	7	3	7	3	7	7	3	7	3	10	3	7	3
J20	vehicle	10	4	6	6	11	8	0	0	0	6	6	6	6	6	12	12	12	6	6	6	6
J20	vehicle	8	8	11	6	16	7	5	3	5	3	7	12	9	9	7	5	2	2	7	7	5
J20	vehicle	13	13	11	7	15	15	0	0	0	0	0	0	9	9	9	9	4	9	9	9	9
J20	vehicle	6	8	5	3	5	1	0	0	6	6	6	6	6	6	12	12	12	12	6	0	0
J20	VX-765		9	5	4	20	3	0	0	0	0	0	13	25	0	0	13	13	13	13	13	0
J20	VX-765	10	10	12	5	13	3	0	0	2	4	4	2	6	13	11	19	7	9	7	6	6
J20	VX-765	11	11	11	3	28	4	2	2	5	5	7	7	7	7	5	7	5	5	5	5	5
J20	VX-765	3	4	9	2	9	3	2	5	5	0	5	7	2	7	7	10	10	10	7	5	7
J20	VX-765	14	7	8	4	20	4	3	3	6	0	3	14	14	6	6	9	9	9	9	6	0
J20	VX-765	4	7	15	4	19	5	2	2	2	2	7	7	2	5	10	10	7	10	12	10	5
J20	VX-765	2	3	6	4	10	2	0	0	0	0	5	5	9	14	9	14	9	9	9	5	5
J20	VX-765	4	8	5	6	25	4	2	2	3	3	5	2	5	11	11	11	8	8	6	5	5
J20	VX-765	8	14	10	3	12	5	2	0	4	2	2	2	6	10	12	8	10	8	8	8	8
J20	VX-765	12	9	8	3	17	2	4	4	6	0	4	6	8	8	8	8	8	6	4	4	2

Figure 2g,h,i - Barnes maze 20-week WO

						Learning acquisition # or errors				Probe		Probe # of pokes																		
+6	+7	+8	+9	Genotype	Treatment	Day 1	Day 2	Day 3	Day 4	Latency (s)	Errors	-9	-8	-7	-6	-5	-4	-3	-2	-1	T	+1	+2	+3	+4	+5	+6	+7	+8	+9
0	0	0	0	WT	Vehicle	7	2	3	1	10	0	0	0	0	0	0	0	0	15	15	15	15	15	8	8	0	0	0	0	8
3	3	5	3	WT	Vehicle	11	3	4	1	15	4	3	6	6	3	0	6	6	13	13	13	9	3	3	3	3	0	3	3	0
4	0	4	0	WT	Vehicle	9	3	3	2	27	3	18	9	0	0	0	0	0	9	9	18	9	0	0	0	9	0	0	0	9
7	7	7	7	WT	Vehicle	12	6	4	3	8	0	10	5	0	5	5	5	5	0	10	18	15	10	5	0	0	0	0	5	5
0	7	3	3	WT	Vehicle	2	5	3	2	14	3	0	0	0	0	0	6	17	11	17	20	17	6	6	6	0	0	0	0	0
6	11	11	0	WT	Vehicle	11	9	6	4	10	2	5	5	5	5	0	5	5	9	9	17	14	9	0	5	5	5	0	0	0
0	0	9	0	WT	Vehicle	9	5	6	3	10	2	3	5	5	5	5	5	5	5	5	8	8	8	5	3	0	5	5	5	0
13	13	0	0	WT	Vehicle	7	10	5	5	8	0	3	3	3	3	3	3	3	3	6	26	16	6	3	3	0	3	3	3	3
4	4	0	0	WT	Vehicle	6	13	5	6	18	3	3	0	0	0	0	3	13	9	9	9	6	6	9	6	9	3	3	3	3
3	3	3	7	WT	Vehicle	8	5	4	3	12	2	5	5	5	5	5	5	5	5	5	9	9	7	5	3	2	3	3	3	3
5	5	0	0	WT	Vehicle	8	7	3	2	9	0	0	0	0	0	0	0	0	0	0	10	0	0	0	0	0	0	0	0	0
4	4	0	0	WT	Vehicle	5	6	2	2	10	3	4	4	0	0	4	4	7	7	11	14	11	14	4	0	0	4	7	7	0
5	5	0	0	WT	Vehicle	7	4	3	2	40	4	0	0	0	0	0	0	0	15	15	23	25	10	0	0	0	0	5	5	5
0	5	3	0	WT	Vehicle	10	6	3	4	12	3	6	3	0	6	3	6	3	12	12	15	9	9	3	3	0	0	3	3	6
0	0	0	0	WT	Vehicle	7	5	4	3	21	5	5	5	0	0	0	0	11	16	11	16	11	0	5	5	5	0	0	5	5
5	5	2	0	WT	Vehicle	7	8	2	4	8	0	0	0	0	0	0	0	0	5	10	13	17	17	10	5	0	10	10	10	5
0	0	0	0	WT	Vehicle	5	8	3	4	8	3	0	0	0	0	0	0	0	0	0	30	30	30	0	0	0	0	0	0	0
4	4	4	4	WT	Vehicle	7	9	4	3	8	0	7	3	7	7	0	7	7	7	7	10	10	7	7	3	7	3	0	0	0
3	3	3	3	WT	Vehicle	8	5	2	4	8	3	4	4	7	7	7	11	7	4	7	11	7	4	11	0	0	0	4	4	0
6	3	3	3	WT	Vehicle	9	3	6	2	6	0	0	0	0	0	4	4	4	8	8	8	8	8	8	12	15	8	8	0	0
0	4	0	4																											
5	5	0	0																											
0	4	4	4	WT	VX-765	9	3	4	1	9	2	3	3	0	0	3	0	3	6	25	25	13	6	3	3	3	0	0	0	0
10	10	0	0	WT	VX-765	9	4	5	4	10	3	0	7	7	7	3	3	7	7	10	14	10	7	7	3	0	0	0	3	0
4	4	4	4	WT	VX-765	8	1	3	1	6	0	0	6	6	0	3	6	10	10	16	16	10	10	0	0	0	0	3	3	0
5	5	5	0	WT	VX-765	2	4	7	0	7	0	4	4	0	0	0	0	12	12	12	15	12	12	4	4	4	4	4	0	0
4	4	0	0	WT	VX-765	5	4	4	3	7	1	4	4	8	8	4	0	8	12	16	16	12	4	0	0	4	0	0	0	0
6	6	0	0	WT	VX-765	7	5	3	2	8	0	3	3	0	0	0	3	3	6	12	21	18	6	6	3	3	3	3	3	0
5	5	5	0	WT	VX-765	10	3	5	1	6	0	0	4	4	0	9	9	9	9	9	13	13	13	4	0	4	0	0	0	0
0	0	6	6	WT	VX-765	9	2	1	3	9	1	0	6	6	0	0	0	0	6	19	25	19	6	6	6	0	0	0	0	0
0	3	3	0	WT	VX-765	4	3	6	3	6	0	5	5	5	5	0	0	0	5	10	10	10	5	5	5	5	5	5	5	5
0	5	3	0	WT	VX-765	10	4	1	2	6	1	2	2	2	5	2	5	5	7	9	16	14	9	7	7	2	2	0	2	0
0	0	0	4	WT	VX-765	3	4	2	1	6	0	4	0	4	4	4	4	4	4	8	27	19	8	0	0	4	4	0	0	4
6	13	0	0	WT	VX-765	8	5	2	1	9	2	3	0	0	3	0	5	5	8	14	16	14	8	5	5	3	0	3	5	0
5	7	5	2	WT	VX-765	5	4	3	5	8	4	4	6	6	6	6	6	8	8	8	8	6	4	4	0	4	4	0	2	6
5	6	4	4	J20	vehicle	7	3	10	5	13	5	4	6	4	4	4	5	6	6	6	6	5	4	4	5	6	5	4	4	4
4	7	7	5	J20	vehicle	7	7	6	2	18	3	0	2	2	6	8	6	6	6	8	10	8	10	4	2	2	8	4	6	2
7	5	4	5	J20	vehicle	17	5	6	4	8	4	5	2	0	3	2	5	5	8	9	8	8	6	8	5	6	3	8	6	5
5	6	6	6	J20	vehicle	10	9	6	11	60	2	4	4	4	4	4	4	4	8	8	8	8	8	4	4	4	4	4	4	4
5	5	5	5	J20	vehicle	11	8	7	6	20	10	5	5	4	1	2	4	5	7	8	10	7	8	5	5	5	5	5	5	5
3	3	3	3	J20	vehicle	4	11	9	7	90	12	7	7	7	7	0	0	0	7	7	7	0	7	7	7	7	7	7	7	0
3	0	3	3	J20	vehicle	15	5	2	4	18	11	5	5	6	4	6	7	7	5	5	5	4	6	7	5	5	5	5	4	5
2	2	4	4	J20	vehicle	10	10	13	5	15	7	8	8	8	8	0	0	0	0	0	0	0	8	8	8	8	8	8	8	8
0	0	0	0	J20	vehicle	4	12	8	11	25	11	5	5	4	6	6	6	5	5	6	6	4	6	6	5	3	4	5	5	5
0	0	0	0	J20	vehicle	9	7	10	10	10	12	4	4	4	4	1	3	4	7	10	10	8	7	4	6	7	6	3	3	6
3	3	3	7	J20	vehicle	10	9	6	10	18	7	3	4	4	6	6	6	7	7	8	8	8	7	9	6	5	5	3	3	3
6	6	0	0																											
3	5	0	3																											
9	9	4	4																											
6	6	0	0																											
0	0	0	0	J20	VX-765	5	5	3	0	13	4	5	0	0	0	0	7	13	13	18	18	7	5	4	2	0	0	0	4	5
6	0	0	0	J20	VX-765	8	9	8	5	23	5	4	2	5	4	5	7	9	4	7	7	7	7	4	5	4	4	7	4	4
5	5	5	5	J20	VX-765	8	6	5	5	13	3	0	7	2	5	5	2	5	7	10	10	12	10	5	5	0	5	5	5	2
5	2	2	0	J20	VX-765	9	4	6	5	12	2	6	4	0	4	4	4	6	8	8	9	8	4	8	6	8	8	4	4	2
0	3	0	3	J20	VX-765	11	5	3	5	27	5	4	7	7	4	4	11	7	4	7	11	7	7	0	0	4	4	4	4	7
2	0	2	2	J20	VX-765	10	8	7	9	32	10	4	2	2	2	2	6	9	6	6	8	4	8	6	8	8	6	4	6	8
5	5	0	0	J20	VX-765	8	9	5	3	17	4	6	7	3	3	1	3	3	4	6	9	9	6	6	6	7	6	4	6	4
2	2	8	2																											
4	2	2	4																											
4	6	6	4																											

Figure 3a - hAPP qPCR

				hAPP hippo		
Genotype	Treatment	WO (Weeks)	Mean Ct hAPP	Mean Ct HPRT+18s	ΔCt	$2^{-\Delta Ct}$
J20	Vehicle	4	13.77496719	15.37090969	-1.595942497	**3.02292**
J20	Vehicle	4	14.02191353	15.28404999	-1.262136459	**2.39851**
J20	Vehicle	4	13.92167664	14.81592607	-0.894249439	**1.85864**
J20	VX-765	4	14.28224945	15.5962553	-1.314005852	**2.48631**
J20	VX-765	4	13.58196259	15.2533884	-1.671425819	**3.18529**
J20	VX-765	4	14.04699707	15.36834908	-1.321352005	**2.49900**
J20	Vehicle	12	14.69730282	15.21114445	-0.513841629	**1.42785**
J20	Vehicle	12	12.92142296	15.20016241	-2.278739452	**4.85254**
J20	Vehicle	12	13.13781548	14.65073061	-1.512915134	**2.85386**
J20	VX-765	12	13.74358749	15.46437502	-1.720787525	**3.29616**
J20	VX-765	12	14.1049614	15.16145897	-1.056497574	**2.07988**
J20	VX-765	12	13.53317165	15.32771206	-1.794540405	**3.46905**
J20	Vehicle	20	13.50344276	15.21928406	-1.715841293	**3.28488**
J20	Vehicle	20	13.07200432	15.23942089	-2.167416573	**4.49218**
J20	Vehicle	20	14.05902958	15.38573456	-1.326704979	**2.50829**
J20	Vehicle	20	14.18771362	15.03895283	-0.851239204	**1.80405**
J20	VX-765	20	13.5593071	15.04809523	-1.488788128	**2.80653**
J20	VX-765	20	13.30195999	15.19388771	-1.891927719	**3.71131**
J20	VX-765	20	14.26202679	15.09176922	-0.829742432	**1.77737**
J20	VX-765	20	13.72371483	15.5668292	-1.843114376	**3.58784**

Figure 3b - hAPP western blot in the hippocampus

Antibody: 6E10, Biolegend,803001, lot B198895 (1:1000)

hAPP at 4-week WO

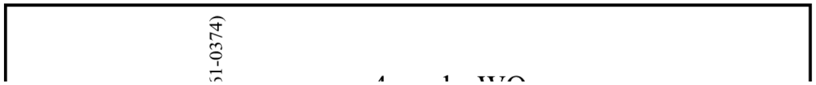

hAPP at 20-week WO

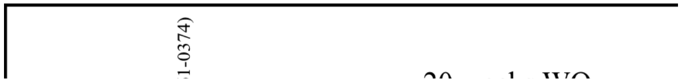

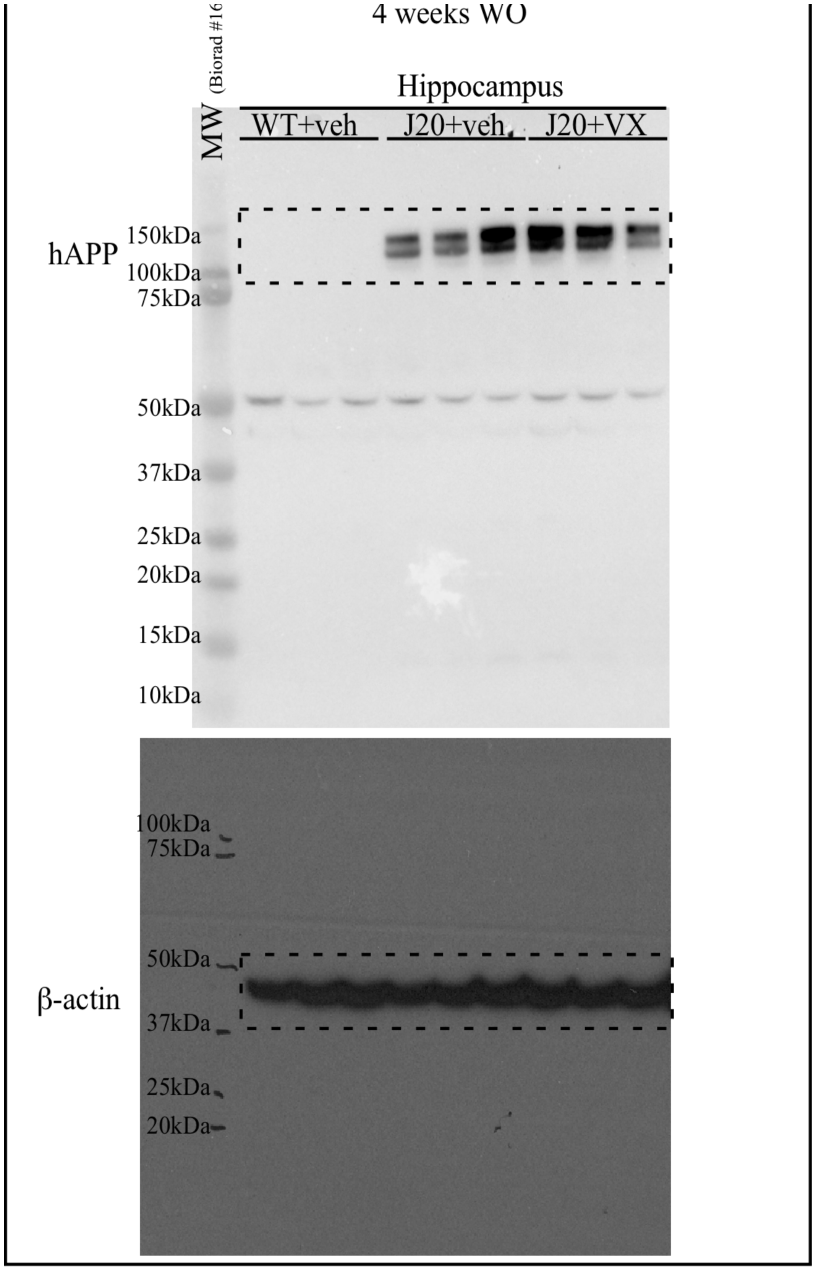

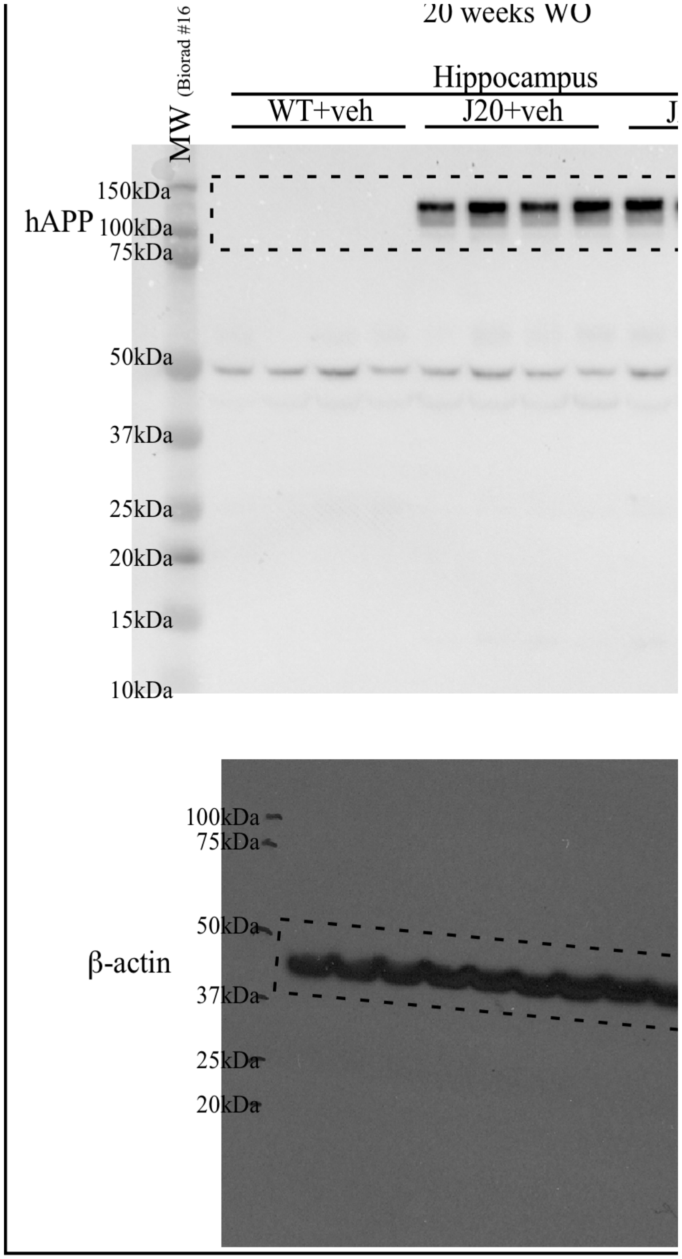

*Dotted area is what is shown in publication figure

Figure 3c - hAPP western blot quantification (% of J20+veh)

Hippo	J20+veh	J20+VX
4-week WO	**83.1**	**144.7**
	**77.5**	**137.0**
	**139.4**	**71.5**
	**113.1**	**89.5**
	**124.7**	**97.0**

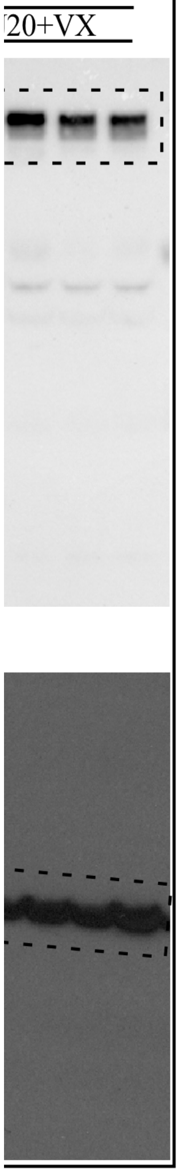

	62.8	113.8
	99.3	
20-week WO	94.2	117.8
	113.6	114.3
	81.9	95.0
	110.4	80.6
	97.6	102.7
	99.3	83.6
	103.1	83.7

Figure 4a & b - Iba1 quantification and morphological analysis

Hippo	Genotype	Treatment	TOTAL # Iba1	Total Volume Analyzed (mm3)	**CELL DENSITY (cells/mm3)**	Iba-1 Distribution (#) i	ii	iii	iv
4-week WO	WT	Vehicle	176	0.0308	**5723.2**	125	50	1	0
	WT	Vehicle	194	0.0387	**5014.5**	138	54	2	0
	WT	Vehicle	276	0.0476	**5796.4**	195	80	0	0
	WT	Vehicle	168	0.0252	**6659.2**	149	17	2	0
	WT	Vehicle	156	0.0252	**6183.6**	146	10	0	0
	WT	Vehicle	182	0.0252	**7214.2**	140	42	0	0
	WT	Vehicle	140	0.0252	**5549.4**	136	4	0	0
	J20	Vehicle	266	0.0327	**8125.6**	56	175	35	0
	J20	Vehicle	169	0.0218	**7743.8**	37	106	26	0
	J20	Vehicle	194	0.0218	**8889.3**	38	138	18	0
	J20	Vehicle	230	0.0252	**9116.8**	170	60	0	0
	J20	Vehicle	136	0.0126	**10781.6**	102	33	1	0
	J20	Vehicle	254	0.0252	**10068.1**	216	34	4	0
	J20	Vehicle	226	0.0252	**8958.2**	158	62	6	0
	J20	VX-765	159	0.0357	**4452.3**	101	52	4	2
	J20	VX-765	156	0.0357	**4368.3**	76	64	4	12
	J20	VX-765	120	0.0238	**5040.3**	73	41	2	4
	J20	VX-765	184	0.0252	**7293.4**	140	40	0	4
	J20	VX-765	214	0.0252	**8482.6**	176	34	4	0
	J20	VX-765	144	0.0252	**5707.9**	108	34	2	0
	J20	VX-766	168	0.0252	**6659.2**	144	22	2	0
12-week WO	WT	Vehicle	194	0.0456	**4251.4**	150	40	4	0
	WT	Vehicle	203	0.0516	**3935.3**	166	33	4	0
	WT	Vehicle	154	0.0327	**4704.3**	120	30	4	0
	WT	Vehicle	152	0.0252	**6025.0**	146	4	2	0
	WT	Vehicle	176	0.0252	**6976.3**	144	28	4	0
	WT	Vehicle	170	0.0252	**6738.5**	134	34	2	0

	WT	Vehicle	170	0.0252	**6738.5**	134	36	0	0
	J20	Vehicle	294	0.0412	**7141.5**	140	137	17	0
	J20	Vehicle	210	0.0278	**7560.5**	94	112	4	0
	J20	Vehicle	189	0.0139	**13608.9**	93	81	15	0
	J20	Vehicle	155	0.0126	**12287.9**	123	25	7	0
	J20	Vehicle	140	0.0126	**11098.7**	84	42	14	0
	J20	Vehicle	121	0.0126	**9592.5**	76	38	7	0
	J20	Vehicle	131	0.0126	**10385.2**	72	44	15	0
	J20	VX-765	246	0.0486	**5060.9**	159	77	9	1
	J20	VX-765	152	0.0268	**5675.0**	80	66	6	0
	J20	VX-765	240	0.0436	**5498.5**	172	64	4	0
	J20	VX-765	230	0.0252	**9116.8**	138	68	24	0
	J20	VX-765	192	0.0252	**7610.5**	132	52	8	0
	J20	VX-765	186	0.0252	**7372.7**	72	88	26	0
	J20	VX-766	210	0.0252	**8324.0**	146	52	12	0
20-week WO	WT	Vehicle	232	0.0288	**8055.6**	176	56	0	0
	WT	Vehicle	180	0.0288	**6250.0**	136	40	4	0
	WT	Vehicle	228	0.0288	**7916.7**	156	44	28	0
	WT	Vehicle	180	0.0252	**7134.9**	126	54	0	0
	WT	Vehicle	194	0.0252	**7689.8**	152	42	0	0
	WT	Vehicle	160	0.0252	**6342.1**	108	52	0	0
	WT	Vehicle	198	0.0252	**7848.4**	144	54	0	0
	J20	Vehicle	182	0.0144	**12638.9**	100	74	8	0
	J20	Vehicle	174	0.0144	**12083.3**	54	74	46	0
	J20	Vehicle	200	0.0144	**13888.9**	74	86	40	0
	J20	Vehicle	150	0.0126	**11891.5**	58	59	33	0
	J20	Vehicle	160	0.0126	**12684.2**	45	100	15	0
	J20	VX-765	176	0.0223	**7885.3**	102	30	32	12
	J20	VX-765	228	0.0223	**10215.1**	86	64	58	20
	J20	VX-765	292	0.0248	**11774.2**	88	76	100	28
	J20	VX-765	230	0.0252	**9116.8**	82	100	36	12
	J20	VX-765	232	0.0252	**9196.1**	140	70	16	6

Figure 4c - GFAP western blot in the hippocampus

Antibody: GFAP, Dako, z0334, lot 20044021 (1:3000)

GFAP at 4-week WO

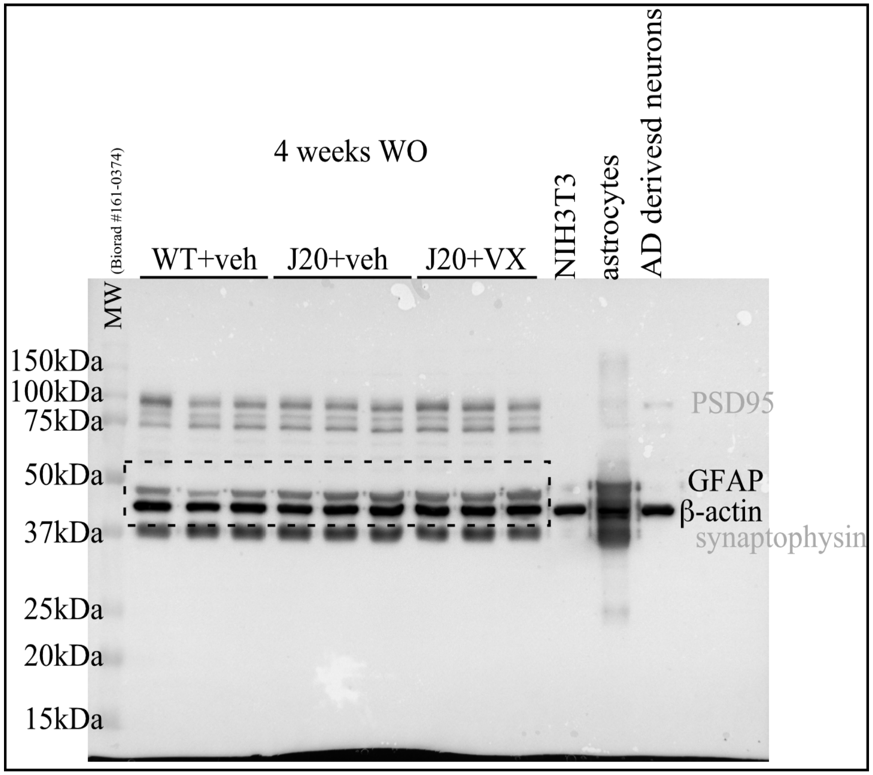

GFAP at 20-week WO

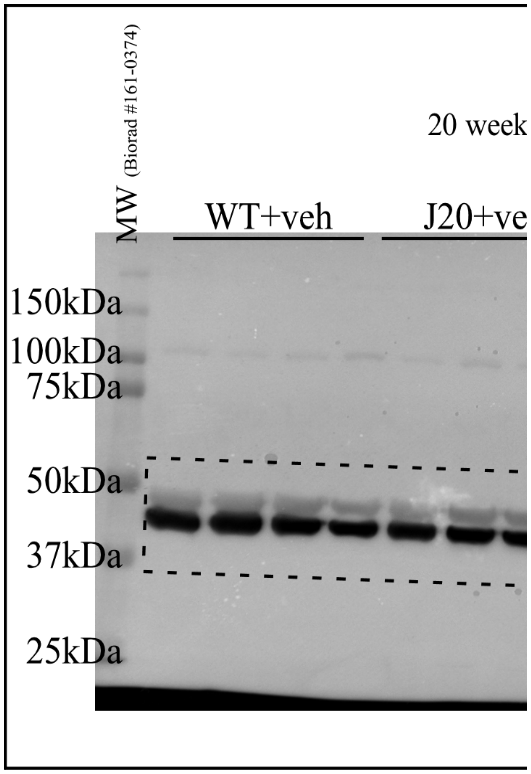

*Dotted area is what is shown in publication figure

Figure 4e GFAP density quantification

Hippo	Genotype	Treatment	SUM/Total Stained Area (um2)	SUM/Total Area Analyzed (converted to mm^2)	**SUM Surface Area Staining (um^2 staining/mm^2)**
4-week WO	WT	Vehicle	59000.0	2.355	**25056.8**
	WT	Vehicle	75214.0	2.355	**31942.7**
	WT	Vehicle	54937.0	2.355	**23331.3**
	WT	Vehicle	66116.0	2.355	**28078.9**
	WT	Vehicle	53705.0	2.260	**23762.0**
	WT	Vehicle	73598.0	2.260	**32563.8**
	WT	Vehicle	61813.0	2.260	**27349.5**
	J20	Vehicle	100303	2.355	**42597.8**
	J20	Vehicle	118068	2.355	**50142.5**
	J20	Vehicle	134961	2.355	**57316.8**
	J20	Vehicle	217923	2.355	**92550.0**
	J20	Vehicle	96066	2.260	**42504.9**
	J20	Vehicle	102026	2.260	**45141.9**
	J20	Vehicle	102389	2.260	**45302.5**
	J20	VX-765	112323	2.355	**47702.6**
	J20	VX-765	72557	2.355	**30814.3**
	J20	VX-765	148291	2.355	**62977.9**
	J20	VX-765	79397	2.355	**33719.2**
	J20	VX-765	64750	2.260	**28648.9**
	J20	VX-765	86294	2.260	**38181.2**
	J20	VX-765	80564	2.260	**35645.9**
12-week WO	WT	Vehicle	47455	2.355	**20153.7**
	WT	Vehicle	58836	2.355	**24987.1**
	WT	Vehicle	77488	2.355	**32908.5**
	WT	Vehicle	68627	2.355	**29145.3**
	WT	Vehicle	67517	2.260	**29873.2**
	WT	Vehicle	38105	2.260	**16859.7**
	WT	Vehicle	98400	2.260	**43537.5**

Cortex	Genotype	Treatment
4-week WO	WT	Vehicle
	WT	Vehicle
	WT	Vehicle
	WT	Vehicle
	WT	Vehicle
	WT	Vehicle
	WT	Vehicle
	J20	Vehicle
	J20	Vehicle
	J20	Vehicle
	J20	Vehicle
	J20	Vehicle
	J20	Vehicle
	J20	Vehicle
	J20	VX-765
	J20	VX-765
	J20	VX-765
	J20	VX-765
	J20	VX-765
	J20	VX-765
	J20	VX-765
12-week WO	WT	Vehicle
	WT	Vehicle
	WT	Vehicle
	WT	Vehicle
	WT	Vehicle
	WT	Vehicle
	WT	Vehicle

	J20	Vehicle	94965	2.355	**40330.8**		J20	Vehicle
	J20	Vehicle	126300	2.355	**53638.5**		J20	Vehicle
	J20	Vehicle	120926	2.355	**51356.2**		J20	Vehicle
	J20	Vehicle	181133	2.355	**76925.6**		J20	Vehicle
	J20	Vehicle	116082	2.260	**51361.0**		J20	Vehicle
	J20	Vehicle	118251	2.260	**52320.7**		J20	Vehicle
	J20	Vehicle	136549	2.260	**60416.8**		J20	Vehicle
	J20	VX-765	101708	2.260	**45001.2**		J20	VX-765
	J20	VX-765	73953	2.260	**32720.9**		J20	VX-765
	J20	VX-765	88700	2.260	**39245.7**		J20	VX-765
	J20	VX-765	123232	2.355	**52335.6**		J20	VX-765
	J20	VX-765	87366	2.355	**37103.6**		J20	VX-765
	J20	VX-765	140744	2.355	**59772.8**		J20	VX-765
	J20	VX-765	66260	2.355	**28140.1**		J20	VX-765
20-week WO	WT	Vehicle	63375	2.260	**28040.6**	20-week WO	WT	Vehicle
	WT	Vehicle	52853	2.260	**23385.1**		WT	Vehicle
	WT	Vehicle	62933	2.260	**27845.0**		WT	Vehicle
	WT	Vehicle	64616	2.355	**27441.9**		WT	Vehicle
	WT	Vehicle	72701	2.355	**30875.5**		WT	Vehicle
	WT	Vehicle	78282	2.355	**33245.7**		WT	Vehicle
	WT	Vehicle	75248	2.355	**31957.2**		WT	Vehicle
	J20	Vehicle	240124	2.355	**101978.6**		J20	Vehicle
	J20	Vehicle	268404	2.355	**113988.9**		J20	Vehicle
	J20	Vehicle	111234	2.260	**49216.0**		J20	Vehicle
	J20	Vehicle	112210	2.260	**49647.9**		J20	Vehicle
	J20	Vehicle	117184	2.260	**51848.6**		J20	Vehicle
	J20	VX-765	172187	2.260	**76185.0**		J20	VX-765
	J20	VX-765	79679	2.260	**35254.4**		J20	VX-765
	J20	VX-765	115959	2.260	**51306.6**		J20	VX-765
	J20	VX-765	188095	2.355	**79882.3**		J20	VX-765
	J20	VX-765	225818	2.355	**95903.0**		J20	VX-765

Iba-1 Distribution (%)									
i	ii	iii	iv	Cortex	Genotype	Treatment	TOTAL # Iba1	Total Volume Analyzed (mm3)	CELL DENSITY (cells/mm3)
**71%**	**28%**	**1%**	**0%**	4-week WO	WT	Vehicle	190	0.0198	**9576.6**
**71%**	**28%**	**1%**	**0%**		WT	Vehicle	108	0.0198	**5443.5**
**71%**	**29%**	**0%**	**0%**		WT	Vehicle	149	0.0223	**6675.6**
**89%**	**10%**	**1%**	**0%**		WT	Vehicle	188	0.0252	**7452.0**
**94%**	**6%**	**0%**	**0%**		WT	Vehicle	172	0.0252	**6817.8**
**77%**	**23%**	**0%**	**0%**		WT	Vehicle	166	0.0252	**6579.9**
**97%**	**3%**	**0%**	**0%**		WT	Vehicle	154	0.0252	**6104.3**
**21%**	**66%**	**13%**	**0%**		J20	Vehicle	133	0.0099	**13407.3**
**22%**	**63%**	**15%**	**0%**		J20	Vehicle	230	0.0174	**13248.8**
**20%**	**71%**	**9%**	**0%**		J20	Vehicle	248	0.0198	**12500.0**
**74%**	**26%**	**0%**	**0%**		J20	Vehicle	278	0.0252	**11019.4**
**75%**	**24%**	**1%**	**0%**		J20	Vehicle	272	0.0252	**10781.6**
**85%**	**13%**	**2%**	**0%**		J20	Vehicle	260	0.0252	**10305.9**
**70%**	**27%**	**3%**	**0%**		J20	Vehicle	218	0.0252	**8641.1**
**64%**	**33%**	**3%**	**1%**		J20	VX-765	108	0.0198	**5443.5**
**49%**	**41%**	**3%**	**8%**		J20	VX-765	138	0.0198	**6955.6**
**61%**	**34%**	**2%**	**3%**		J20	VX-765	182	0.0198	**9173.4**
**76%**	**22%**	**0%**	**2%**		J20	VX-765	204	0.0252	**8086.2**
**82%**	**16%**	**2%**	**0%**		J20	VX-765	214	0.0252	**8482.6**
**75%**	**24%**	**1%**	**0%**		J20	VX-765	194	0.0252	**7689.8**
**86%**	**13%**	**1%**	**0%**		J20	VX-766	168	0.0252	**6659.2**
**77%**	**21%**	**2%**	**0%**	12-week WO	WT	Vehicle	127	0.0298	**4267.5**
**82%**	**16%**	**2%**	**0%**		WT	Vehicle	147	0.0298	**4939.5**
**78%**	**19%**	**3%**	**0%**		WT	Vehicle	208	0.0298	**6989.2**
**96%**	**3%**	**1%**	**0%**		WT	Vehicle	73	0.0126	**5787.2**
**82%**	**16%**	**2%**	**0%**		WT	Vehicle	80	0.0126	**6342.1**
**79%**	**20%**	**1%**	**0%**		WT	Vehicle	80	0.0126	**6342.1**

**79%**	**21%**	**0%**	**0%**		WT	Vehicle	94	0.0126	**7452.0**
**48%**	**47%**	**6%**	**0%**		J20	Vehicle	134	0.0126	**10623.0**
**45%**	**53%**	**2%**	**0%**		J20	Vehicle	149	0.0126	**11812.2**
**49%**	**43%**	**8%**	**0%**		J20	Vehicle	121	0.0126	**9592.5**
**79%**	**16%**	**5%**	**0%**		J21	Vehicle	128	0.0126	**10147.4**
**60%**	**30%**	**10%**	**0%**		J20	Vehicle	230	0.0198	**11592.7**
**63%**	**31%**	**6%**	**0%**		J20	Vehicle	236	0.0198	**11895.2**
**55%**	**34%**	**11%**	**0%**		J20	Vehicle	206	0.0198	**10383.1**
**65%**	**31%**	**4%**	**0%**		J20	VX-765	160	0.0298	**5376.3**
**53%**	**43%**	**4%**	**0%**		J20	VX-765	154	0.0198	**7762.1**
**72%**	**27%**	**2%**	**0%**		J20	VX-765	240	0.0397	**6048.4**
**60%**	**30%**	**10%**	**0%**		J20	VX-765	101	0.0126	**8006.9**
**69%**	**27%**	**4%**	**0%**		J20	VX-765	100	0.0126	**7927.6**
**39%**	**47%**	**14%**	**0%**		J20	VX-765	110	0.0126	**8720.4**
**70%**	**25%**	**6%**	**0%**		J20	VX-766	95	0.0126	**7531.3**
**76%**	**24%**	**0%**	**0%**	20-week WO	WT	Vehicle	156	0.0180	**8666.7**
**76%**	**22%**	**2%**	**0%**		WT	Vehicle	142	0.0180	**7888.9**
**68%**	**19%**	**12%**	**0%**		WT	Vehicle	120	0.0180	**6666.7**
**70%**	**30%**	**0%**	**0%**		WT	Vehicle	99	0.0126	**7848.4**
**78%**	**22%**	**0%**	**0%**		WT	Vehicle	97	0.0126	**7689.8**
**68%**	**33%**	**0%**	**0%**		WT	Vehicle	95	0.0126	**7531.3**
**73%**	**27%**	**0%**	**0%**		WT	Vehicle	98	0.0126	**7769.1**
**55%**	**41%**	**4%**	**0%**		J20	Vehicle	125	0.0090	**13888.9**
**31%**	**43%**	**26%**	**0%**		J20	Vehicle	208	0.0180	**11555.6**
**37%**	**43%**	**20%**	**0%**		J20	Vehicle	248	0.0180	**13777.8**
**39%**	**39%**	**22%**	**0%**		J20	Vehicle	131	0.0126	**10385.2**
**28%**	**63%**	**9%**	**0%**		J20	Vehicle	148	0.0126	**11732.9**
**58%**	**17%**	**18%**	**7%**		J20	VX-765	154	0.0218	**7056.5**
**38%**	**28%**	**25%**	**9%**		J20	VX-765	208	0.0218	**9530.8**
**30%**	**26%**	**34%**	**10%**		J20	VX-765	120	0.0119	**10080.6**
**36%**	**43%**	**16%**	**5%**		J20	VX-765	115	0.0126	**9116.8**
**60%**	**30%**	**7%**	**3%**		J20	VX-765	105	0.0126	**8324.0**

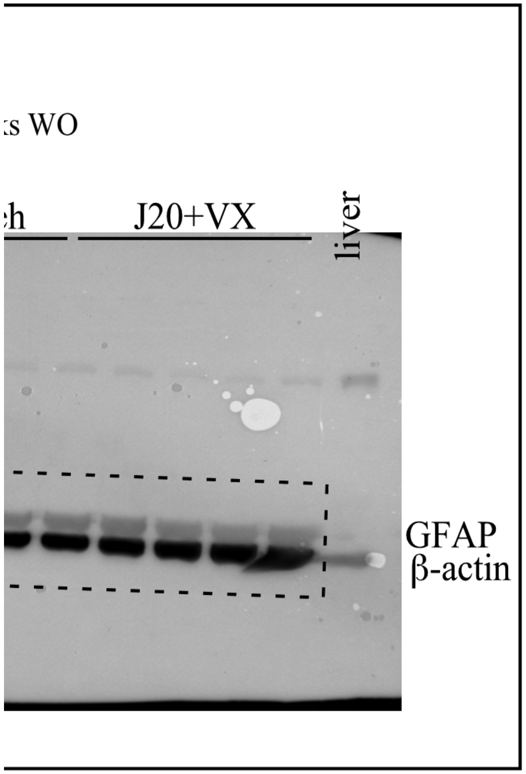

Figure 4d - GFAP western blot quantification (% of J20+veh)

Hippo	WT+veh	J20+veh	J20+VX
4-week WO	73.53	94.42	101.85
	52.66	98.71	99.72
	78.02	106.87	119.97
	74.44	133.15	118.86
	46.69	115.49	90.67
	12.51	51.57	42.02
	66.75	99.79	74.34
20-week WO	66.13	88.69	100.36
	58.97	104.46	87.38
	74.19	106.27	73.24
	88.10	100.58	77.93
	41.37	134.69	37.22
	75.02	70.62	103.76
	35.88	94.69	96.56
	110.46		

SUM/Total Stained Area (um2)	SUM/Total Area Analyzed (converted to mm^2)	**SUM Surface Area Staining (um^2 staining/mm^2)**
8378	1.457	**5748.4**
6410	1.457	**4398.1**
8426	1.457	**5781.3**
26862	2.355	**11408.1**
24445	2.355	**10381.6**
20304	2.355	**8622.9**
21974	2.355	**9332.2**
9934	1.457	**6816.0**
16205	1.457	**11118.7**
15021	1.457	**10306.3**
41306	2.355	**17542.3**
50632	2.355	**21503.0**
44340	2.355	**18830.8**
51560	2.355	**21897.1**
31076	2.355	**13197.7**
35587	2.355	**15113.5**
37958	2.355	**16120.4**
25211	2.355	**10706.9**
5168	1.457	**3545.9**
8586	1.457	**5891.1**
7423	1.457	**5093.1**
5272	1.457	**3617.3**
6710	1.457	**4603.9**
6896	1.457	**4731.5**
42645	2.355	**18111.0**
31408	2.355	**13338.7**
21313	2.355	**9051.4**
28560	2.355	**12129.2**

5900	1.457	**4048.2**
19678	1.457	**13501.6**
20250	1.457	**13894.1**
94965	2.355	**40330.8**
126300	2.355	**53638.5**
120926	2.355	**51356.2**
181133	2.355	**76925.6**
15180	1.457	**10415.4**
9364	1.457	**6424.9**
12194	1.457	**8366.6**
36805	2.355	**15630.8**
45712	2.355	**19413.5**
69627	2.355	**29570.0**
38838	2.355	**16494.2**
31373	2.355	**13323.8**
50370	2.355	**21391.7**
36211	2.355	**15378.5**
27045	2.355	**11485.8**
7093	1.457	**4866.7**
8927	1.457	**6125.1**
7902	1.457	**5421.8**
8882	1.457	**6094.2**
8056	1.457	**5527.4**
9176	1.457	**6295.9**
90518	2.355	**38442.2**
93752	2.355	**39815.7**
18517	1.457	**12705.0**
7023	1.457	**4818.7**
11458	1.457	**7861.7**
83416	2.355	**38850.3**
70680	2.355	**38897.1**

Iba-1 Distribution (#)				Iba-1 Distribution (%)			
i	ii	iii	iv	**i**	**ii**	**iii**	**iv**
138	52	0	0	**73%**	**27%**	**0%**	**0%**
84	24	0	0	**78%**	**22%**	**0%**	**0%**
111	38	0	0	**74%**	**26%**	**0%**	**0%**
160	28	0	0	**85%**	**15%**	**0%**	**0%**
148	24	0	0	**86%**	**14%**	**0%**	**0%**
132	34	0	0	**80%**	**20%**	**0%**	**0%**
140	14	0	0	**91%**	**9%**	**0%**	**0%**
28	100	5	0	**21%**	**75%**	**4%**	**0%**
104	120	6	0	**45%**	**52%**	**3%**	**0%**
80	160	8	0	**32%**	**65%**	**3%**	**0%**
242	36	0	0	**87%**	**13%**	**0%**	**0%**
204	66	2	0	**75%**	**24%**	**1%**	**0%**
184	74	2	0	**71%**	**28%**	**1%**	**0%**
174	44	0	0	**80%**	**20%**	**0%**	**0%**
66	26	12	4	**61%**	**24%**	**11%**	**4%**
98	40	0	0	**71%**	**29%**	**0%**	**0%**
98	84	0	0	**54%**	**46%**	**0%**	**0%**
150	50	4	0	**74%**	**25%**	**2%**	**0%**
176	34	4	0	**82%**	**16%**	**2%**	**0%**
144	50	0	0	**74%**	**26%**	**0%**	**0%**
144	22	2	0	**86%**	**13%**	**1%**	**0%**
95	30	1	1	**75%**	**24%**	**1%**	**1%**
102	42	0	3	**69%**	**29%**	**0%**	**2%**
136	71	1	0	**65%**	**34%**	**0%**	**0%**
64	9	0	0	**88%**	**12%**	**0%**	**0%**
68	11	1	0	**85%**	**14%**	**1%**	**0%**
55	24	1	0	**69%**	**30%**	**1%**	**0%**

71	23	0	0	**76%**	**24%**	**0%**	**0%**
90	40	4	0	**67%**	**30%**	**3%**	**0%**
89	58	2	0	**60%**	**39%**	**1%**	**0%**
77	43	1	0	**64%**	**36%**	**1%**	**0%**
74	48	6	0	**58%**	**38%**	**5%**	**0%**
118	106	6	0	**51%**	**46%**	**3%**	**0%**
86	148	2	0	**36%**	**63%**	**1%**	**0%**
80	116	10	0	**39%**	**56%**	**5%**	**0%**
113	46	1	0	**71%**	**29%**	**1%**	**0%**
104	50	0	0	**68%**	**32%**	**0%**	**0%**
158	78	4	0	**66%**	**33%**	**2%**	**0%**
70	30	1	0	**69%**	**30%**	**1%**	**0%**
75	24	1	0	**75%**	**24%**	**1%**	**0%**
65	44	1	0	**59%**	**40%**	**1%**	**0%**
66	29	0	0	**69%**	**31%**	**0%**	**0%**
126	28	2	0	**81%**	**18%**	**1%**	**0%**
106	34	2	0	**75%**	**24%**	**1%**	**0%**
100	20	0	0	**83%**	**17%**	**0%**	**0%**
68	31	0	0	**69%**	**31%**	**0%**	**0%**
72	24	1	0	**74%**	**25%**	**1%**	**0%**
62	33	0	0	**65%**	**35%**	**0%**	**0%**
59	39	0	0	**60%**	**40%**	**0%**	**0%**
63	52	10	0	**50%**	**42%**	**8%**	**0%**
106	92	10	0	**51%**	**44%**	**5%**	**0%**
148	90	10	0	**60%**	**36%**	**4%**	**0%**
62	62	7	0	**47%**	**47%**	**5%**	**0%**
48	95	5	0	**32%**	**64%**	**3%**	**0%**
96	47	8	3	**62%**	**31%**	**5%**	**2%**
126	66	12	4	**61%**	**32%**	**6%**	**2%**
83	28	9	0	**69%**	**23%**	**8%**	**0%**
59	50	5	1	**51%**	**43%**	**4%**	**1%**
70	32	3	0	**67%**	**30%**	**3%**	**0%**

Figure 5a - IL-1b & IL-18 qPCR

					IL-1β hippo
Genotype	Treatment	WO (Weeks)	Mean Ct IL-1β	Mean Ct HPRT+18s	ΔCt
WT	Vehicle	4	29.0347023	15.09490585	13.93979645
WT	Vehicle	4	26.85299301	15.37163448	11.48135853
WT	Vehicle	4	28.06803131	15.34946823	12.71856308
WT	Vehicle	4	28.48034096	15.17251396	13.307827
J20	Vehicle	4	28.45514297	15.37090969	13.08423328
J20	Vehicle	4	28.44120216	15.28404999	13.15715218
J20	Vehicle	4	26.37270737	14.81592607	11.55678129
J20	VX-765	4	28.29172707	15.5962553	12.69547176
J20	VX-765	4	28.83581543	15.2533884	13.58242702
J20	VX-765	4	27.63921356	15.36834908	12.27086449
WT	Vehicle	12	29.46294785	15.26136637	14.20158148
WT	Vehicle	12	27.69842529	14.97976685	12.71865845
WT	Vehicle	12	28.46947098	15.04685688	13.4226141
WT	Vehicle	12	26.84332848	15.26461792	11.57871056
J20	Vehicle	12	27.38851547	15.21114445	12.17737103
J20	Vehicle	12	29.20269012	15.20016241	14.00252771
J20	Vehicle	12	26.51593018	14.65073061	11.86519957
J20	VX-765	12	27.833992	15.46437502	12.36961699
J20	VX-765	12	27.04841614	15.16145897	11.88695717
J20	VX-765	12	28.81360626	15.32771206	13.4858942
WT	Vehicle	20	27.4281311	15.10013771	12.32799339
WT	Vehicle	20	28.50073242	15.26441288	13.23631954
WT	Vehicle	20	28.51007271	15.46088028	13.04919243
WT	Vehicle	20	29.79371643	15.31291771	14.48079872
J20	Vehicle	20	27.53962898	15.21928406	12.32034492
J20	Vehicle	20	28.37399864	15.23942089	13.13457775
J20	Vehicle	20	28.09940338	15.38573456	12.71366882
J20	Vehicle	20	26.64273071	15.03895283	11.60377789

J20	VX-765	20	27.51235771	15.04809523	12.46426249
J20	VX-765	20	28.52693939	15.19388771	13.33305168
J20	VX-765	20	26.47532654	15.09176922	11.38355732
J20	VX-765	20	26.41855621	15.5668292	10.85172701

Figure 5b - IL-1β western blot in the hippocampus

Antibody: IL-1β, Abcam, ab9722, lot GR308444-1 (1:1000)

IL-1β at 4-week WO

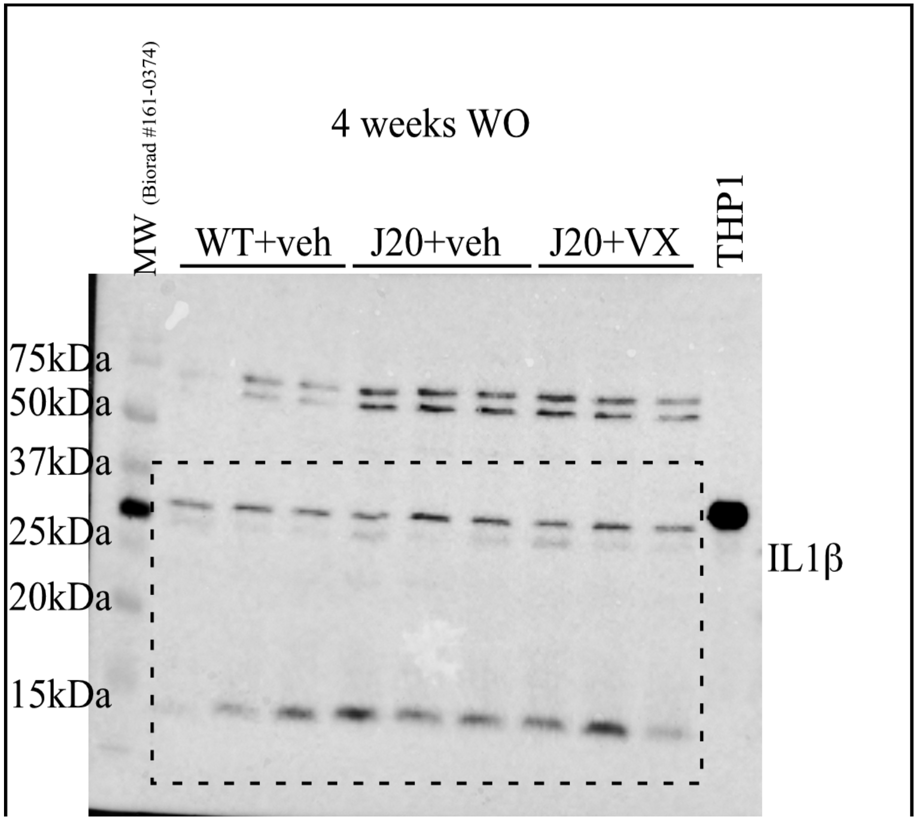

IL-1β at 20-week WO

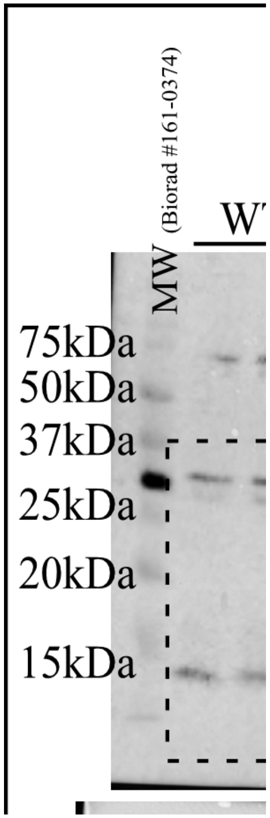

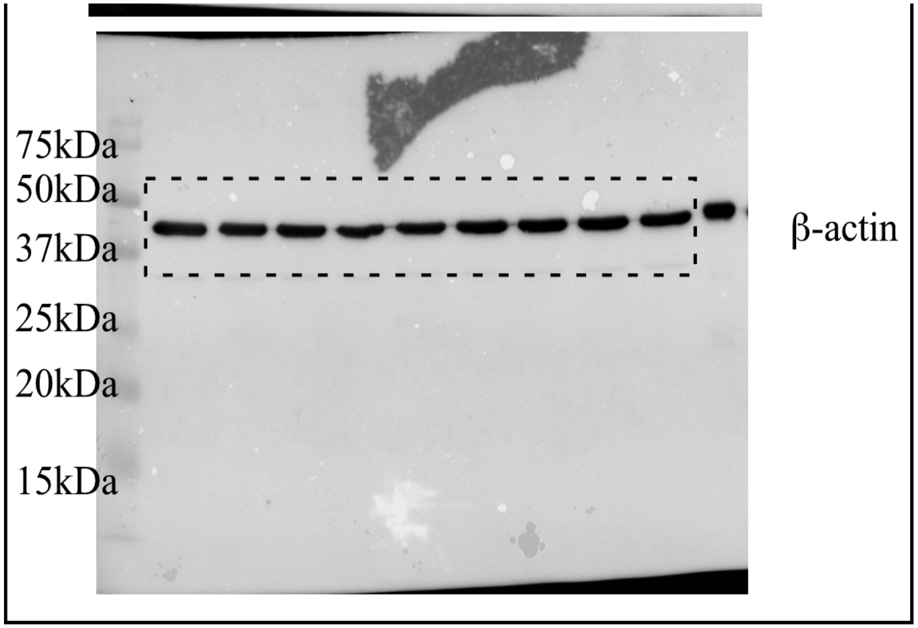

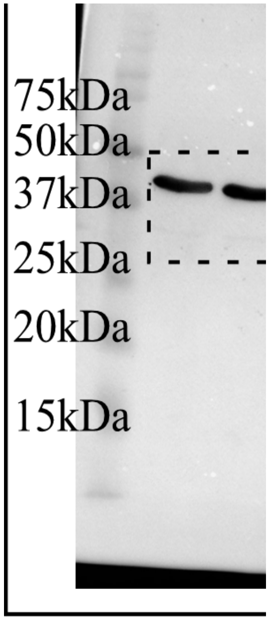

*Dotted area is what is shown in publication figure

Figure 5i IL-1β ELISA

			Hippo		
		Washout (weeks)	Calc. Concentration	Sample Volume (ml)	Total Amount (pg)
WT	Vehicle	4	0.00	0.03	0.0000
WT	Vehicle	4	0.00	0.03	0.0000

WT	Vehicle	4	0.00	0.03	0.0000
WT	Vehicle	4	0.40	0.03	0.0101
WT	Vehicle	4	0.32	0.03	0.0081
WT	Vehicle	4	0.44	0.03	0.0111
WT	Vehicle	4	0.44	0.03	0.0111
J20	Vehicle	4	0.11	0.03	0.0027
J20	Vehicle	4	0.18	0.03	0.0045
J20	Vehicle	4	0.07	0.03	0.0018
J20	Vehicle	4	0.44	0.03	0.0109
J20	Vehicle	4	0.40	0.03	0.0100
J20	Vehicle	4	0.39	0.03	0.0096
J20	Vehicle	4	0.38	0.03	0.0095
J20	VX-765	4	0.01	0.03	0.0002
J20	VX-765	4	0.02	0.03	0.0004
J20	VX-765	4	0.01	0.03	0.0003
J20	VX-765	4	0.46	0.03	0.0115
J20	VX-765	4	0.35	0.03	0.0087
J20	VX-765	4	0.39	0.03	0.0098
J20	VX-765	4	0.40	0.03	0.0101
J20	VX-765	4	0.43	0.03	0.0107
WT	Vehicle	12	0.04	0.03	0.00108
WT	Vehicle	12	0.12	0.03	0.00302
WT	Vehicle	12	0.05	0.03	0.00130
WT	Vehicle	12	0.00	0.03	0.00000
WT	Vehicle	12	0.32	0.03	0.00810
WT	Vehicle	12	0.45	0.03	0.01113
WT	Vehicle	12	0.33	0.03	0.00834
WT	Vehicle	12	0.47	0.03	0.01185
J20	Vehicle	12	0.12	0.03	0.00289
J20	Vehicle	12	0.05	0.03	0.00130
J20	Vehicle	12	0.09	0.03	0.00235
J20	Vehicle	12	0.59	0.03	0.01484

J20	Vehicle	12	0.43	0.03	0.01071
J20	Vehicle	12	1.54	0.03	0.03844
J20	VX-765	12	0.09	0.03	0.00221
J20	VX-765	12	0.03	0.03	0.00086
J20	VX-765	12	0.04	0.03	0.00104
J20	VX-765	12	0.47	0.03	0.01167
J20	VX-765	12	0.47	0.03	0.01183
J20	VX-765	12	0.89	0.03	0.02219
J20	VX-765	12	0.53	0.03	0.01319
WT	Vehicle	20	0.06	0.03	0.00145
WT	Vehicle	20	0.00	0.03	0.00000
WT	Vehicle	20	0.10	0.03	0.00244
WT	Vehicle	20	0.05	0.03	0.00120
WT	Vehicle	20	0.43	0.03	0.01065
WT	Vehicle	20	0.36	0.03	0.00904
WT	Vehicle	20	0.37	0.03	0.00934
WT	Vehicle	20	0.53	0.03	0.01325
J20	Vehicle	20	0.35	0.03	0.00865
J20	Vehicle	20	0.10	0.03	0.00245
J20	Vehicle	20	0.12	0.03	0.00301
J20	Vehicle	20	0.08	0.03	0.00209
J20	Vehicle	20	1.07	0.03	0.02676
J20	Vehicle	20	0.77	0.03	0.01927
J20	Vehicle	20	0.85	0.03	0.02136
J20	VX-765	20	0.00	0.03	0.00000
J20	VX-765	20	0.35	0.03	0.00865
J20	VX-765	20	0.10	0.03	0.00255
J20	VX-765	20	0.10	0.03	0.00244
J20	VX-765	20	0.60	0.03	0.01509
J20	VX-765	20	0.54	0.03	0.01355
J20	VX-765	20	0.54	0.03	0.01361

Figure 5j - Casp1 and Casp6 qPCR

				**Casp1 hippo**	
Genotype	Treatment	WO (Weeks)	Mean Ct Casp1	Mean Ct HPRT+18s	ΔCt
WT	Vehicle	4	24.94897842	15.09490585	9.854072571
WT	Vehicle	4	22.30825615	15.37163448	6.936621666
WT	Vehicle	4	23.30211639	15.34946823	7.952648163
WT	Vehicle	4	25.13892365	15.17251396	9.966409683
J20	Vehicle	4	25.84470749	15.37090969	10.4737978
J20	Vehicle	4	25.40044022	15.28404999	10.11639023
J20	Vehicle	4	24.12657166	14.81592607	9.31064558
J20	VX-765	4	25.04060173	15.5962553	9.444346428
J20	VX-765	4	25.3991394	15.2533884	10.145751
J20	VX-765	4	25.90226936	15.36834908	10.53392029
WT	Vehicle	12	25.49831009	15.26136637	10.23694372
WT	Vehicle	12	23.0785675	14.97976685	8.098800659
WT	Vehicle	12	25.11908722	15.04685688	10.07223034
WT	Vehicle	12	22.29694748	15.26461792	7.032329559
J20	Vehicle	12	24.1714325	15.21114445	8.960288048
J20	Vehicle	12	25.07314682	15.20016241	9.872984409
J20	Vehicle	12	23.88545609	14.65073061	9.234725475
J20	VX-765	12	25.79445648	15.46437502	10.33008146
J20	VX-765	12	24.36670303	15.16145897	9.205244064
J20	VX-765	12	24.88580322	15.32771206	9.558091164
WT	Vehicle	20	23.92237091	15.10013771	8.8222332
WT	Vehicle	20	24.05766296	15.26441288	8.793250084
WT	Vehicle	20	25.85461426	15.46088028	10.39373398
WT	Vehicle	20	24.59355545	15.31291771	9.280637741
J20	Vehicle	20	24.89929581	15.21928406	9.680011749
J20	Vehicle	20	25.04869843	15.23942089	9.809277534
J20	Vehicle	20	26.07449913	15.38573456	10.68876457
J20	Vehicle	20	24.00456619	15.03895283	8.965613365

J20	VX-765	20	24.4878273	15.04809523	9.439732075
J20	VX-765	20	25.30406189	15.19388771	10.11017418
J20	VX-765	20	23.43918037	15.09176922	8.347411156
J20	VX-765	20	24.31295395	15.5668292	8.746124744

$2^{-\Delta Ct}$	Genotype	Treatment	WO (Weeks)	Mean Ct IL-18
**6.36360E-05**	WT	Vehicle	4	20.57417679
**3.49757E-04**	WT	Vehicle	4	19.9042778
**1.48365E-04**	WT	Vehicle	4	20.17718887
**9.86154E-05**	WT	Vehicle	4	20.08216476
**1.15147E-04**	J20	Vehicle	4	20.42238617
**1.09472E-04**	J20	Vehicle	4	20.36158943
**3.31942E-04**	J20	Vehicle	4	19.0434227
**1.50759E-04**	J20	VX-765	4	20.26403809
**8.15234E-05**	J20	VX-765	4	19.84205246
**2.02349E-04**	J20	VX-765	4	20.05990982
**5.30760E-05**	WT	Vehicle	12	20.20211601
**1.48355E-04**	WT	Vehicle	12	20.7601738
**9.10732E-05**	WT	Vehicle	12	20.01478004
**3.26935E-04**	WT	Vehicle	12	20.03585243
**2.15897E-04**	J20	Vehicle	12	20.31573486
**6.09283E-05**	J20	Vehicle	12	20.07816887
**2.68052E-04**	J20	Vehicle	12	18.92856979
**1.88962E-04**	J20	VX-765	12	20.34121704
**2.64040E-04**	J20	VX-765	12	20.29390717
**8.71648E-05**	J20	VX-765	12	20.14831161
**1.94493E-04**	WT	Vehicle	20	19.9550724
**1.03626E-04**	WT	Vehicle	20	20.46099472
**1.17978E-04**	WT	Vehicle	20	20.21567535
**4.37366E-05**	WT	Vehicle	20	20.38136673
**1.95527E-04**	J20	Vehicle	20	20.2617836
**1.11198E-04**	J20	Vehicle	20	20.03141975
**1.48869E-04**	J20	Vehicle	20	20.64006805
**3.21303E-04**	J20	Vehicle	20	18.97956085

**1.76963E-04**	J20	VX-765	20	19.97141266
**9.69062E-05**	J20	VX-765	20	20.46414948
**3.74290E-04**	J20	VX-765	20	19.79588699
**5.41134E-04**	J20	VX-765	20	19.23885536

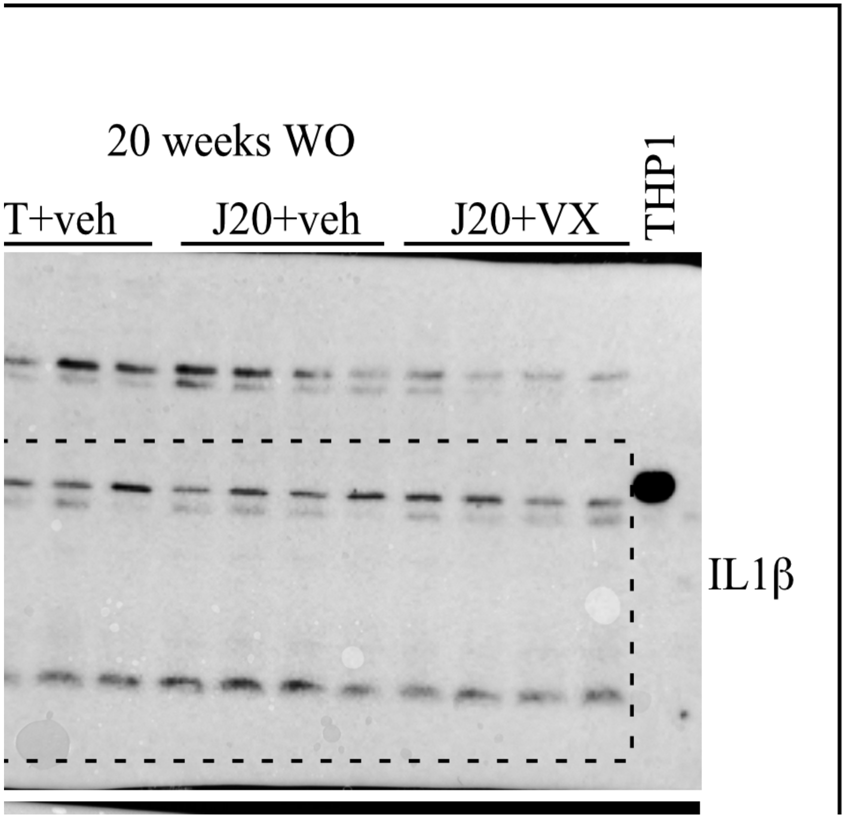

Figure 4c-h - IL-1β western blot qua

4-week WO Hippo	WT+veh
pro IL-1β	**99.9**
	**114.8**
	**85.3**
	**78.1**
	**69.1**
	**115.0**
	**137.8**
mature IL-1β	**3.0**
	**70.1**
	**226.9**
	**60.8**
	**88.1**
	**126.5**
	**124.6**
Total IL-1β	**50.3**
	**91.9**
	**157.8**
	**66.4**
	**81.9**
	**122.8**
	**128.9**

20-week WO Hippo	WT+veh

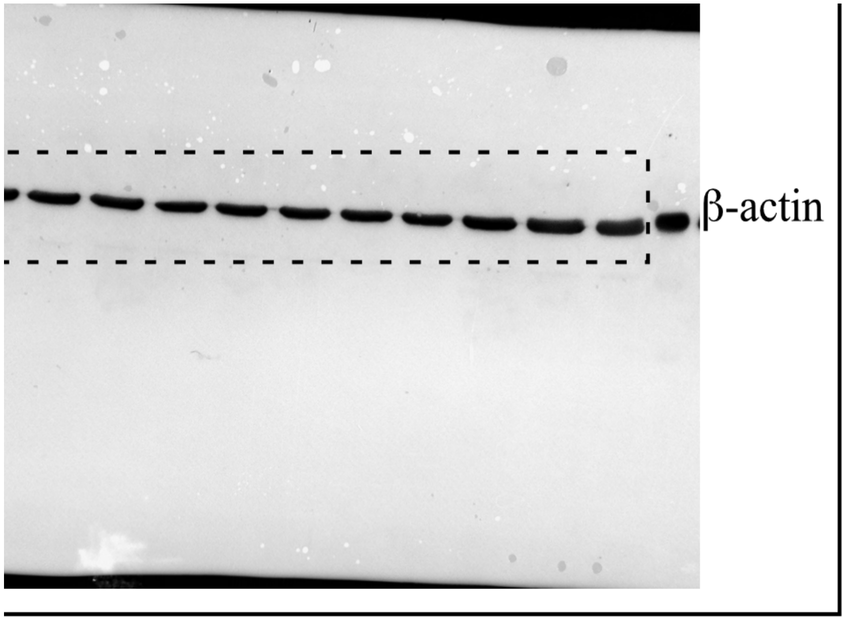

pro IL-1β	**52.5**
	**91.9**
	**122.9**
	**132.7**
	**125.6**
	**92.3**
	**25.0**
	**157.1**
mature IL-1β	**66.5**
	**48.2**
	**139.5**
	**145.9**
	**85.1**
	**103.4**
	**98.4**
	**113.1**
Total IL-1β	**60.7**
	**66.2**
	**132.7**
	**140.4**
	**95.5**
	**100.5**
	**79.4**
	**124.5**

Cortex

Protein Loaded (μg)	**Final [conc] pg/mg**	Calc. Concentration	Sample Volume (ml)	Total Amount (pg)
100	**0.0000**	0.28	0.03	0.0070
100	**0.0000**	0.37	0.03	0.0093

100	**0.0000**	0.33	0.03	0.0082
125	**0.0809**	0.74	0.03	0.0185
125	**0.0648**	0.75	0.03	0.0188
125	**0.0885**	0.75	0.03	0.0188
125	**0.0885**	0.67	0.03	0.0166
100	**0.0266**	0.34	0.03	0.0086
100	**0.0452**	0.28	0.03	0.0071
100	**0.0176**	0.34	0.03	0.0085
125	**0.0876**	0.51	0.03	0.0127
125	**0.0799**	0.57	0.03	0.0142
125	**0.0771**	0.62	0.03	0.0156
125	**0.0761**	0.48	0.03	0.0120
100	**0.0021**	0.20	0.03	0.0050
100	**0.0042**	0.24	0.03	0.0060
100	**0.0029**	0.19	0.03	0.0048
125	**0.0919**	0.61	0.03	0.0152
125	**0.0700**	0.47	0.03	0.0118
125	**0.0780**	0.59	0.03	0.0148
125	**0.0809**	0.68	0.03	0.0169
125	**0.0857**	0.48	0.03	0.0121
100	**0.01080**	0.37	0.03	0.009
100	**0.03018**	0.49	0.03	0.012
100	**0.01303**	0.52	0.03	0.013
100	**0.00000**	0.36	0.03	0.009
125	**0.06482**	0.69	0.03	0.017
125	**0.08904**	0.69	0.03	0.017
125	**0.06669**	0.83	0.03	0.021
125	**0.09480**	0.70	0.03	0.017
100	**0.02894**	0.29	0.03	0.007
100	**0.01303**	0.22	0.03	0.006
100	**0.02348**	0.24	0.03	0.006
125	**0.11874**	0.79	0.03	0.020

125	**0.08566**	0.70	0.03	0.018
125	**0.30755**	1.03	0.03	0.026
100	**0.02207**	0.24	0.03	0.006
100	**0.00858**	0.27	0.03	0.007
100	**0.01036**	0.23	0.03	0.006
125	**0.09335**	0.69	0.03	0.017
125	**0.09462**	0.97	0.03	0.024
125	**0.17752**	0.78	0.03	0.019
125	**0.10549**	0.82	0.03	0.020
100	**0.01447**	0.31	0.03	0.008
100	**0.00000**	0.30	0.03	0.008
100	**0.02435**	0.33	0.03	0.008
100	**0.01200**	0.41	0.03	0.010
125	**0.08518**	0.83	0.03	0.021
125	**0.07234**	0.63	0.03	0.016
125	**0.07470**	0.71	0.03	0.018
125	**0.10598**	0.82	0.03	0.020
100	**0.08651**	0.36	0.03	0.009
100	**0.02449**	0.21	0.03	0.005
100	**0.03009**	0.35	0.03	0.009
100	**0.02093**	0.27	0.03	0.007
125	**0.21409**	0.79	0.03	0.020
125	**0.15416**	1.06	0.03	0.027
125	**0.17089**	0.84	0.03	0.021
100	**0.00000**	0.23	0.03	0.006
100	**0.08649**	0.36	0.03	0.009
100	**0.02554**	0.35	0.03	0.009
100	**0.02435**	0.31	0.03	0.008
125	**0.12071**	0.68	0.03	0.017
125	**0.10842**	0.74	0.03	0.019
125	**0.10891**	0.63	0.03	0.016

$2^{-\Delta Ct}$	Genotype	Treatment	WO (Weeks)	Mean Ct Casp6
**0.00108051**	WT	Vehicle	4	23.09135246
**0.00816336**	WT	Vehicle	4	23.69684601
**0.00403659**	WT	Vehicle	4	23.84801102
**0.00099957**	WT	Vehicle	4	23.76126099
**0.00070319**	J20	Vehicle	4	23.9985199
**0.00090087**	J20	Vehicle	4	23.41037178
**0.00157477**	J20	Vehicle	4	22.50354385
**0.00143539**	J20	VX-765	4	23.77772713
**0.00088272**	J20	VX-765	4	23.05110359
**0.00067449**	J20	VX-765	4	23.68867111
**0.00082865**	WT	Vehicle	12	23.7630043
**0.00364769**	WT	Vehicle	12	23.49138451
**0.00092887**	WT	Vehicle	12	23.71401787
**0.00763938**	WT	Vehicle	12	23.91001129
**0.00200763**	J20	Vehicle	12	23.93011093
**0.00106644**	J20	Vehicle	12	23.90492249
**0.00165986**	J20	Vehicle	12	22.45653534
**0.00077685**	J20	VX-765	12	24.22476768
**0.00169412**	J20	VX-765	12	23.68422699
**0.00132656**	J20	VX-765	12	22.91827011
**0.00220924**	WT	Vehicle	20	23.45905495
**0.00225407**	WT	Vehicle	20	23.5504303
**0.00074332**	WT	Vehicle	20	24.31675339
**0.00160787**	WT	Vehicle	20	23.37968063
**0.00121906**	J20	Vehicle	20	23.81767273
**0.00111459**	J20	Vehicle	20	23.61899757
**0.00060584**	J20	Vehicle	20	23.98420715
**0.00200024**	J20	Vehicle	20	22.80439377

**0.00143998**	J20	VX-765	20	23.13829041
**0.00090476**	J20	VX-765	20	23.87419701
**0.00307029**	J20	VX-765	20	23.04981995
**0.00232892**	J20	VX-765	20	23.57238388

IL-18 hippo

Mean Ct HPRT+18s	ΔCt	$2^{-\Delta Ct}$
15.09490585	5.479270935	**0.022417**
15.37163448	4.532643318	**0.043205**
15.34946823	4.827720642	**0.035214**
15.17251396	4.909650803	**0.033270**
15.37090969	5.051476479	**0.030155**
15.28404999	5.077539444	**0.029615**
14.81592607	4.227496624	**0.053382**
15.5962553	4.667782784	**0.039342**
15.2533884	4.588664055	**0.041560**
15.36834908	4.691560745	**0.038699**
15.26136637	4.940749645	**0.032560**
14.97976685	5.780406952	**0.018194**
15.04685688	4.967923164	**0.031953**
15.26461792	4.771234512	**0.036620**
15.21114445	5.104590416	**0.029065**
15.20016241	4.878006458	**0.034007**
14.65073061	4.277839184	**0.051552**
15.46437502	4.876842022	**0.034035**
15.16145897	5.132448196	**0.028509**
15.32771206	4.820599556	**0.035388**
15.10013771	4.854934692	**0.034556**
15.26441288	5.196581841	**0.027269**
15.46088028	4.754795074	**0.037039**
15.31291771	5.06844902	**0.029802**
15.21928406	5.042499542	**0.030343**
15.23942089	4.791998863	**0.036096**
15.38573456	5.254333496	**0.026199**
15.03895283	3.940608025	**0.065127**

15.04809523	4.923317432	**0.032956**
15.19388771	5.270261765	**0.025912**
15.09176922	4.704117775	**0.038364**
15.5668292	3.672026157	**0.078453**

ntification (% of WT+veh)

J20+veh	J20+VX	4-week WO Cortex	WT+veh	J20+veh	J20+VX
**50.6**	**71.7**	pro IL-1β	**78.9**	**69.9**	**73.8**
**110.7**	**90.7**		**103.7**	**83.6**	**84.6**
**104.8**	**55.0**		**114.0**	**96.0**	**78.1**
**114.1**	**101.0**		**103.4**	**90.9**	**83.1**
**79.7**	**71.0**				
**135.0**	**60.4**				
**30.8**	**60.4**				
**294.4**	**151.8**	mature IL-1β	**93.6**	**86.8**	**25.5**
**165.1**	**225.7**		**98.3**	**85.6**	**91.0**
**160.6**	**41.4**		**104.0**	**111.0**	**113.0**
**143.0**	**133.2**		**104.0**	**103.6**	**79.1**
**127.0**	**134.7**				
**130.8**	**118.4**				
**130.5**	**102.3**				
**175.4**	**112.6**	Total IL-1β	**87.7**	**80.1**	**44.8**
**138.5**	**159.8**		**100.5**	**84.8**	**88.4**
**133.4**	**48.0**		**108.0**	**105.0**	**99.1**
**133.6**	**122.7**		**103.8**	**98.6**	**80.7**
**111.6**	**114.0**				
**132.2**	**99.6**				
**98.2**	**88.7**				
J20+veh	J20+VX	20-week WO Cortex	WT+veh	J20+veh	J20+VX

**55.8**	**109.8**	pro IL-1β	**120.5**	**95.2**	**87.8**
**108.0**	**91.1**		**92.7**	**91.2**	**69.1**
**91.0**	**51.0**		**89.1**	**95.6**	**55.2**
**123.0**	**47.3**		**97.8**		
**161.0**	**232.7**				
**247.9**	**195.0**				
**148.7**	**143.6**				
**163.5**	**64.0**	mature IL-1β	**91.3**	**137.7**	**98.1**
**169.2**	**70.0**		**107.7**	**115.0**	**92.2**
**149.7**	**36.4**		**96.2**	**124.4**	**98.8**
**79.7**	**57.7**		**104.8**		
**113.0**	**132.1**				
**126.4**	**113.7**				
**93.4**	**114.2**				
**119.1**	**82.9**	Total IL-1β	**102.4**	**121.6**	**94.2**
**143.9**	**78.7**		**102.0**	**105.9**	**83.4**
**125.5**	**42.4**		**93.5**	**113.5**	**82.2**
**97.6**	**53.4**		**102.2**		
**125.5**	**158.1**				
**157.8**	**134.7**				
**107.7**	**121.8**				

Protein Loaded (μg)	**Final [conc] pg/mg**
100	**0.0701**
100	**0.0927**

100	**0.0819**
125	**0.1477**
125	**0.1508**
125	**0.1502**
125	**0.1331**
100	**0.0862**
100	**0.0711**
100	**0.0851**
125	**0.1020**
125	**0.1139**
125	**0.1247**
125	**0.0961**
100	**0.0498**
100	**0.0604**
100	**0.0477**
125	**0.1217**
125	**0.0943**
125	**0.1187**
125	**0.1356**
125	**0.0967**
100	**0.0927**
100	**0.1230**
100	**0.1296**
100	**0.0905**
125	**0.1386**
125	**0.1386**
125	**0.1655**
125	**0.1398**
100	**0.0733**
100	**0.0562**
100	**0.0597**
125	**0.1587**

125	**0.1404**
125	**0.2057**
100	**0.0604**
100	**0.0669**
100	**0.0587**
125	**0.1386**
125	**0.1945**
125	**0.1550**
125	**0.1636**
100	**0.0776**
100	**0.0754**
100	**0.0830**
100	**0.1035**
125	**0.1654**
125	**0.1253**
125	**0.1422**
125	**0.1636**
100	**0.0894**
100	**0.0530**
100	**0.0883**
100	**0.0669**
125	**0.1587**
125	**0.2125**
125	**0.1685**
100	**0.0583**
100	**0.0894**
100	**0.0883**
100	**0.0787**
125	**0.1362**
125	**0.1489**
125	**0.1253**

Casp6 hippo

Mean Ct HPRT+18s	ΔCt	**$2^{-\Delta Ct}$**
15.09490585	7.996446609	**0.00391588**
15.37163448	8.325211525	**0.00311790**
15.34946823	8.498542786	**0.00276493**
15.17251396	8.588747025	**0.00259734**
15.37090969	8.627610207	**0.00252831**
15.28404999	8.126321793	**0.00357877**
14.81592607	7.687617779	**0.00485061**
15.5962553	8.181471825	**0.00344454**
15.2533884	7.797715187	**0.00449421**
15.36834908	8.320322037	**0.00312848**
15.26136637	8.501637936	**0.00275900**
14.97976685	8.511617661	**0.00273998**
15.04685688	8.667160988	**0.00245994**
15.26461792	8.645393372	**0.00249734**
15.21114445	8.718966484	**0.00237317**
15.20016241	8.704760075	**0.00239666**
14.65073061	7.805804729	**0.00446909**
15.46437502	8.760392666	**0.00230600**
15.16145897	8.522768021	**0.00271889**
15.32771206	7.590558052	**0.00518817**
15.10013771	8.358917236	**0.00304590**
15.26441288	8.286017418	**0.00320376**
15.46088028	8.855873108	**0.00215832**
15.31291771	8.066762924	**0.00372960**
15.21928406	8.598388672	**0.00258004**
15.23942089	8.379576683	**0.00300259**
15.38573456	8.598472595	**0.00257989**
15.03895283	7.765440941	**0.00459589**

15.5668292	7.571461201	**0.00525730**
15.04809523	8.82610178	**0.00220333**
15.19388771	7.855932236	**0.00431647**
15.09176922	8.480614662	**0.00279950**

Figure 6b - Aβ staining density

Hippo	Genotype	Treatment	SUM/Total Stained Area (um2)	SUM/Total Area Analyzed (converted to mm^2)	**SUM Surface Area Staining (um^2 staining/mm^2)**
4-week WO	J20	Vehicle	12184	2.5561	**4766.61**
	J20	Vehicle	7692	2.5561	**3009.26**
	J20	Vehicle	5098	1.9171	**2659.25**
	J20	Vehicle	7856.9	2.3547	**3336.76**
	J20	Vehicle	14879.1	2.3547	**6319.03**
	J20	Vehicle	1897.9	2.3547	**806.02**
	J20	Vehicle	3317.4	2.3547	**1408.87**
	J20	VX-765	372.1	2.3547	**158.03**
	J20	VX-765	3306.9	2.3547	**1404.41**
	J20	VX-765	2741.1	2.3547	**1164.12**
	J20	VX-765	4020.3	2.3547	**1707.39**
	J20	VX-765	5426	2.5561	**2122.76**
	J20	VX-765	3692	1.2781	**2888.76**
	J20	VX-765	2225	1.2781	**1740.93**
12-week WO	J20	Vehicle	8694	1.2781	**6802.52**
	J20	Vehicle	4486	1.2781	**3510.02**
	J20	Vehicle	3202	0.6390	**5010.74**
	J20	Vehicle	34975.1	2.3547	**14853.63**
	J20	Vehicle	30214.9	2.3547	**12832.01**
	J20	Vehicle	22134.9	2.3547	**9400.50**
	J20	Vehicle	16945.6	2.3547	**7196.65**
	J20	VX-765	3925	0.6390	**6142.14**
	J20	VX-765	9032	1.2781	**7066.98**
	J20	VX-765	2663	0.6390	**4167.27**
	J20	VX-765	30798.5	2.3547	**13079.86**
	J20	VX-765	12120.4	2.3547	**5147.43**
	J20	VX-765	40378.6	2.3547	**17148.45**
	J20	VX-765	15787.4	2.3547	**6704.77**

20-week WO	J20	Vehicle	103605	5.1122	**20266.13**
	J20	Vehicle	153057	5.1122	**29939.42**
	J20	Vehicle	37456	2.5561	**14653.51**
	J20	Vehicle	85599	2.5561	**33487.97**
	J20	Vehicle	145508.5	2.3547	**61796.21**
	J20	Vehicle	57379.2	2.3547	**24368.45**
	J20	VX-765	87233.2	2.3547	**37047.19**
	J20	VX-765	83609	2.3547	**35508.03**
	J20	VX-765	54904	5.1122	**10739.75**
	J20	VX-765	101242	5.1122	**19803.91**
	J20	VX-765	112475	5.1122	**22001.19**
	J20	VX-765	129362	5.1122	**25304.45**

Figure 6c - Aβ ELISA (RIPA)

See source data for Supplemental Figure 8 for additional data

			RIPA		
**Hippo**			**Total Aβ (pg/mg)**	**Aβ42/Total Aβ**	**Cortex**
4-week WO	J20	Vehicle	**386.97**	**0.14**	4-week WO
	J20	Vehicle	**357.78**	**0.13**	
	J20	Vehicle	**461.73**	**0.10**	
	J20	Vehicle	**359.18**	**0.12**	
	J20	Vehicle	**319.53**	**0.10**	
	J20	Vehicle	**321.48**	**0.10**	
	J20	Vehicle	**323.86**	**0.10**	
	J20	VX-765	**417.93**	**0.10**	
	J20	VX-765	**393.96**	**0.11**	
	J20	VX-765	**362.13**	**0.11**	
	J20	VX-765	**245.49**	**0.14**	
	J20	VX-765	**356.15**	**0.12**	
	J20	VX-765	**325.12**	**0.10**	
	J20	VX-765	**322.59**	**0.09**	
	J20	VX-765	**307.16**	**0.10**	

12-week WO	J20	Vehicle	**448.15**	**0.13**	12-week WO
	J20	Vehicle	**475.80**	**0.15**	
	J20	Vehicle	**470.93**	**0.13**	
	J20	Vehicle	**292.55**	**0.13**	
	J20	Vehicle	**303.23**	**0.13**	
	J20	Vehicle	**296.48**	**0.13**	
	J20	VX-765	**511.43**	**0.13**	
	J20	VX-765	**547.76**	**0.12**	
	J20	VX-765	**294.12**	**0.16**	
	J20	VX-765	**263.94**	**0.14**	
	J20	VX-765	**351.59**	**0.09**	
	J20	VX-765	**353.96**	**0.09**	
	J20	VX-765	**352.69**	**0.09**	
20-week WO	J20	Vehicle	**226.50**	**0.19**	20-week WO
	J20	Vehicle	**292.01**	**0.20**	
	J20	Vehicle	**298.00**	**0.14**	
	J20	Vehicle	**330.83**	**0.18**	
	J20	Vehicle	**640.53**	**0.28**	
	J20	Vehicle	**538.46**	**0.36**	
	J20	Vehicle	**540.57**	**0.19**	
	J20	VX-765	**352.76**	**0.14**	
	J20	VX-765	**343.84**	**0.14**	
	J20	VX-765	**354.58**	**0.12**	
	J20	VX-765	**350.22**	**0.13**	
	J20	VX-765	**456.12**	**0.22**	
	J20	VX-765	**463.31**	**0.21**	
	J20	VX-765	**410.09**	**0.17**	

Figure 6e - IDE western blot quantification (% of J20+veh)

Hippo	WT+veh	J20+veh	J20+VX
4-week WO	**69.4**	**113.0**	**57.4**
	**142.4**	**84.9**	**68.2**
	**69.0**	**102.1**	**63.5**

Figure 6f - Nep we

Hippo

4-week WO

	48.9	78.8	81.8	
	61.2	99.7	94.3	
	81.4	127.3	95.7	
	81.5	94.2	91.2	
20-week WO	83.2	70.3	111.9	20-week WO
	55.9	128.6	73.8	
	78.5	88.8	69.0	
	118.7	112.4	109.8	
	108.0	100.9	150.0	
	140.3	142.9	114.9	
	114.3	56.2	140.3	
	130.4			

Cortex	Genotype	Treatment	SUM/Total Stained Area (um2)	SUM/Total Area Analyzed (converted to mm^2)	**SUM Surface Area Staining (um^2 staining/mm^2)**
4-week WO	J20	Vehicle	1305	2.5561	**510.54**
	J20	Vehicle	1328	5.1122	**259.77**
	J20	Vehicle	916	2.5561	**358.36**
	J20	Vehicle	7856.9	2.3547	**3336.76**
	J20	Vehicle	14879.1	2.3547	**6319.03**
	J20	Vehicle	1897.9	2.3547	**806.02**
	J20	Vehicle	3317.4	2.3547	**1408.87**
	J20	VX-765	838	1.9171	**437.12**
	J20	VX-765	350	1.2781	**273.85**
	J20	VX-765	1348	1.9171	**703.15**
	J20	VX-765	372.1	2.3547	**158.03**
	J20	VX-765	3306.9	2.3547	**1404.41**
	J20	VX-765	2741.1	2.3547	**1164.12**
	J20	VX-765	4020.3	2.3547	**1707.39**
12-week WO	J20	Vehicle	3113	1.2781	**2435.73**
	J20	Vehicle	2636	0.6390	**4125.01**
	J20	Vehicle	1421	0.6390	**2223.69**
	J20	Vehicle	34975.1	2.3547	**14853.63**
	J20	Vehicle	30214.9	2.3547	**12832.01**
	J20	Vehicle	22134.9	2.3547	**9400.50**
	J20	Vehicle	16945.6	2.3547	**7196.65**
	J20	VX-765	2713	1.2781	**2122.76**
	J20	VX-765	847	0.6390	**1325.45**
	J20	VX-765	938	0.6390	**1467.85**
	J20	VX-765	30798.5	2.3547	**13079.86**
	J20	VX-765	12120.4	2.3547	**5147.43**
	J20	VX-765	40378.6	2.3547	**17148.45**
	J20	VX-765	15787.4	2.3547	**6704.77**

20-week WO	J20	Vehicle	40056	5.1122	**7835.34**
	J20	Vehicle	18897	2.5561	**7392.87**
	J20	Vehicle	27179	5.1122	**5316.47**
	J20	Vehicle	21149	2.5561	**8273.89**
	J20	Vehicle	145508.5	2.3547	**61796.21**
	J20	Vehicle	57379.2	2.3547	**24368.45**
	J20	VX-765	13961	5.1122	**2730.91**
	J20	VX-765	38295	5.1122	**7490.87**
	J20	VX-765	23075	5.1122	**4513.69**
	J20	VX-765	28742	5.1122	**5622.21**
	J20	VX-765	87233.2	2.3547	**37047.19**
	J20	VX-765	83609	2.3547	**35508.03**

Figure 6d - Aβ EL

Hippo

		RIPA		
		**Total Aβ (pg/mg)**	**Aβ42/Total Aβ**	
J20	Vehicle	**148.59**	**0.10**	4-week WO
J20	Vehicle	**142.98**	**0.11**	
J20	Vehicle	**138.91**	**0.11**	
J20	Vehicle	**117.59**	**0.20**	
J20	Vehicle	**112.69**	**0.18**	
J20	Vehicle	**103.06**	**0.19**	
J20	Vehicle	**139.34**	**0.15**	
J20	VX-765	**177.93**	**0.09**	
J20	VX-765	**156.12**	**0.09**	
J20	VX-765	**126.17**	**0.10**	
J20	VX-765	**122.69**	**0.14**	
J20	VX-765	**126.03**	**0.15**	
J20	VX-765	**159.71**	**0.10**	
J20	VX-765	**255.50**	**0.11**	
J20	VX-765	**164.51**	**0.16**	12-week WO

J20	Vehicle	**137.99**	**0.09**	
J20	Vehicle	**130.95**	**0.09**	
J20	Vehicle	**128.36**	**0.10**	
J20	Vehicle	**109.48**	**0.18**	
J20	Vehicle	**137.34**	**0.16**	
J20	Vehicle	**133.03**	**0.17**	
J20	VX-765	**179.70**	**0.08**	
J20	VX-765	**183.82**	**0.08**	
J20	VX-765	**185.79**	**0.08**	20-week WO
J20	VX-765	**178.50**	**0.13**	
J20	VX-765	**174.58**	**0.15**	
J20	VX-765	**104.46**	**0.17**	
J20	VX-765	**105.53**	**0.20**	
J20	Vehicle	**155.58**	**0.11**	
J20	Vehicle	**135.92**	**0.11**	
J20	Vehicle	**161.64**	**0.11**	
J20	Vehicle	**136.07**	**0.11**	
J20	Vehicle	**222.44**	**0.14**	
J20	Vehicle	**138.60**	**0.18**	
J20	Vehicle	**211.08**	**0.14**	
J20	VX-765	**175.24**	**0.12**	
J20	VX-765	**171.15**	**0.12**	
J20	VX-765	**194.24**	**0.10**	
J20	VX-765	**190.82**	**0.11**	
J20	VX-765	**267.99**	**0.15**	
J20	VX-765	**204.68**	**0.22**	
J20	VX-765	**96.05**	**0.23**	

estern blot quantification (% of J20+veh)

WT+veh	J20+veh	J20+VX
**54.7**	**110.7**	**101.3**
**15.4**	**81.5**	**68.8**
**70.8**	**107.9**	**62.8**

219.2	201.8	333.2
121.6	74.5	262.9
230.7	65.3	83.3
1006.0	58.4	41.9
73.2	115.1	84.0
91.8	87.4	91.8
81.7	119.1	151.4
41.5	78.4	123.2
49.9	143.7	79.9
72.6	120.3	78.6
34.4	36.0	32.1
163.7		

ISA (Formic acid) See source data for Supplemental Figure 8 for additional data

		Formic acid			
		**Total Aβ (pg/mg)**	**Aβ42/Total Aβ**	**Cortex**	
J20	Vehicle	**472.93**	**0.12**	4-week WO	J20
J20	Vehicle	**473.34**	**0.11**		J20
J20	Vehicle	**142.13**	**0.40**		J20
J20	Vehicle	**532.48**	**0.54**		J20
J20	Vehicle	**420.63**	**0.51**		J20
J20	Vehicle	**247.21**	**0.32**		J20
J20	VX-765	**430.31**	**0.14**		J20
J20	VX-765	**621.87**	**0.29**		J20
J20	VX-765	**205.67**	**0.31**		J20
J20	VX-765	**240.34**	**0.49**		J20
J20	VX-765	**143.48**	**0.33**		J20
J20	VX-765	**116.59**	**0.43**		J20
J20	VX-765	**576.23**	**0.37**		J20
J20	Vehicle	**3337.80**	**0.32**	12-week WO	J20
J20	Vehicle	**3676.87**	**0.29**		J20

J20	Vehicle	**17893.65**	**0.54**		J20
J20	Vehicle	**15076.80**	**0.47**		J20
J20	VX-765	**3190.54**	**0.31**		J20
J20	VX-765	**3399.99**	**0.30**		J20
J20	VX-765	**15793.44**	**0.47**		J20
J20	VX-765	**65155.70**	**0.72**		J20
J20	VX-765	**1100.04**	**0.41**		J20
J20	Vehicle	**21426.92**	**0.37**	20-week WO	J20
J20	Vehicle	**14783.27**	**0.34**		J20
J20	Vehicle	**14051.62**	**0.37**		J20
J20	Vehicle	**15885.18**	**0.39**		J20
J20	Vehicle	**8267.19**	**0.33**		J20
J20	Vehicle	**62557.21**	**0.79**		J20
J20	Vehicle	**14991.65**	**0.34**		J20
J20	VX-765	**19335.78**	**0.34**		J20
J20	VX-765	**19986.79**	**0.36**		J20
J20	VX-765	**24398.42**	**0.33**		J20
J20	VX-765	**24829.00**	**0.35**		J20
J20	VX-765	**75115.44**	**0.64**		J20
J20	VX-765	**15918.63**	**0.34**		J20
J20	VX-765	**72642.60**	**0.65**		J20

	Formic acid	
	**Total Aβ (pg/mg)**	**Aβ42/Total Aβ**
Vehicle	**126.24**	**0.25**
Vehicle	**142.00**	**0.25**
Vehicle	**65.76**	**0.29**
Vehicle	**47.17**	**0.41**
Vehicle	**79.44**	**0.20**
Vehicle	**34.81**	**0.31**
VX-765	**17.25**	**0.30**
VX-765	**60.88**	**0.29**
VX-765	**124.32**	**0.32**
VX-765	**62.24**	**0.34**
VX-765	**59.82**	**0.20**
VX-765	**110.10**	**0.52**
VX-765	**48.92**	**0.16**
Vehicle	**569.64**	**0.55**
Vehicle	**603.49**	**0.61**

Vehicle	535.91	0.68
Vehicle	3398.88	0.63
VX-765	367.95	0.57
VX-765	465.31	0.60
VX-765	88.81	0.34
VX-765	398.27	0.53
VX-765	5210.80	0.54
Vehicle	1880.00	0.35
Vehicle	936.23	0.60
Vehicle	884.16	0.43
Vehicle	202.27	0.05
Vehicle	3426.50	0.48
Vehicle	2830.18	0.58
Vehicle	876.42	0.38
VX-765	783.58	0.02
VX-765	853.82	0.02
VX-765	553.83	0.01
VX-765	543.10	0.02
VX-765	8607.65	0.59
VX-765	5138.32	0.65
VX-765	657.81	0.71

Figure 7a - Iba1 vs NOR impairment correlation

	Hippo		Cortex	
	Iba1 cell density	NOR DI	Iba1 cell density	NOR DI
WT+veh	**5723.2**	**0.55**	**9576.6**	**0.55**
WT+veh	**5014.5**	**0.33**	**5443.5**	**0.33**
WT+veh	**5796.4**	**0.37**	**6675.6**	**0.37**
WT+veh	**3973.8**	**0.43**	**6048.4**	**0.43**
WT+veh	**6136**	**0.00**	**5846.8**	**0.00**
WT+veh	**4514.4**	**0.71**	**6317.2**	**0.71**
WT+veh	**4251.4**	**0.40**	**4267.5**	**0.40**
WT+veh	**3935.3**	**0.50**	**4939.5**	**0.50**
WT+veh	**4704.3**	**0.00**	**6989.2**	**0.00**
WT+veh	**6129**	**0.40**	**8568.5**	**0.40**
WT+veh	**5112.3**	**0.00**	**5645.2**	**0.00**
WT+veh	**5675**	**0.43**	**5645.2**	**0.43**
WT+veh	**8055.6**	**0.50**	**8666.7**	**0.50**
WT+veh	**6250**	**0.33**	**7888.9**	**0.33**
WT+veh	**7916.7**	**0.27**	**6666.7**	**0.27**
J20+veh	**8125.6**	**0.13**	**13407.3**	**0.13**
J20+veh	**7743.8**	**-0.04**	**13248.8**	**-0.04**
J20+veh	**8889.3**	**0.05**	**12500.0**	**0.05**
J20+veh	**11328.7**	**-0.03**	**12576.8**	**-0.03**
J20+veh	**11904.8**	**0.04**	**14112.9**	**0.04**
J20+veh	**9817.7**	**-0.08**	**10080.6**	**-0.08**
J20+veh	**7141.5**	**0.08**	**11592.7**	**0.08**
J20+veh	**7560.5**	**0.13**	**11895.2**	**0.13**
J20+veh	**13608.9**	**-0.07**	**10383.1**	**-0.07**
J20+veh	**8582.9**	**0.00**	**11962.4**	**0.00**
J20+veh	**9305.2**	**-0.16**	**9879.0**	**-0.16**
J20+veh	**9311.3**	**-0.02**	**10584.7**	**-0.02**
J20+veh	**12638.9**	**-0.25**	**13888.9**	**-0.25**
J20+veh	**12083.3**	**0.00**	**11555.6**	**0.00**
J20+veh	**13888.9**	**-0.04**	**13777.8**	**-0.04**

Figure 7b - IL-1β protein levels vs NOI

	Hippo	
	IL-1β (pg/mg)	NOR DI
WT+veh	**0.00**	**0.33**
WT+veh	**0.00**	**0.55**
WT+veh	**0.00**	**0.25**
WT+veh	**0.00**	**0.33**
WT+veh	**0.00**	**0.41**
WT+veh	**0.01**	**0.20**
WT+veh	**0.01**	**0.50**
WT+veh	**0.03**	**0.20**
WT+veh	**0.01**	**0.33**
WT+veh	**0.00**	**0.50**
WT+veh	**0.00**	**0.33**
WT+veh	**0.05**	**0.25**
WT+veh	**0.02**	**0.33**
WT+veh	**0.01**	**0.33**
WT+veh	**0.00**	**0.11**
WT+veh	**0.02**	**0.45**
WT+veh	**0.01**	**0.29**
J20+veh	**0.03**	**0.07**
J20+veh	**0.05**	**0.00**
J20+veh	**0.02**	**0.00**
J20+veh	**0.01**	**0.00**
J20+veh	**0.00**	**-0.08**
J20+veh	**0.00**	**-0.04**
J20+veh	**0.03**	**0.11**
J20+veh	**0.01**	**-0.27**
J20+veh	**0.02**	**-0.10**
J20+veh	**0.00**	**-0.16**
J20+veh	**0.01**	**-0.02**
J20+veh	**0.03**	**-0.08**
J20+veh	**0.09**	**0.45**

J20+VX	**4452.3**	**0.38**	**5443.5**	**0.38**
J20+VX	**4368.3**	**0.33**	**6955.6**	**0.33**
J20+VX	**5040.3**	**0.40**	**9173.4**	**0.40**
J20+VX	**8824.2**	**0.25**	**7889.2**	**0.25**
J20+VX	**10431.3**	**0.25**	**7100.3**	**0.25**
J20+VX	**8020.7**	**0.39**	**12096.8**	**0.39**
J20+VX	**5060.9**	**0.13**	**4334.7**	**0.13**
J20+VX	**5675**	**0.26**	**7762.1**	**0.26**
J20+VX	**5498.5**	**0.23**	**4334.7**	**0.23**
J20+VX	**6208.8**	**0.08**	**7459.7**	**0.08**
J20+VX	**7488.5**	**0.50**	**9072.6**	**0.50**
J20+VX	**4435.5**	**0.53**	**7762.1**	**0.53**
J20+VX	**7885.3**	**0.00**	**7056.5**	**0.00**
J20+VX	**10215.1**	**0.06**	**9530.8**	**0.06**
J20+VX	**11774.2**	**0.33**	**10080.6**	**0.33**

J20+veh	**0.02**	**-0.08**
J20+veh	**0.03**	**0.38**
J20+veh	**0.02**	**0.00**
J20+VX	**0.00**	**0.32**
J20+VX	**0.00**	**0.33**
J20+VX	**0.00**	**0.38**
J20+VX	**0.00**	**0.25**
J20+VX	**0.00**	**0.06**
J20+VX	**0.00**	**0.13**
J20+VX	**0.02**	**0.06**
J20+VX	**0.01**	**0.00**
J20+VX	**0.01**	**0.30**
J20+VX	**0.05**	**0.50**
J20+VX	**0.23**	**0.53**
J20+VX	**0.11**	**0.03**
J20+VX	**0.00**	**0.08**
J20+VX	**0.09**	**0.09**
J20+VX	**0.03**	**0.40**
J20+VX	**0.02**	**0.20**

[illegible] impairment correlation

Figure 7c - Aβ staining density vs NOR impairment correlation

Figure 7d

Cortex			Hippo		Cortex		
IL-1β (pg/mg)	NOR DI		Aβ density	NOR DI	Aβ density (	NOR DI	
**0.07**	**0.33**	J20+veh	**4766.6**	**0.13**	**510.5**	**0.13**	J20+veh
**0.09**	**0.55**	J20+veh	**3009.3**	**-0.04**	**259.8**	**-0.04**	J20+veh
**0.08**	**0.25**	J20+veh	**2659.2**	**0.05**	**358.4**	**0.05**	J20+veh
**0.10**	**0.33**	J20+veh	**6281.4**	**-0.03**	**260.7**	**-0.03**	J20+veh
**0.09**	**0.41**	J20+veh	**3424.0**	**0.04**	**370.1**	**0.04**	J20+veh
**0.18**	**0.20**	J20+veh	**3424.0**	**-0.08**	**367.7**	**-0.08**	J20+veh
**0.09**	**0.50**	J20+veh	**6802.5**	**0.08**	**2435.7**	**0.08**	J20+veh
**0.12**	**0.20**	J20+veh	**3510.0**	**0.13**	**4125.0**	**0.13**	J20+veh
**0.13**	**0.33**	J20+veh	**5010.7**	**-0.07**	**2223.7**	**-0.07**	J20+veh
**0.09**	**0.50**	J20+veh	**15673.8**	**0.00**	**3271.4**	**0.00**	J20+veh
**0.10**	**0.33**	J20+veh	**14905.8**	**-0.16**	**4502.5**	**-0.16**	J20+veh
**0.09**	**0.25**	J20+veh	**23773.0**	**-0.02**	**2628.6**	**-0.02**	J20+veh
**0.08**	**0.33**	J20+veh	**29939.4**	**-0.25**	**7835.3**	**-0.25**	J20+veh
**0.08**	**0.33**	J20+veh	**14653.5**	**0.00**	**7392.9**	**0.00**	J20+veh
**0.08**	**0.11**	J20+veh	**33488.0**	**-0.04**	**5316.5**	**-0.04**	J20+veh
**0.08**	**0.45**	J20+VX	**2122.8**	**0.38**	**437.1**	**0.38**	J20+veh
**0.10**	**0.29**	J20+VX	**2888.8**	**0.33**	**273.9**	**0.33**	J20+VX
**0.09**	**0.07**	J20+VX	**1740.9**	**0.40**	**703.2**	**0.40**	J20+VX
**0.07**	**0.00**	J20+VX	**1617.7**	**0.25**	**391.6**	**0.25**	J20+VX
**0.09**	**0.00**	J20+VX	**2224.2**	**0.25**	**390.4**	**0.25**	J20+VX
**0.06**	**0.00**	J20+VX	**1971.0**	**0.39**	**385.0**	**0.39**	J20+VX
**0.05**	**-0.08**	J20+VX	**6142.1**	**0.13**	**2122.8**	**0.13**	J20+VX
**0.06**	**-0.04**	J20+VX	**7067.0**	**0.26**	**1325.5**	**0.26**	J20+VX
**0.07**	**0.11**	J20+VX	**4167.3**	**0.23**	**1467.9**	**0.23**	J20+VX
**0.06**	**-0.27**	J20+VX	**7168.7**	**0.08**	**4115.6**	**0.08**	J20+VX
**0.06**	**-0.10**	J20+VX	**15356.9**	**0.50**	**3167.3**	**0.50**	J20+VX
**0.07**	**-0.16**	J20+VX	**6527.9**	**0.53**	**3608.6**	**0.53**	J20+VX
**0.06**	**-0.02**	J20+VX	**19803.9**	**0.00**	**2730.9**	**0.00**	J20+VX
**0.06**	**-0.08**	J20+VX	**22001.2**	**0.06**	**7490.9**	**0.06**	J20+VX
**0.09**	**0.45**	J20+VX	**25304.4**	**0.33**	**4513.7**	**0.33**	J20+VX

0.05	-0.08	J20+VX
0.09	0.38	J20+VX
0.07	0.00	
0.05	0.32	
0.06	0.33	
0.05	0.38	
0.07	0.33	
0.06	0.06	
0.06	0.13	
0.06	0.06	
0.07	0.00	
0.06	0.30	
0.08	0.50	
0.18	0.03	
0.11	0.53	
0.06	0.08	
0.09	0.09	
0.09	0.20	
0.08	0.40	

- total Aβ RIPA protein levels vs NOR impairment correlation

Hippo		Cortex	
Aβ RIPA (pg/mg)	NOR DI	Aβ RIPA (pg/mg)	NOR DI
**319.5**	**0.07**	**148.6**	**0.07**
**321.5**	**0.00**	**143.0**	**0.00**
**323.9**	**0.00**	**138.9**	**0.00**
**258.7**	**0.00**	**143.6**	**0.00**
**245.4**	**-0.08**	**138.7**	**-0.08**
**251.2**	**-0.04**	**132.8**	**-0.04**
**292.5**	**0.11**	**138.0**	**0.11**
**303.2**	**-0.27**	**130.9**	**-0.27**
**296.5**	**-0.10**	**128.4**	**-0.10**
**312.2**	**-0.16**	**176.5**	**-0.16**
**252.8**	**-0.02**	**119.6**	**-0.02**
**364.3**	**-0.08**	**154.4**	**-0.08**
**226.5**	**0.45**	**155.6**	**0.45**
**292.0**	**-0.08**	**135.9**	**-0.08**
**298.0**	**0.38**	**161.6**	**0.38**
**330.8**	**0.00**	**136.1**	**0.00**
**325.1**	**0.32**	**177.9**	**0.32**
**322.6**	**0.33**	**156.1**	**0.33**
**307.2**	**0.38**	**126.2**	**0.38**
**258.4**	**0.33**	**123.4**	**0.33**
**250.7**	**0.06**	**114.0**	**0.06**
**254.0**	**0.13**	**112.7**	**0.13**
**351.6**	**0.06**	**179.7**	**0.06**
**354.0**	**0.00**	**183.8**	**0.00**
**352.7**	**0.30**	**185.8**	**0.30**
**0.0**	**0.50**	**0.0**	**0.50**
**284.0**	**0.53**	**142.0**	**0.53**
**321.1**	**0.03**	**126.6**	**0.03**
**352.8**	**0.08**	**175.2**	**0.08**
**343.8**	**0.09**	**171.2**	**0.09**

354.6	0.40	194.2	0.40
350.2	0.20	190.8	0.20

Supplemental Fig 2a & d - NOR DI and open field distance travelled of repeatedly-measured mice at 8- and 16-wk W

		NOR DI score		Open field distance (m)	
Genotype	Treatment	8-week WO	16-week WO	8-week WO	16-week WO
WT	vehicle	0.75	0.50	13.22	13.30
WT	vehicle	0.67	0.64	5.13	4.60
WT	vehicle	-0.13	0.40	27.27	26.95
WT	vehicle	0.50	0.00	11.62	9.34
WT	vehicle	0.67	0.00	9.48	5.62
WT	vehicle	0.33	0.71	18.16	9.00
WT	vehicle	0.40	0.40	7.38	12.00
WT	vehicle	0.27	0.00	19.58	9.84
WT	vehicle	0.60	0.43	16.41	14.77
WT	vehicle	0.25	0.40	11.86	11.57
WT	vehicle	0.20	-0.11	18.86	14.17
WT	vehicle	0.33	0.33	11.64	15.00
WT	vehicle	0.41	0.33	43.17	16.10
WT	vehicle	0.05	0.33	22.51	36.66
J20	vehicle	0.14	0.08	54.28	53.61
J20	vehicle	-0.04	-0.06	25.94	30.74
J20	vehicle	-0.03	0.06	30.61	26.08
J20	vehicle	-0.06	-0.05	29.88	19.14
J20	vehicle	0.10	0.00	39.8	34.18
J20	vehicle	0.20	0.00	25.56	25.03
J20	vehicle	0.25	-0.16	11.51	24.91
J20	vehicle	0.02	-0.02	47.89	56.73
J20	vehicle	0.00	-0.08	65.05	24.19
J20	vehicle	-0.08	-0.09	38.46	25.27
J20	vehicle	-0.04	0.00	26.06	28.16
J20	VX-765	0.38	-0.03	47.41	55.96
J20	VX-765	0.67	0.20	53.39	37.38
J20	VX-765	0.25	-0.18	47.63	37.34

J20	VX-765	0.33	0.20		51.39	37.34
J20	VX-765	0.25	0.00		44.71	44.44
J20	VX-765	0.00	0.50		17.59	25.11
J20	VX-765	0.52	0.53		37.41	44.76
J20	VX-765	0.24	0.03		25.38	34
J20	VX-765	0.33	0.45		13.08	15.55
J20	VX-765	0.06	0.06		43.33	43.75

Supplemental Fig 1b & e - NOR DI and open field distance travelled of remaining NON-repeatedly-measured mice

		NOR DI score				Open field distance (m)			
Genotype	Treatment	4-week WO	8-week WO	12-week WO	16-week WO	4-week WO	8-week WO	12-week WO	16-week WO
WT	vehicle	0.37		0.41		26.91		23.72	
WT	vehicle	0.22				26.89			
WT	vehicle	0.20				20.1			
WT	vehicle	0.31				27.42			
WT	vehicle	0.17		0.27		29.92		20.12	
WT	vehicle	0.23				39.77			
WT	vehicle	0.33		0.33		29.84		14.32	
WT	vehicle	0.29				33.58			
WT	vehicle	0.27				30.39			
WT	vehicle	0.44		0.29		26.73		9.08	
WT	vehicle	0.19		0.33		30.02		17.29	
WT	vehicle	0.30		0.33		30.53		9.77	
WT	vehicle	0.20		0.33		16.67		10.18	
WT	vehicle	0.20				30.84			
WT	vehicle	0.29				28.17			
WT	vehicle	0.27				24.58			
WT	vehicle	0.45				33.27			
WT	vehicle	0.25		0.25		29.28		10.17	
WT	vehicle	0.28		0.27		30.15		10.02	
WT	vehicle	0.33		0.33		21.41		10.58	
WT	vehicle					4.11			
WT	vehicle	0.48	-0.25	-0.14		18.15	15.66	9.98	

WT	vehicle	0.55				19.5			
WT	vehicle	0.33	0.60	0.78		16.78	14.96	16.96	
WT	vehicle	0.37	0.57	0.45		16.82	38.67	24.5	
WT	vehicle	0.70	0.43			40.49	21.19		
WT	vehicle	0.60	0.00	0.33	0.33	15.23	31.47	21.63	20.67
WT	vehicle	0.36	0.71			22.74	23.81		
WT	vehicle	0.36	0.50	0.40	0.29	13.59	25.61	22.08	19.15
WT	vehicle	0.20				33.52			
WT	vehicle	0.25				17.32			
WT	vehicle	0.25	0.38			7.89	14.05		
WT	vehicle	0.71	0.64			11.68	16.35		
WT	vehicle	0.60	0.60	0.33		14.13	9.46	14.9	
WT	vehicle	0.17				15.14			
WT	vehicle	0.50	0.33	0.33		13.86	10.26	15.68	
WT	vehicle	0.43	0.38			27.07	19.12		
WT	vehicle	0.60	0.67			10.52	2.99		
WT	vehicle	0.50				15.79			
WT	vehicle	0.29	0.33	0.18		19.24	9.16	17.12	
WT	vehicle	0.50	0.33	0.50		13.7	12.29	14.1	
WT	vehicle	0.33	0.44	0.38		19.31	20.23	19.6	
WT	vehicle		0.20	0.20	0.25	40.33	9.93		

Supplemental Fig 1f,g,h - Open field secondary measures (# of quadrant entries, % time moving, thigmotaxis)

		OF # of quadrant entries							OF
**Genotype**	**Treatment**	**4-week WO**	**8-week WO**	**12-week WO**	**16-week WO**	**20-week WO**		**4-week WO**	**8-week WO**
WT	vehicle	166		159				88.6	
WT	vehicle	163						88.2	
WT	vehicle	133						79.3	
WT	vehicle	158						91.2	
WT	vehicle	189		137				89.8	
WT	vehicle	242						92.4	
WT	vehicle	186		93				90.4	
WT	vehicle	217						89.5	

WT	vehicle	190						92.4	
WT	vehicle	152		56				89.7	
WT	vehicle	194		108				88.9	
WT	vehicle	145		69		57		85.6	
WT	vehicle	127		66		56		80.7	
WT	vehicle	133		35		16		81.7	
WT	vehicle	183		163		148		87.9	
WT	vehicle	186		63				91.8	
WT	vehicle	114		77				71.6	
WT	vehicle	168		111		54		90.9	
WT	vehicle	186		102		84		89.5	
WT	vehicle	150		126		86		84.4	
WT	vehicle	196						93.3	
WT	vehicle	171						89.9	
WT	vehicle	150						89.9	
WT	vehicle	190						91.8	
WT	vehicle	178		116		77		93.2	
WT	vehicle	175		133		72		90.7	
WT	vehicle	237		184		129		93.7	
WT	vehicle	165		55				91.1	
WT	vehicle	192		62				91.5	
WT	vehicle	127		69				87.9	
WT	vehicle	331						93.9	
WT	vehicle	209	84	58				87.7	69.2
WT	vehicle	161	87	46	65	58		80.7	68.1
WT	vehicle	185	32	21	28	53		86.3	29.6
WT	vehicle	393						91.6	
WT	vehicle	152	90	98				87.1	66.9
WT	vehicle	222	232	141				95.2	95.2
WT	vehicle	292	170	214	167	125		94.0	82.2
WT	vehicle	192	132					87.8	67.0
WT	vehicle	184	190	127	129			74.6	89.3
WT	vehicle	236	153					89.7	83.5

WT	vehicle	259	148	135	114			95.0	80.3
WT	vehicle	194						87.2	
WT	vehicle	104						70.6	
WT	vehicle	46	90					46.7	59.5
WT	vehicle	80	102					55.2	66.0
WT	vehicle	87	63	104				59.9	38.5
WT	vehicle	84						63.6	
WT	vehicle	99	66	89				61.3	46.3
WT	vehicle	172	113					78.1	55.9
WT	vehicle	62	13					44.7	12.8
WT	vehicle	113						70.6	
WT	vehicle	122	53	129				77.1	54.2
WT	vehicle	89	60	77				59.9	46.9
WT	vehicle	123	133	135				73.1	65.5
WT	vehicle	146	78	85	44	38		87.3	62.1
WT	vehicle	95	67	64	32	71		67.7	55.6
WT	vehicle	92	122	52	55	47		63.3	62.2
WT	vehicle	79	46	75	73	72		65.2	37.3
WT	vehicle	128	120	48	47	64		84.2	66.9
WT	vehicle	113	102	102	104	85		71.1	62.2
WT	vehicle	121	84	129	69	78		83.3	54.9
WT	vehicle	111	106	122	79	76		79.7	71.3
WT	vehicle	190	69	84	81	64		92.0	49.0
WT	vehicle	190	241	137	84	33		93.5	94.3
WT	vehicle	251	54					91.6	47.0
WT	vehicle	292	169	155	219	177		90.1	74.1
WT	VX-765	112		97				73.4	
WT	VX-765	99		89				76.5	
WT	VX-765	132		95				87.0	
WT	VX-765	115						78.5	
WT	VX-765	131						82.1	
WT	VX-765	203						92.1	

WT	VX-765	142			85.9
WT	VX-765	221			93.8
WT	VX-765	141	86		88.3
WT	VX-765	178	90		86.3
WT	VX-765	155			85.9
WT	VX-765	224			92.6
WT	VX-765	178			91.3
WT	VX-765	131	95		83.5
WT	VX-765	149	106		85.8
WT	VX-765	143	28		75.6
WT	VX-765	206			93.5
WT	VX-765	145	67	43	89.1
WT	VX-765	155	111	81	79.0
WT	VX-765	189	84		87.2
WT	VX-765	150	117	82	87.7
WT	VX-765	112	62	32	68.6
WT	VX-765	143	61	71	83.7
WT	VX-765	162	107	94	85.7
WT	VX-765	124	86	82	82.6
WT	VX-765	137	64	85	78.3
WT	VX-765	159	121	91	89.6
WT	VX-765	153			81.9
WT	VX-765	126			74.6
WT	VX-765	136			80.6
WT	VX-765	189	172		90.5
WT	VX-765	237	93		92.8
WT	VX-765	176	138	100	89.5
WT	VX-765	235	180	137	92.7
WT	VX-765	175	132	85	89.8
WT	VX-765	201	221	186	93.3
WT	VX-765	198			94.2
WT	VX-765	168			90.5
WT	VX-765	207			93.3

WT	VX-765	198		88			91.3	
WT	VX-765	163		132			88.8	
WT	VX-765	188		129			92.3	
J20	vehicle	201					92.1	
J20	vehicle	265		269			94.3	
J20	vehicle	251					95.5	
J20	vehicle	315		287			95.6	
J20	vehicle	214		191		309	92.4	
J20	vehicle	139					82.6	
J20	vehicle	204		206		184	91.1	
J20	vehicle	261		266			94.9	
J20	vehicle	239		374		375	91.7	
J20	vehicle	180		166			93.5	
J20	vehicle	283		289			94.5	
J20	vehicle	283					96.1	
J20	vehicle	269					94.8	
J20	vehicle	217		270			91.6	
J20	vehicle	223		221			94.1	
J20	vehicle	136	263				75.5	91.3
J20	vehicle	260	223				93.2	96.3
J20	vehicle	122	397	306			77.9	95.4
J20	vehicle	40					29.6	
J20	vehicle	607					98.1	
J20	vehicle	77	304	248	321	208	54.8	86.5
J20	vehicle	221	350	316			91.4	95.1
J20	vehicle	124	159	110	183	180	75.8	89.8
J20	vehicle	321	178	116	145	98	90.5	82.8
J20	vehicle	93	206	465	604		65.1	87.5
J20	vehicle	111	797	625	690		79.1	99.7
J20	vehicle	326	619	622	579		77.5	97.7
J20	vehicle	198					84.3	
J20	vehicle	137	175	103	117	114	76.1	74.3

J20	vehicle	96	248	234	207	155		70.7	94.2
J20	vehicle	157	120					87.7	65.5
J20	vehicle	103	144	145	139	136		70.3	76.2
J20	vehicle	163	65	108	135	112		71.9	52.3
J20	vehicle	149	290	280	326	395		85.9	94.7
J20	vehicle	94	389	306	127	40		68.2	94.7
J20	vehicle	182	235	257	146	162		87.4	82.0
J20	vehicle	118	158	258	153	178		58.3	81.5
J20	vehicle	529	77	303	122	266		96.3	60.3
J20	VX-765	308	127					98.1	81.5
J20	VX-765	20						22.6	
J20	VX-765	91	299					74.5	95.1
J20	VX-765	171						70.9	
J20	VX-765	142	262	233	330	212		83.2	95.5
J20	VX-765	140	302	273	207	193		79.4	91.5
J20	VX-765	472	121	141				94.0	78.5
J20	VX-765	196	243	134	130			75.8	88.7
J20	VX-765	246	84	183				92.4	55.5
J20	VX-765	122	272	242	234	394		95.7	99.2
J20	VX-765	198	322	229	311			88.3	95.0
J20	VX-765	103	226	242	245			56.7	91.3
J20	VX-765	132						72.1	
J20	VX-765	253						91.1	
J20	VX-765	142						87.9	
J20	VX-765	195	304	230	210	85		89.2	92.4
J20	VX-765	71	259	216	258	207		58.9	90.8
J20	VX-765	~~2~~						~~0.2~~	
J20	VX-765	82	139	154	127	~~0~~		61.5	64.2
J20	VX-765	71	93	141	125	146		58.8	44.5
J20	VX-765	188	203	154	247	255		87.1	78.6
J20	VX-765	153	150	184	198	133		79.9	70.0
J20	VX-765	92	65	67	84	69		54.9	42.9

J20	VX-765	258	259	223	251	275		94.1	84.9
J20	VX-765	143	163	181	138	159		47.1	78.5

Supplemental Figure 1i - Barnes maze learning acquisition latency to target

12-week WO		Learning acquisition latency to target (s)				20-week WO		Learnir
Genotype	Treatment	Day 1	Day 2	Day 3	Day 4	Genotype	Treatment	Day 1
WT	Vehicle	82.8	68.5	28.0	15.3	WT	Vehicle	45.0
WT	Vehicle	29.3	34.3	15.0	20.0	WT	Vehicle	44.3
WT	Vehicle	152.3	123.5	24.3	29.8	WT	Vehicle	98.0
WT	Vehicle	75.0	19.0	27.0	15.0	WT	Vehicle	34.3
WT	Vehicle	82.5	63.5	40.3	48.8	WT	Vehicle	43.3
WT	Vehicle	123.8	40.0	64.5	23.8	WT	Vehicle	65.8
WT	Vehicle	180.0	100.5	10.5	25.5	WT	Vehicle	48.0
WT	Vehicle	39.3	58.0	44.8	19.0	WT	Vehicle	38.3
WT	Vehicle	180.0	76.5	26.8	22.5	WT	Vehicle	61.3
WT	Vehicle	139.0	53.8	44.8	36.8	WT	Vehicle	49.5
WT	Vehicle	135.3	59.3	24.3	10.3	WT	Vehicle	75.5
WT	Vehicle	62.8	71.8	50.3	76.8	WT	Vehicle	18.3
WT	Vehicle	133.8	62.0	39.0	26.5	WT	Vehicle	51.8
WT	Vehicle	99.3	29.0	26.3	17.5	WT	Vehicle	71.3
WT	Vehicle	168.8	39.3	48.0	21.8	WT	Vehicle	53.8
WT	Vehicle	96.0	46.5	30.5	30.8	WT	Vehicle	67.5
WT	Vehicle	171.0	80.5	30.0	27.3	WT	Vehicle	34.8
WT	Vehicle	87.3	138.5	41.5	37.5	WT	Vehicle	38.0
WT	Vehicle	117.3	55.5	82.3	58.3	WT	Vehicle	74.8
WT	Vehicle	180.0	109.8	93.3	48.3	WT	Vehicle	41.5
WT	Vehicle	102.3	43.3	54.0	11.5			
WT	Vehicle	43.0	34.0	40.8	29.5			
WT	VX-765	84.3	30.3	26.5	16.8	WT	VX-765	79.8
WT	VX-765	92.5	41.8	22.3	19.8	WT	VX-765	74.0
WT	VX-765	99.8	37.5	18.3	20.5	WT	VX-765	34.8
WT	VX-765	90.8	75.3	17.0	16.0	WT	VX-765	23.8

WT	VX-765	128.8	58.0	28.0	10.0	WT	VX-765	47.5
WT	VX-765	80.5	76.5	16.8	13.5	WT	VX-765	26.3
WT	VX-765	82.0	43.5	21.3	18.5	WT	VX-765	83.8
WT	VX-765	100.3	94.8	35.5	15.8	WT	VX-765	89.8
WT	VX-765	128.8	63.0	59.5	20.5	WT	VX-765	21.3
WT	VX-765	67.3	24.8	36.5	11.8	WT	VX-765	64.5
WT	VX-765	89.8	19.8	12.8	13.8	WT	VX-765	26.8
WT	VX-765	96.8	48.8	16.5	12.0	WT	VX-765	65.5
WT	VX-765	34.0	36.0	18.5	10.3	WT	VX-765	15.0
J20	vehicle	94.3	52.0	28.8	35.5	J20	vehicle	72.5
J20	vehicle	83.8	68.5	23.0	22.0	J20	vehicle	47.5
J20	vehicle	99.0	27.3	38.5	16.3	J20	vehicle	43.0
J20	vehicle	138.5	104.5	61.0	36.5	J20	vehicle	128.0
J20	vehicle	180.0	180.0	165.0	138.3	J20	vehicle	62.0
J20	vehicle	155.8	44.5	20.0	22.3	J20	vehicle	48.8
J20	vehicle	105.0	168.8	125.8	32.0	J20	vehicle	65.0
J20	vehicle	180.0	63.5	23.8	37.8	J20	vehicle	46.0
J20	vehicle	157.3	74.5	34.0	29.3	J20	vehicle	21.8
J20	vehicle	96.3	15.3	22.3	18.3	J20	vehicle	31.3
J20	vehicle	89.8	43.5	21.8	10.8	J20	vehicle	21.8
J20	vehicle	55.3	21.0	20.0	14.3			
J20	vehicle	45.5	26.3	24.3	15.5			
J20	vehicle	56.3	25.8	19.0	15.0			
J20	vehicle	46.0	24.0	18.5	11.8			
J20	VX-765	180.0	174.0	96.5	24.0	J20	VX-765	61.0
J20	VX-765	86.3	29.0	29.0	23.0	J20	VX-765	51.3
J20	VX-765	75.3	62.8	46.8	27.5	J20	VX-765	82.5
J20	VX-765	149.8	79.0	43.8	16.5	J20	VX-765	60.8
J20	VX-765	98.0	36.5	23.3	17.0	J20	VX-765	55.3
J20	VX-765	147.3	45.8	35.5	31.0	J20	VX-765	39.3
J20	VX-765	80.8	43.8	40.8	22.5	J20	VX-765	38.8

J20	VX-765	86.8	139.8	34.0	32.5
J20	VX-765	59.0	25.3	24.5	14.5
J20	VX-765	127.8	45.8	49.8	22.8

/O

		NOR DI score				Open field distance (m)		
Genotype	Treatment	4-week WO	8-week WO	12-week WO	16-week WO	4-week WO	8-week WO	12-week WO
WT	VX-765	0.24		0.43		18.66		17.99
WT	VX-765	0.33		0.43		16.01		13.08
WT	VX-765	0.30		0.50		22.89		16.55
WT	VX-765	0.25				19.89		
WT	VX-765	0.33				20.73		
WT	VX-765	0.60				32.47		
WT	VX-765	0.50				22.27		
WT	VX-765	0.22				35.73		
WT	VX-765	0.22		0.50		21.78		14.54
WT	VX-765	0.33		0.54		27.83		13.12
WT	VX-765	0.35				26.68		
WT	VX-765	0.07				38.87		
WT	VX-765	0.36				27.56		
WT	VX-765	0.38		0.33		20.85		14.79
WT	VX-765	0.43		0.50		24.32		18.03
WT	VX-765	0.65		0.50		22.03		5.92
WT	VX-765	0.38				32.72		
WT	VX-765	0.25		0.40		29.90		12.97
WT	VX-765	0.12				23.43		
WT	VX-765	0.58				18.78		
WT	VX-765	0.30				22.17		
WT	VX-765	0.31		0.16		31.06		28.49

WT	VX-765	0.50	0.80	40.02	18.04
WT	VX-765	0.39		34.00	
WT	VX-765	0.45		28.41	
WT	VX-765	0.38		36.13	
WT	VX-765	0.20	0.58	32.25	14.57
WT	VX-765	0.25	0.50	23.57	19.78
WT	VX-765	0.22	0.33	31.41	18.96

% time moving			OF thigmotaxis (% time in periphery)				
12-week WO	16-week WO	20-week WO	4-week WO	8-week WO	12-week WO	16-week WO	20-week WO
80.6			63.0		79.7		
			81.4				
			79.6				
			73.9				
76.9			84.2		71.7		
			81.7				
65.9			69.7		91.3		
			79.5				

			78.4					
52.3			65.4			91.9		
68.0			63.5			83.3		
48.6		39.2	75.9			97.5		98.7
53.3		37.4	57.7			53.8		76.6
41.7		14.1	86.5			98.9		100.0
78.7		64.9	80.0			83.6		70.8
48.1			75.1			95.7		
50.1			67.9			94.0		
70.0		38.6	76.4			93.4		95.4
70.3		54.2	80.6			81.9		95.0
72.2		49.4	81.7			93.9		79.3
			77.1					
			77.6					
			73.4					
			87.5					
72.5		46.0	74.8		93.2			96.7
78.0		45.4	77.6		87.8			88.9
85.6		72.8	78.6		89.6			86.6
56.2			74.1		96.6			
53.3			64.4		88.5			
48.8			70.8		98.2			
			80.5					
62.0			92.5	93.9		77.6		
46.3	58.5	50.9	89.5	85.7		99.0	98.0	97.5
14.5	21.9	32.5	93.3	83.8		99.7	100.0	99.6
			89.3					
64.5			94.6	94.0		95.8		
87.2			82.0	80.4		88.5		
86.2	80.1	69.6	94.1	92.3		92.4	93.7	94.5
			95.7	94.8				
73.9	67.9		81.7	88.6		93.2	95.7	
			91.4	92.7				

73.1	67.8		94.8	85.0	84.9	91.8	
			91.6				
			95.0				
			83.0	82.3			
			86.4	81.8			
59.7			64.5	87.3	71.2		
			96.6				
62.1			57.4	77.0	98.6		
			77.9	92.6			
			69.8	100.0			
			66.3				
78.7			48.6	43.1	58.7		
58.7			91.1	99.9	98.4		
73.4			73.2	59.4	86.8		
50.9	50.1	37.8	89.6	92.9	94.1	94.2	99.5
47.6	30.6	45.5	69.0	82.0	96.2	96.1	93.1
36.6	36.6	43.9	87.0	91.8	99.0	99.1	99.2
50.1	56.2	53.3	58.6	97.1	93.7	87.6	94.5
36.1	39.1	42.9	82.2	93.0	99.3	98.7	97.9
58.7	59.1	60.5	57.3	95.1	93.9	95.5	92.5
63.5	56.5	51.6	82.1	78.1	84.3	65.0	86.8
73.5	55.9	60.9	90.7	95.7	93.3	97.7	97.9
49.1	53.2	45.8	89.8	97.6	99.1	96.4	97.4
79.5	66.6	35.0	90.0	96.3	98.1	99.5	98.9
			61.5	42.5			
76.7	85.6	68.5	82.6	83.3	76.2	90.9	70.9
64.6			74.8		80.0		
55.7			74.8		79.5		
72.3			66.6		86.9		
			66.4				
			48.8				
			80.4				

		78.3		
		79.8		
65.2		82.2	91.6	
60.1		65.4	62.5	
		87.1		
		84.3		
		65.2		
62.2		75.0	89.3	
63.7		63.8	66.1	
32.3		82.0	98.8	
		80.7		
56.1	30.9	69.1	97.1	99.0
70.9	51.1	47.4	67.8	86.3
57.9		77.5	89.5	
66.4	48.1	71.0	82.3	92.4
44.3	19.5	85.7	98.7	99.4
51.1	48.4	71.2	96.3	90.7
64.6	51.7	69.1	71.9	84.6
62.9	36.7	69.1	97.8	96.0
47.5	42.7	78.8	56.0	50.3
71.1	59.6	72.6	86.6	95.2
		79.1		
		76.1		
		74.7		
83.1		78.5	87.8	
75.1		78.2	97.7	
77.9	56.9	78.2	84.1	92.1
85.3	67.8	88.1	85.8	68.9
82.3	64.3	89.9	73.8	78.3
91.9	82.9	87.0	78.5	81.0
		71.8		
		66.6		
		85.9		

67.8			71.8		90.6		
77.5			56.4		84.6		
73.7			71.4		81.5		
			90.6				
93.3			90.9		90.3		
			88.6				
95.9			77.3		75.9		
89.1		96.3	90.8		92.7		87.7
			86.2				
88.9		85.2	87.1		96.2		92.0
96.8			92.4		86.7		
95.5		96.5	91.9		88.2		86.0
89.5			91.9		92.8		
93.0			85.3		81.0		
			87.9				
			85.5				
94.3			89.6		89.1		
93.5			84.0		89.4		
			98.5	87.6			
			96.6	92.8			
94.8			68.9	92.6	87.4		
			92.2				
			85.6				
92.2	93.3	82.0	76.9	97.9	81.3	90.6	92.3
96.6			95.9	78.8	84.0		
73.0	87.1	85.6	80.8	90.2	96.7	95.5	95.9
62.0	74.8	57.1	97.4	94.8	99.0	97.2	97.4
94.4	97.1		95.2	90.6	81.8	60.2	
99.1	99.3		90.5	85.3	97.5	88.7	
94.1	83.7		99.8	90.7	82.2	90.0	
			91.5				
63.3	59.0	59.5	96.9	97.6	98.8	98.3	98.3

85.9	82.7	71.3	99.2	88.2	95.7	91.8	96.4
			94.6	97.9			
69.2	68.1	69.7	98.8	96.0	98.2	95.1	97.4
68.6	73.3	58.3	94.6	91.3	98.1	96.1	98.8
93.3	94.9	97.4	92.5	96.1	98.2	94.9	88.4
93.2	55.1	25.7	97.2	95.8	95.7	89.2	95.8
80.0	68.8	70.1	97.2	95.2	97.4	98.1	97.2
95.1	68.0	75.0	98.6	93.1	96.1	97.4	97.8
96.3	55.0	82.5	95.6	88.8	83.9	63.2	88.6
			99.2	91.9			
			94.7				
			90.1	89.4			
			98.0				
92.9	92.9	91.4	95.7	93.0	98.1	93.4	93.6
88.6	73.1	83.5	89.8	96.9	95.9	98.0	94.6
82.1			92.3	97.6	92.7		
81.5	73.9		99.4	90.6	98.2	97.7	
68.5			84.5	95.1	97.1		
98.3	98.5	95.1	89.9	76.6	84.8	97.3	87.2
89.8	94.1		93.5	74.7	82.1	83.7	
94.6	89.5		94.3	95.2	92.1	97.6	
			97.4				
			95.7				
			91.5				
86.1	84.3	35.9	89.1	97.0	94.4	95.1	99.3
79.1	85.4	86.3	94.1	96.9	98.9	94.3	95.5
			~~0.2~~				
72.5	66.3	~~0.3~~	97.2	97.5	98.1	99.1	~~100.0~~
74.5	68.1	68.2	96.6	99.6	99.0	99.9	98.9
70.0	87.4	90.1	92.7	98.0	94.1	96.2	92.7
60.4	84.2	71.0	94.6	96.2	94.2	90.8	97.0
47.1	54.4	46.0	96.6	99.4	99.9	99.2	99.1

89.5	88.4	96.8		90.7	91.2	96.9	92.8	93.8
72.0	69.5	63.1		99.0	94.5	89.9	97.7	97.6

ıg acquisition latency to target (s)

Day 2	Day 3	Day 4
14.8	38.0	10.3
19.0	18.3	9.5
27.8	41.0	38.5
30.8	25.0	16.8
40.3	37.8	15.8
31.8	37.0	29.0
50.5	94.8	109.8
34.5	19.5	19.0
14.0	10.0	10.0
34.8	44.3	29.0
73.8	26.3	15.8
21.0	11.3	11.0
33.3	20.3	22.0
25.0	20.5	23.5
32.5	32.5	13.3
50.0	24.8	26.0
33.8	10.5	11.8
48.5	23.3	16.0
40.0	11.0	15.0
17.8	21.5	12.3
18.5	20.0	11.0
21.3	30.8	21.3
13.8	19.3	7.5
30.5	42.8	9.8

25.8	21.0	13.8
27.8	20.3	10.0
18.0	23.3	11.5
18.8	11.0	13.8
15.0	43.3	9.5
15.0	8.0	7.5
15.3	10.0	9.5
15.5	16.5	8.0
14.3	10.5	13.5

41.8	39.3	17.0
58.8	86.0	27.3
15.0	13.8	24.0
154.5	122.8	43.5
56.0	70.3	20.5
65.8	49.3	44.0
70.3	33.5	30.5
28.3	29.0	14.0
25.8	15.8	19.3
13.8	16.8	15.0
16.8	12.8	18.8

21.0	20.0	22.8
27.3	38.8	19.5
20.3	20.8	12.3
34.3	18.5	18.8
40.5	30.3	29.3
27.5	18.0	12.0
18.8	19.5	25.3

			NOR DI score				Open field c	
16-week WO	Genotype	Treatment	4-week WO	8-week WO	12-week WO	16-week WO	4-week WO	8-week WO
	J20	vehicle	-0.07				33.96	
	J20	vehicle	0.00		0.00		48.32	
	J20	vehicle	-0.18				42.49	
	J20	vehicle	0.08		0.00		50.63	
	J20	vehicle	0.03				24.49	
	J20	vehicle	-0.03		-0.06		46.24	
	J20	vehicle	0.00		0.06		31.22	
	J20	vehicle	0.08		0.08		48.02	
	J20	vehicle	0.00				48.41	
	J20	vehicle	0.03				48.09	
	J20	vehicle	0.17		0.00		39.65	
	J20	vehicle	0.25		0.00		39.23	
	J20	vehicle	0.02	-0.03			86.47	46.09
	J20	vehicle	0.00	-0.03			59.4	38.88
	J20	vehicle	-0.02	0.05	-0.05		54.17	66.45
	J20	vehicle	0.13				36.62	
	J20	vehicle	-0.04				37.51	
	J20	vehicle	0.15	-0.06	0.06		57.68	56.99
	J20	vehicle	0.00	0.04	0.03	0.07	29.94	33.6
	J20	vehicle	-0.06	-0.08	0.08	-0.07	67.8	136.65
	J20	vehicle	0.00	0.10	0.13	-0.19	108.94	102.37
	J20	vehicle	0.00				35.11	

J20	vehicle	0.05	-0.23	29.05	21.25
J20	vehicle	difficult to handle		93.08	12.53

listance (m)				NOR DI score				
12-week WO	16-week WO	Genotype	Treatment	4-week WO	8-week WO	12-week WO	16-week WO	4-week WO
		J20	VX-765	0.47	0.19			39.35
47.87		J20	VX-765	0.33				26.91
		J20	VX-765	0.08	0.41			46.66
48.3		J20	VX-765	0.38				39.27
		J20	VX-765	-0.05	0.67	0.50		24.25
45.45		J20	VX-765	0.53	0.30	0.41	0.29	31.51
28.42		J20	VX-765	0.40	0.25	0.23		30.87
45.05		J20	VX-765	0.45	0.39	0.13	-0.03	47.24
		J20	VX-765	0.58	0.21	0.26	0.16	29.62
		J20	VX-765	0.53				23.32
45.68		J20	VX-765	0.36				46.9
38.63		J20	VX-765	0.12				23.46
		J20	VX-765					~~0.15~~
		J20	VX-765	0.53	0.29	0.06	0.08	15.13
49.59		J20	VX-765	0.29	0.33		-0.20	26.37
54.63								
76.89	102.99							
111.74	117.78							
103.98	100.58							

50.48	16.84

Open field distance (m)		
8-week WO	12-week WO	16-week WO
24.72		
50.38		
22.92	24.82	
40.98	27.53	21.89
16.15	34.09	
53.5	39.31	50.11
39.23	42.6	43.15
24.03	27.48	23.29
27.16	32.32	27.09

Supplemental Fig 2a - hAPP western blots in peripheral tissue

Antibody: 6E10, Biolegend,803001, lot B198895 (1:1000)

Liver

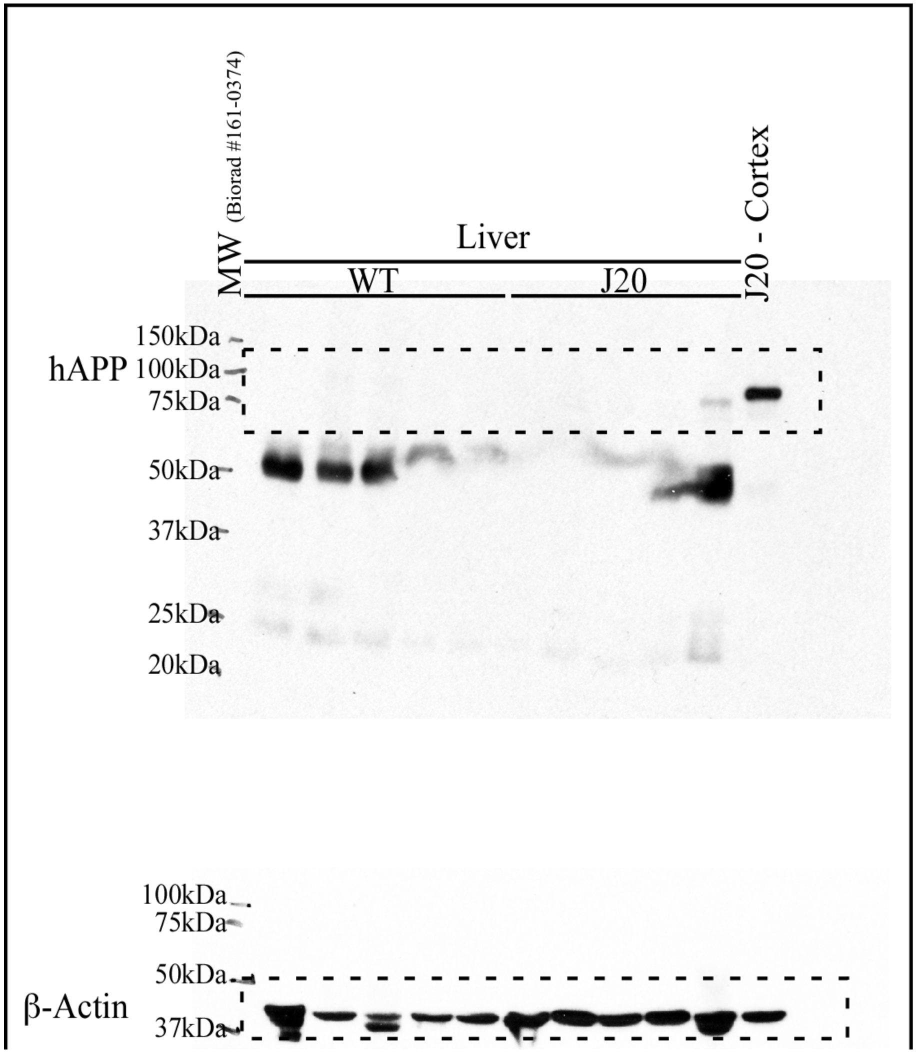

Kidney

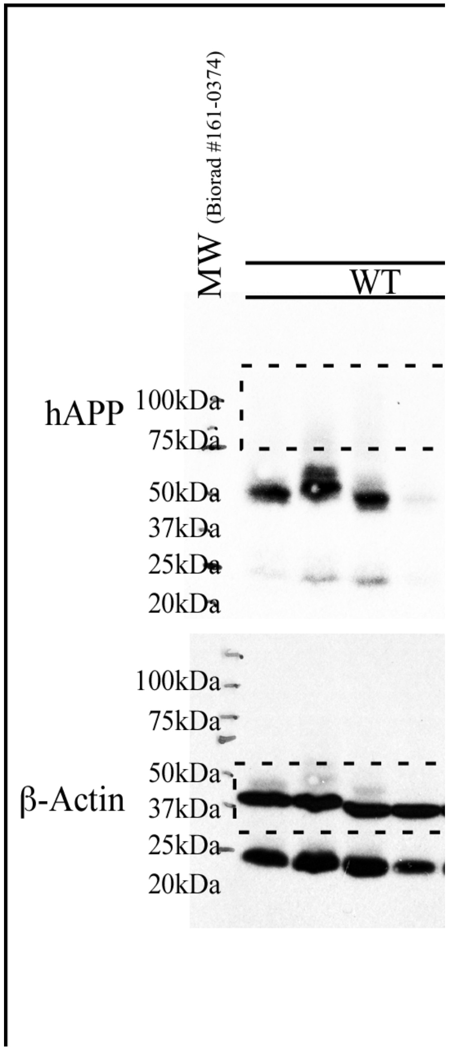

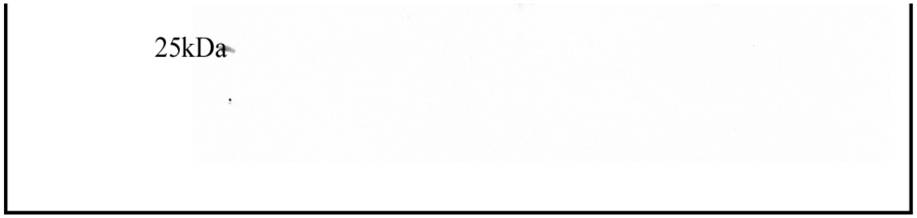

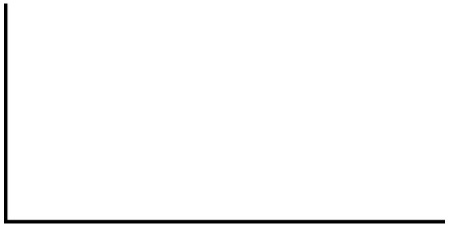

Spleen

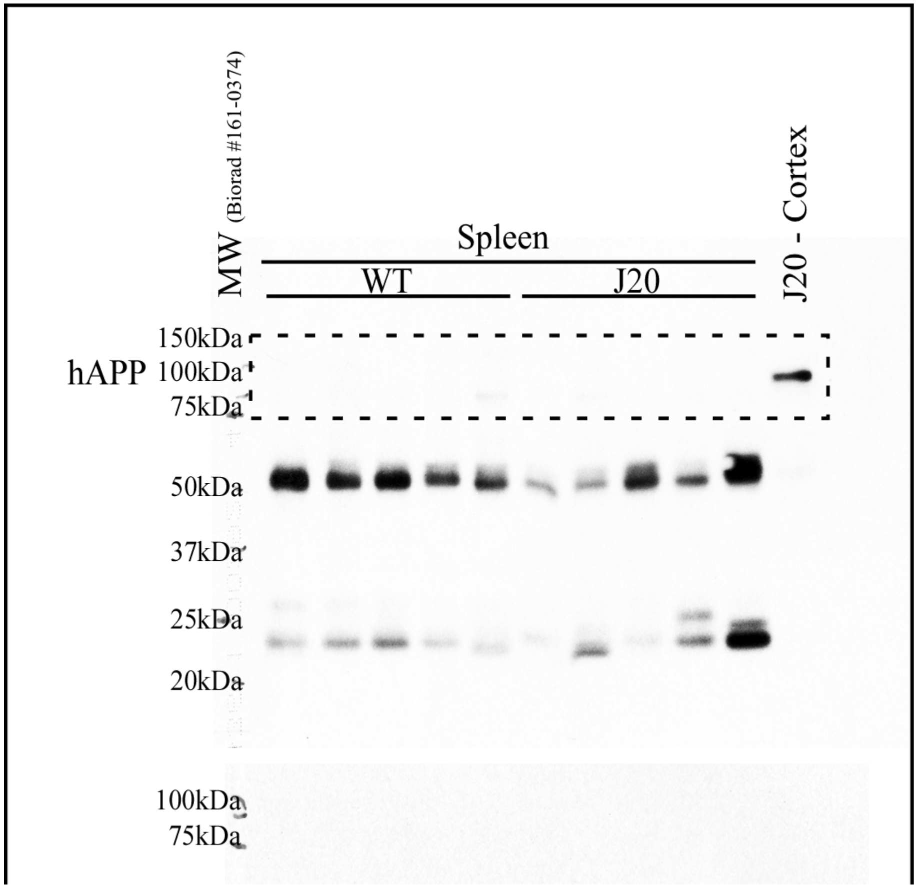

Heart

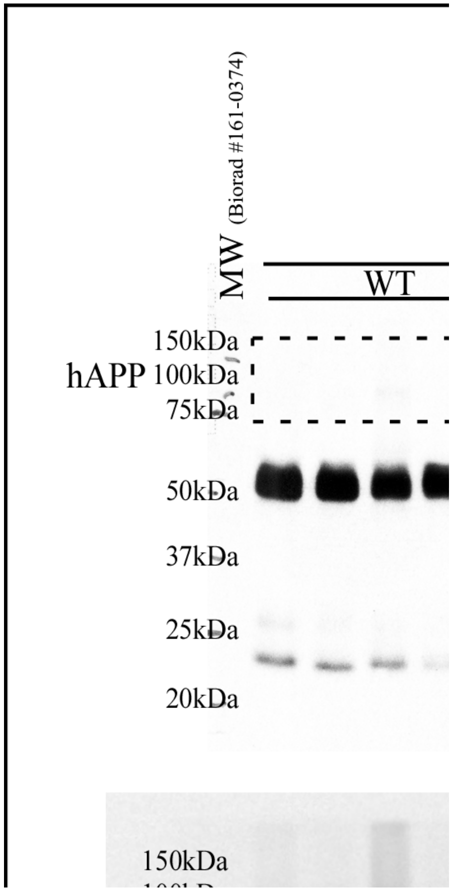

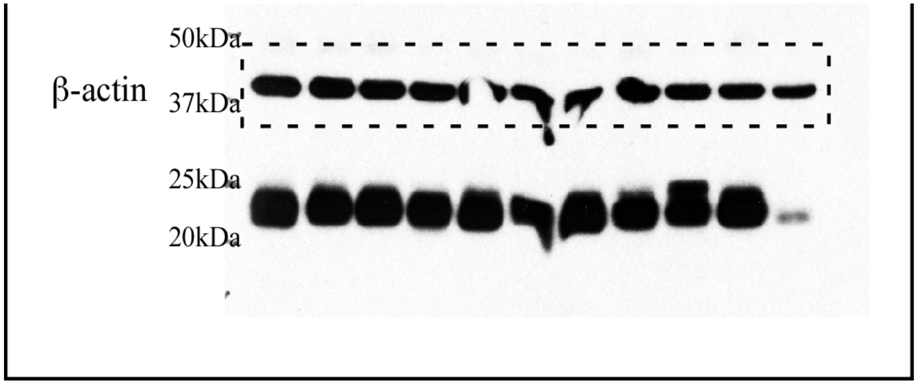

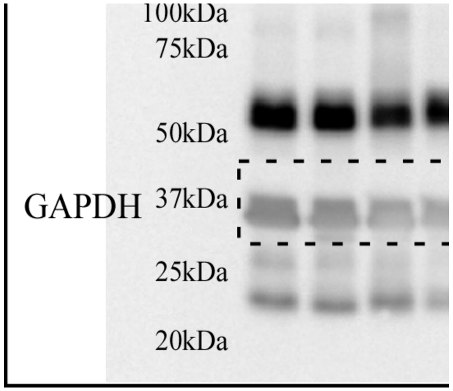

*Dotted area is what is shown in publication figure

Supplemental Fig 2b - APP and CTF western blots in the hippocampus

Antibdy: APP C-term, Sigma, A8717, lot037M4808V (1:2000)

APP at 4-week WO

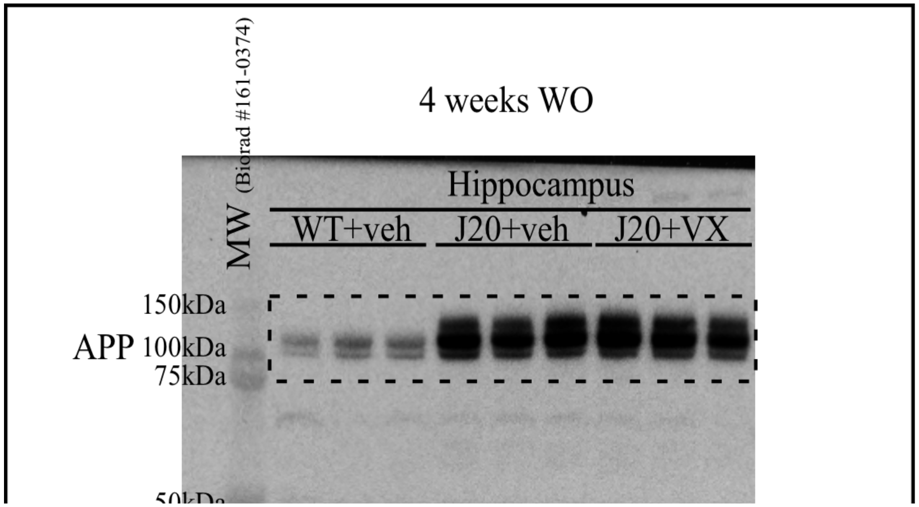

APP at 20-week WO

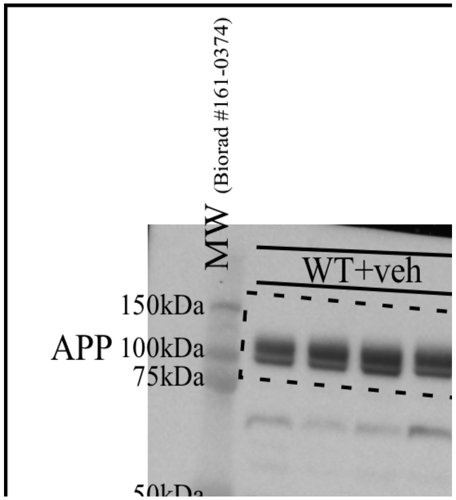

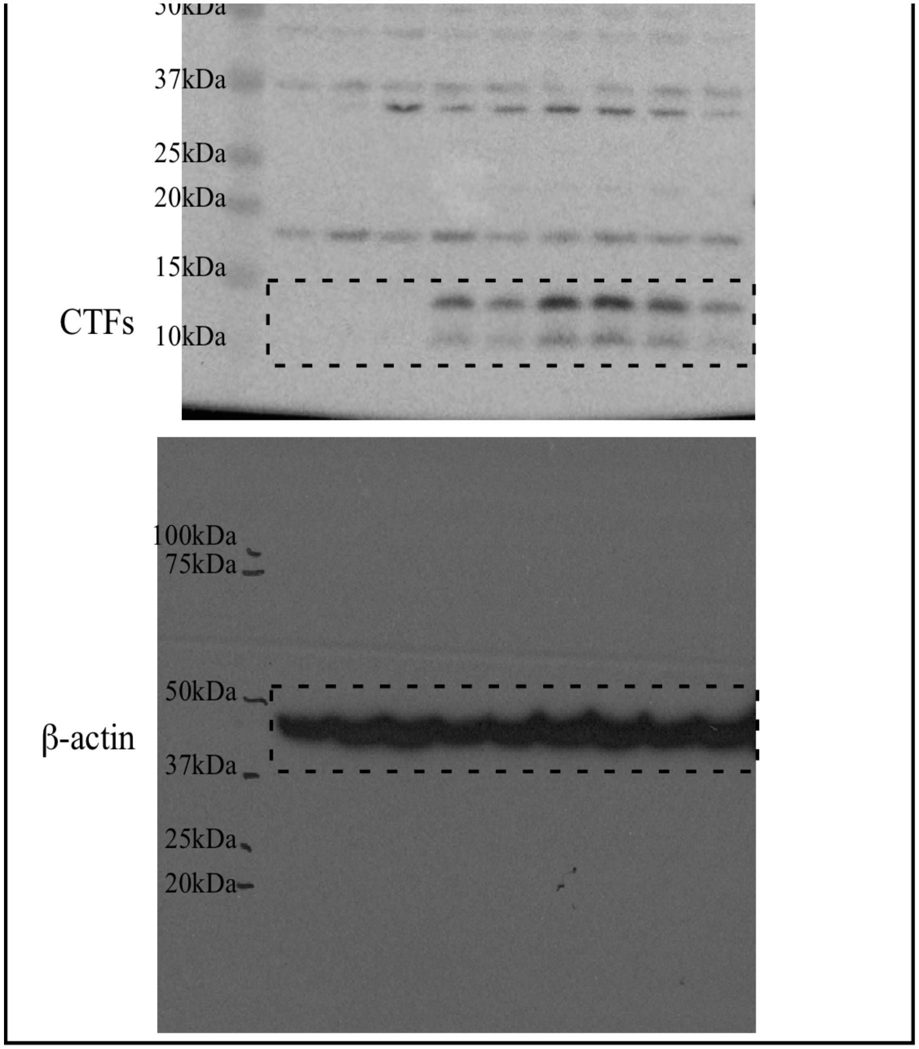

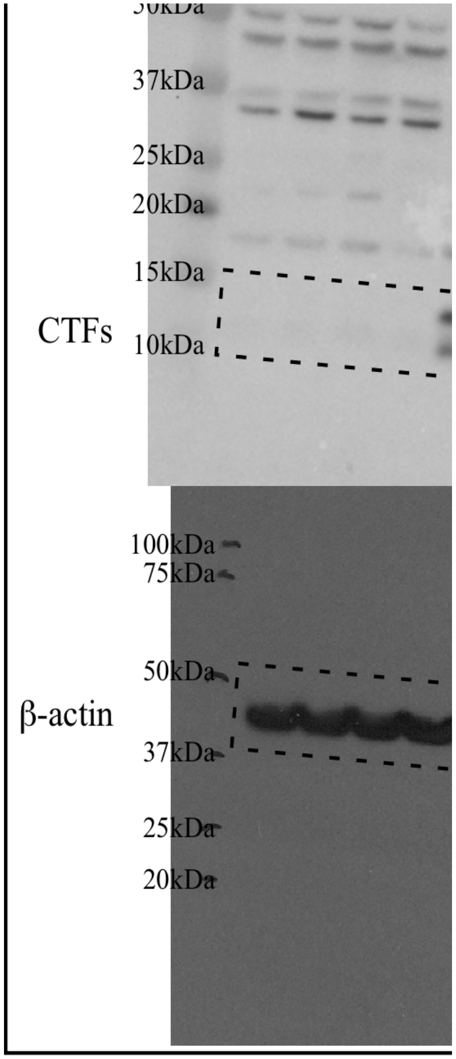

*Dotted area is what is shown in publication figure

Lung

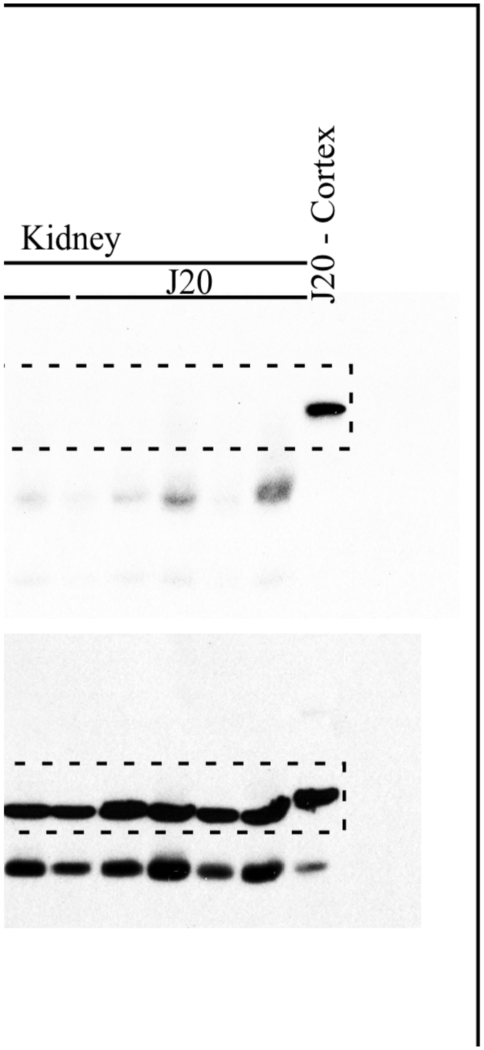
Kidney
J20
J20 - Cortex

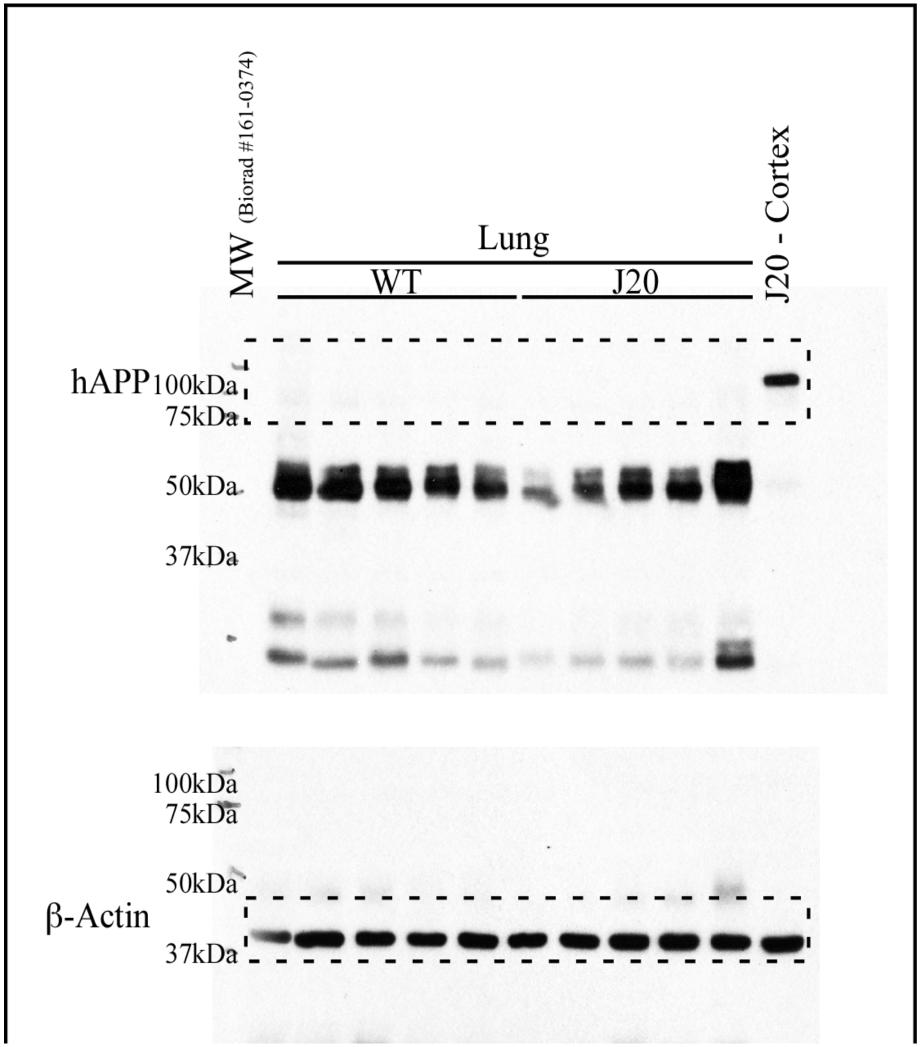
MW (Biorad #161-0374)
Lung
WT
J20
J20 - Cortex
hAPP
100kDa
75kDa
50kDa
37kDa
100kDa
75kDa
50kDa
β-Actin
37kDa

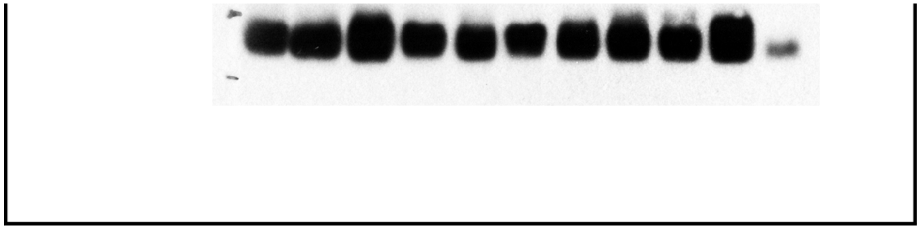

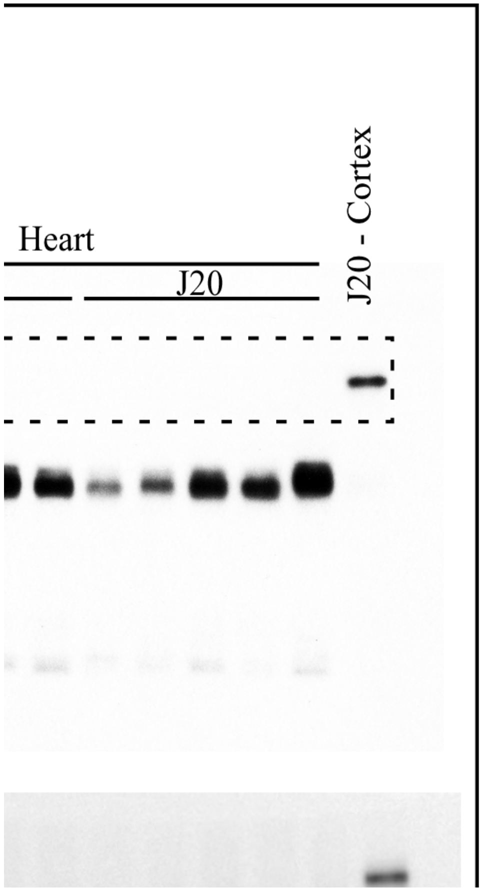
Heart
J20
J20 - Cortex

Muscle

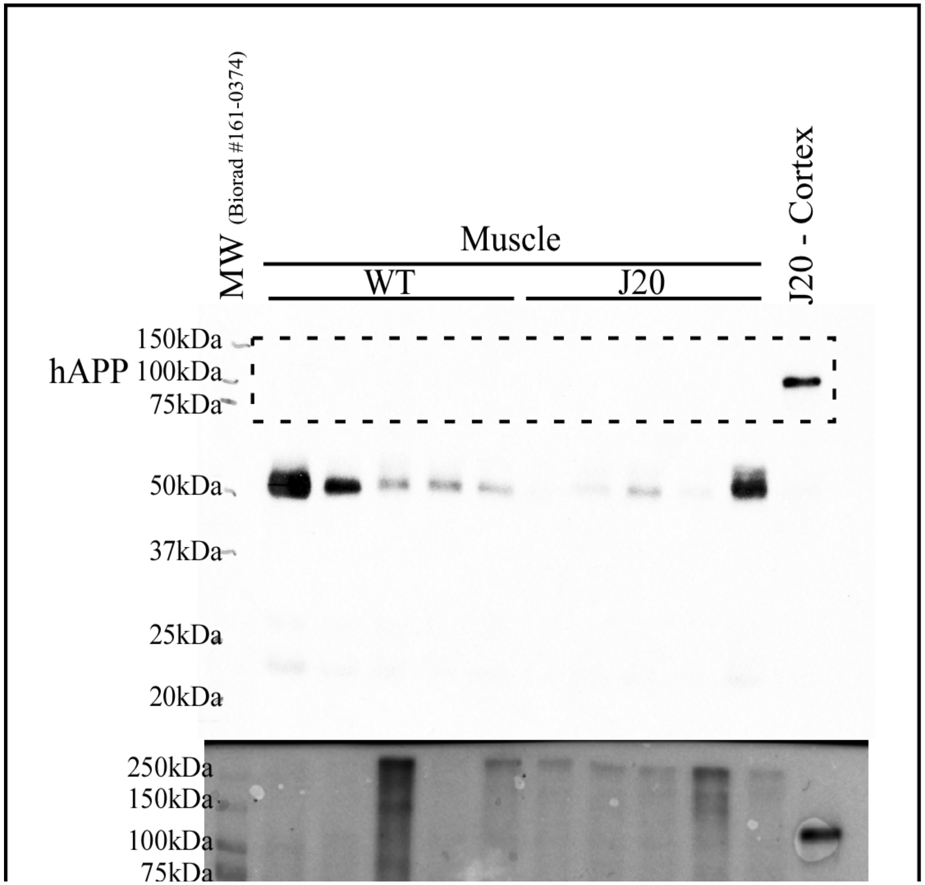
MW (Biorad #161-0374)
Muscle
WT
J20
J20 - Cortex
hAPP
150kDa
100kDa
75kDa
50kDa
37kDa
25kDa
20kDa
250kDa
150kDa
100kDa
75kDa

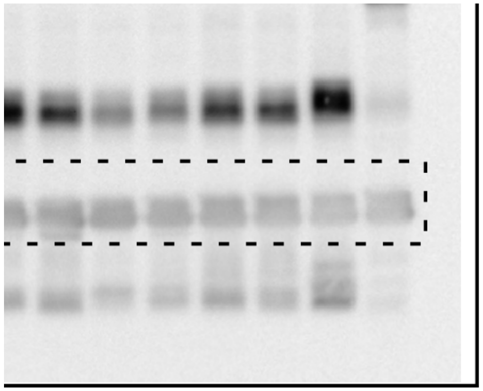

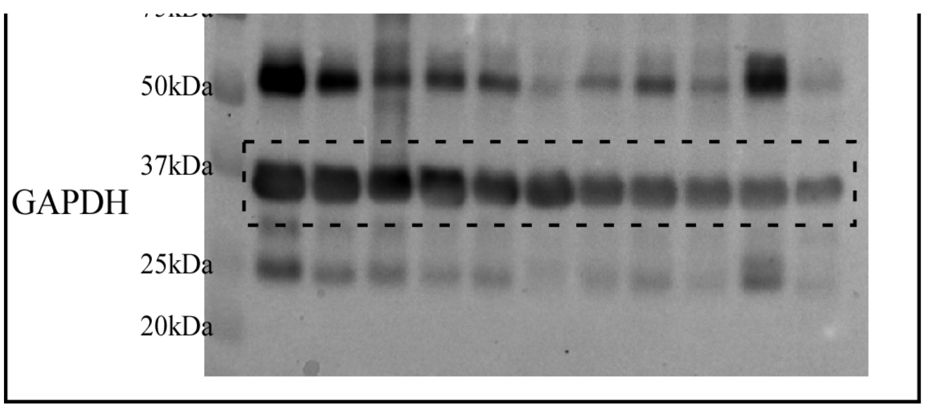

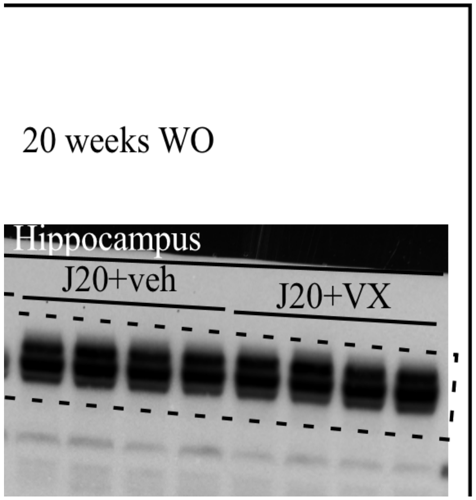

Supplemental Fig 2c-e - APP and CTF western blot quantification (% of J20

Hippo		4-week WO		
	WT + veh	J20+veh	J20+VX	WT+veh
full length APP	**21.4**	**113.2**	**98.0**	**39.4**
	**28.1**	**92.2**	**102.3**	**45.1**
	**29.2**	**94.6**	**88.9**	**52.5**
	**17.7**	**121.1**	**82.4**	**46.7**
	**26.6**	**124.1**	**91.8**	**18.0**
	**31.0**	**62.1**	**108.0**	**21.6**
	**29.5**	**92.7**	**98.1**	**17.6**
				**26.8**
β-CTF/APP	n/a	**90.0**	**130.3**	n/a
	n/a	**73.5**	**109.3**	n/a
	n/a	**136.5**	**64.8**	n/a
	n/a	**87.2**	**107.0**	n/a

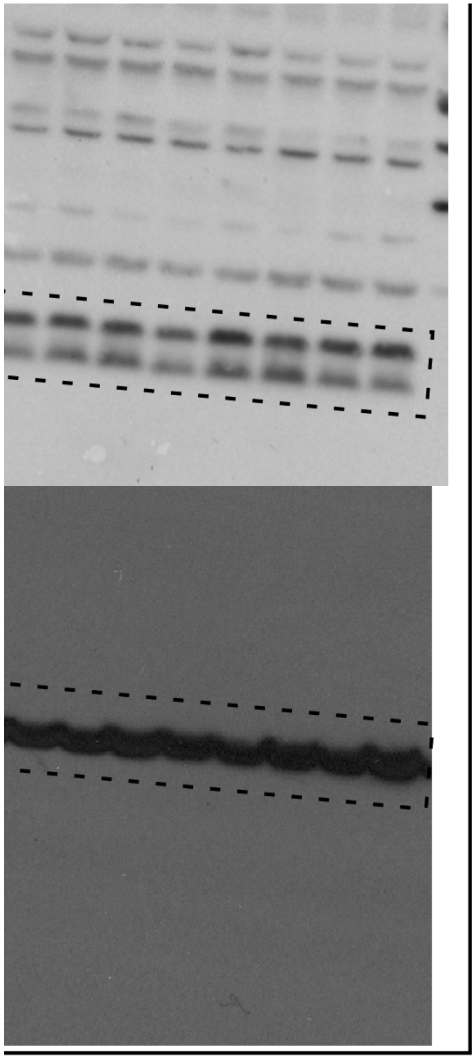

	n/a	**127.7**	**82.6**	n/a
	n/a	**85.0**	**115.1**	n/a
	n/a		**86.5**	n/a
α-CTF/APP	n/a	**86.8**	**145.5**	n/a
	n/a	**70.4**	**118.9**	n/a
	n/a	**142.8**	**61.6**	n/a
	n/a	**83.2**	**126.1**	n/a
	n/a	**120.7**	**87.9**	n/a
	n/a	**96.2**	**113.7**	n/a
	n/a		**86.4**	n/a

+ veh)

20-week WO

J20+veh	J20+VX
**108.0**	**105.7**
**100.7**	**93.5**
**95.8**	**92.8**
**95.5**	**80.3**
**96.9**	**115.4**
**105.8**	**94.2**
**97.3**	**109.8**
**101.4**	**133.8**
**101.2**	**110.6**
**109.4**	**116.9**
**87.9**	**124.1**

92.8	73.0
78.7	85.2
128.6	75.3
87.6	126.1
102.2	136.5
120.4	105.1
89.8	94.8
115.4	79.8
76.4	104.9
108.2	77.1

Supplemental Fig 3b - Iba1 quantification at 8- and 16-week WO

Hippo	Genotype	Treatment	TOTAL # Iba1	Total Volume Analyzed (mm3)	**CELL DENSITY (cells/mm3)**
8-week WO	WT	Vehicle	136	0.03422	**3973.8**
	WT	Vehicle	280	0.04563	**6136.0**
	WT	Vehicle	206	0.04563	**4514.4**
	J20	Vehicle	354	0.03125	**11328.7**
	J20	Vehicle	248	0.02083	**11904.8**
	J20	Vehicle	224	0.02282	**9817.7**
	J20	VX-765	302	0.03422	**8824.2**
	J20	VX-765	238	0.02282	**10431.3**
	J20	VX-765	183	0.02282	**8020.7**
16-week WO	WT	Vehicle	152	0.02480	**6129.0**
	WT	Vehicle	142	0.02778	**5112.3**
	WT	Vehicle	152	0.02678	**5675.0**
	J20	Vehicle	298	0.03472	**8582.9**
	J20	Vehicle	240	0.02579	**9305.2**
	J20	Vehicle	351	0.03770	**9311.3**
	J20	VX-765	271	0.04365	**6208.8**
	J20	VX-765	208	0.02778	**7488.5**
	J20	VX-765	264	0.05952	**4435.5**

Cortex	Genotype	Treatment	TOTAL # Iba1
8-week WO	WT	Vehicle	240
	WT	Vehicle	232
	WT	Vehicle	188
	J20	Vehicle	262
	J20	Vehicle	294
	J20	Vehicle	200
	J20	VX-765	180
	J20	VX-765	162
	J20	VX-765	240
16-week WO	WT	Vehicle	170
	WT	Vehicle	224
	WT	Vehicle	224
	J20	Vehicle	356
	J20	Vehicle	294
	J20	Vehicle	210
	J20	VX-765	148
	J20	VX-765	180
	J20	VX-765	154

Total Volume Analyzed (mm3)	**CELL DENSITY (cells/mm3)**
0.03968	**6048.4**
0.03968	**5846.8**
0.02976	**6317.2**
0.02083	**12576.8**
0.02083	**14112.9**
0.01984	**10080.6**
0.02282	**7889.2**
0.02282	**7100.3**
0.01984	**12096.8**
0.01984	**8568.5**
0.03968	**5645.2**
0.03968	**5645.2**
0.02976	**11962.4**
0.02976	**9879.0**
0.01984	**10584.7**
0.01984	**7459.7**
0.01984	**9072.6**
0.01984	**7762.1**

Supplemental Fig 5b - GFAP density quantification at 8- and 16-week WO

Hippo	Genotype	Treatment	SUM/Total Stained Area (um2)	SUM/Total Area Analyzed (converted to mm^2)	SUM Surface Area Staining (um^2 staining/mm^2)	Cortex	Genotype	Treatment	SUM/Total Stained Area (um2)
8-week WO	WT	vehicle	54182.0	2.260	**23973.1**	8-week WO	WT	vehicle	4504
	WT	vehicle	82102	2.260	**36326.4**		WT	vehicle	7467
	WT	vehicle	71020	2.260	**31423.1**		WT	vehicle	6876
	J20	vehicle	83592	2.260	**36985.7**		J20	vehicle	1991
	J20	vehicle	114054	2.260	**50463.7**		J20	vehicle	12850
	J20	vehicle	101909	2.260	**45090.1**		J20	vehicle	15568
	J20	VX-765	99821	2.260	**44166.3**		J20	VX-765	9459
	J20	VX-765	85633	2.260	**37888.7**		J20	VX-765	3700
	J20	VX-765	90720	2.260	**40139.5**		J20	VX-765	7411
16-week WO	WT	vehicle	57838	2.260	**25590.7**	16-week WO	WT	vehicle	3055
	WT	vehicle	74419	2.260	**32927.0**		WT	vehicle	5476
	WT	vehicle	70833	2.260	**31340.4**		WT	vehicle	5540
	J20	vehicle	130844	2.260	**57892.6**		J20	vehicle	16850
	J20	vehicle	102040	2.260	**45148.1**		J20	vehicle	16127
	J20	vehicle	122970	2.260	**54408.7**		J20	vehicle	17620
	J20	VX-765	93791	2.260	**41498.3**		J20	VX-765	10447
	J20	VX-765	119992	2.260	**53091.0**		J20	VX-765	29353
	J20	VX-765	109038	2.260	**48244.4**		J20	VX-765	12992

SUM/Total Area Analyzed (converted to mm^2)	**SUM Surface Area Staining (um^2 staining/mm^2)**
1.457	**3090.3**
1.457	**5123.3**
1.457	**4717.8**
1.457	**1366.1**
1.457	**8816.7**
1.457	**10681.6**
1.457	**6490.1**
1.457	**2538.7**
1.457	**5084.9**
1.457	**2096.1**
1.457	**3757.2**
1.457	**3801.1**
1.457	**11561.3**
1.457	**11065.2**
1.457	**12089.6**
1.457	**7168.0**
1.457	**20139.9**
1.457	**8914.2**

Supplemental Fig 6a - IL-1b and IL-18 qPCR at 8- and 16-week WO

				IL-1β hippo		
Genotype	Treatment	WO (Weeks)	Mean Ct IL-1β	Mean Ct HPRT+18s	ΔCt	$2^{-\Delta Ct}$
WT	Vehicle	8	28.4163208	15.28797054	13.12835026	**0.0001117**
WT	Vehicle	8	26.75408936	14.86557388	11.88851547	**0.0002638**
WT	Vehicle	8	28.60889816	15.546556	13.06234217	**0.0001169**
J20	Vehicle	8	27.87713814	15.26888847	12.60824966	**0.0001602**
J20	Vehicle	8	28.42812538	15.08697319	13.34115219	**0.0000964**
J20	Vehicle	8	26.42760658	15.06822872	11.35937786	**0.0003806**
J20	VX-765	8	29.80192947	15.41906929	14.38286018	**0.0000468**
J20	VX-765	8	28.58401871	15.31393862	13.27008009	**0.0001012**
J20	VX-765	8	26.00772858	15.37920666	10.62852192	**0.0006317**
J20	VX-765	8	26.59811211	15.08683872	11.51127338	**0.0003426**
WT	Vehicle	16	28.96328354	15.22953463	13.73374891	**0.0000734**
WT	Vehicle	16	27.83516693	15.27529335	12.55987358	**0.0001656**
WT	Vehicle	16	28.1128521	15.08020163	13.03265047	**0.0001193**
WT	Vehicle	16	28.83586502	15.46365452	13.3722105	**0.0000943**
J20	Vehicle	16	27.4910965	15.18231869	12.30877781	**0.0001971**
J20	Vehicle	16	27.76722717	15.49986172	12.26736546	**0.0002028**
J20	Vehicle	16	26.86598587	15.15642214	11.70956373	**0.0002986**
J20	Vehicle	16	26.30711365	14.93968391	11.36742973	**0.0003785**
J20	VX-765	16	28.36128998	14.99911451	13.36217546	**0.0000950**
J20	VX-765	16	28.64811707	15.05091619	13.59720087	**0.0000807**
J20	VX-765	16	28.4313755	15.29935551	13.13202	**0.0001114**
J20	VX-765	16	26.79457474	15.02690887	11.76766586	**0.0002868**

Supplemental Fig 6b - IL-1b ELISA at 8- and 16-week WO

					Hippo	
Genotype	Treatment	WO (weeks)	Calc. Concentration	Sample Volume (ml)	Total Amount (pg)	Protein Loaded (ug)
WT	vehicle	8	0.0128	0.025	0.0003	100
WT	vehicle	8	0.0000	0.025	0.0000	100

WT	vehicle	8	0.0261	0.025	0.0007	100
J20	vehicle	8	0.0521	0.025	0.0013	100
J20	vehicle	8	0.0000	0.025	0.0000	100
J20	vehicle	8	0.0000	0.025	0.0000	100
J20	VX-765	8	0.0169	0.025	0.0004	100
J20	VX-765	8	0.0000	0.025	0.0000	100
J20	VX-765	8	0.0000	0.025	0.0000	100
WT	vehicle	16	0.0000	0.025	0.0000	100
WT	vehicle	16	0.2113	0.025	0.0053	100
WT	vehicle	16	0.0615	0.025	0.0015	100
J20	vehicle	16	0.0000	0.025	0.0000	100
J20	vehicle	16	0.0568	0.025	0.0014	100
J20	vehicle	16	0.1066	0.025	0.0027	100
J20	VX-765	16	0.1900	0.025	0.0048	100
J20	VX-765	16	0.9295	0.025	0.0232	100
J20	VX-765	16	0.4418	0.025	0.0110	100

Supplemental Fig 6c - Nlrp1, Nlrp3, Asc qPCR

				**Nlrp1 hippo**		
Genotype	Treatment	WO (Weeks)	Mean Ct Nlrp1	Mean Ct HPRT+18s	ΔCt	**$2^{-\Delta Ct}$**
WT	Vehicle	4	27.4077301	15.09490585	12.31282425	**0.000196549**
WT	Vehicle	4	27.45674133	15.37163448	12.08510685	**0.000230155**
WT	Vehicle	4	27.2324295	15.34946823	11.88296127	**0.000264772**
WT	Vehicle	4	26.90886116	15.17251396	11.7363472	**0.000293094**
J20	Vehicle	4	27.02495193	15.37090969	11.65404224	**0.000310301**
J20	Vehicle	4	27.24744415	15.28404999	11.96339417	**0.000250415**
J20	Vehicle	4	27.34807968	14.81592607	12.53215361	**0.000168829**
J20	VX-765	4	28.46483994	15.5962553	12.86858463	**0.000133712**
J20	VX-765	4	26.96561813	15.2533884	11.71222973	**0.000298035**
J20	VX-765	4	27.14540863	15.36834908	11.77705956	**0.000284939**
WT	Vehicle	8	27.20701027	15.28797054	11.91903973	**0.000258233**

WT	Vehicle	8	27.36463928	14.86557388	12.4990654	**0.000172745**
WT	Vehicle	8	27.42549896	15.546556	11.87894297	**0.000265511**
J20	Vehicle	8	27.13435173	15.26888847	11.86546326	**0.000268003**
J20	Vehicle	8	26.75969315	15.08697319	11.67271996	**0.00030631**
J20	Vehicle	8	26.82810402	15.06822872	11.7598753	**0.000288353**
J20	VX-765	8	28.40525436	15.08683872	13.31841564	**9.78943E-05**
J20	VX-765	8	27.17992783	15.41906929	11.76085854	**0.000288157**
J20	VX-765	8	26.77294731	15.31393862	11.45900869	**0.000355218**
J20	VX-765	8	27.75315475	15.37920666	12.3739481	**0.000188395**
WT	Vehicle	12	26.84168625	15.26136637	11.58031988	**0.000326570**
WT	Vehicle	12	27.26680565	14.97976685	12.2870388	**0.000200093**
WT	Vehicle	12	26.52207947	15.04685688	11.47522259	**0.000351248**
WT	Vehicle	12	27.45344162	15.26461792	12.1888237	**0.000214190**
J20	Vehicle	12	27.44779968	15.21114445	12.23665524	**0.000207205**
J20	Vehicle	12	26.72609711	15.20016241	11.5259347	**0.000339116**
J20	Vehicle	12	27.41921043	14.65073061	12.76847982	**0.000143319**
J20	VX-765	12	27.03635597	15.46437502	11.57198095	**0.000328463**
J20	VX-765	12	26.79122162	15.16145897	11.62976265	**0.000315568**
J20	VX-765	12	26.52927017	15.32771206	11.20155811	**0.000424615**
WT	Vehicle	16	27.01091003	15.22953463	11.78137541	**0.000284088**
WT	Vehicle	16	27.09383774	15.27529335	11.81854439	**0.000276862**
WT	Vehicle	16	26.91384888	15.08020163	11.83364725	**0.000273979**
WT	Vehicle	16	27.19090271	15.46365452	11.72724819	**0.000294949**
J20	Vehicle	16	27.03002167	15.18231869	11.84770298	**0.000271323**
J20	Vehicle	16	26.45425797	15.49986172	10.95439625	**0.000503962**
J20	Vehicle	16	27.27188492	15.15642214	12.11546278	**0.000225363**
J20	Vehicle	16	26.17500305	14.93968391	11.23531914	**0.000414793**
J20	VX-765	16	28.80858994	15.02690887	13.78168106	**7.10069E-05**
J20	VX-765	16	26.61354065	14.99911451	11.61442614	**0.000318940**
J20	VX-765	16	26.56291389	15.05091619	11.5119977	**0.000342408**
J20	VX-765	16	26.64473724	15.29935551	11.34538174	**0.000384326**

WT	Vehicle	20	27.53358459	15.10013771	12.43344688	**0.000180784**
WT	Vehicle	20	27.15975571	15.26441288	11.89534283	**0.000262509**
WT	Vehicle	20	26.81150818	15.46088028	11.3506279	**0.000382931**
WT	Vehicle	20	27.52961731	15.31291771	12.2166996	**0.000210091**
J20	Vehicle	20	26.0605011	15.21928406	10.84121704	**0.000545090**
J20	Vehicle	20	27.10487175	15.23942089	11.86545086	**0.000268005**
J20	Vehicle	20	26.84225845	15.38573456	11.4565239	**0.000355830**
J20	Vehicle	20	26.89677048	15.03895283	11.85781765	**0.000269427**
J20	VX-765	20	27.74499893	15.5668292	12.17816973	**0.000215777**
J20	VX-765	20	27.51121521	15.04809523	12.46311998	**0.000177103**
J20	VX-765	20	26.95484924	15.19388771	11.76096153	**0.000288136**
J20	VX-765	20	26.94906235	15.09176922	11.85729313	**0.000269525**

Supplemental Fig 6d - Casp1 & Casp6 qPCR at 8- and 16-week WO

				**Casp1 hippo**		
Genotype	Treatment	WO (Weeks)	Mean Ct Casp1	Mean Ct HPRT+18s	ΔCt	$2^{-\Delta Ct}$
WT	Vehicle	8	25.40096283	15.28797054	10.11299229	**0.000902996**
WT	Vehicle	8	23.75003052	14.86557388	8.884456635	**0.002115982**
WT	Vehicle	8	26.39551163	15.546556	10.84895563	**0.000542174**
J20	Vehicle	8	25.13505173	15.26888847	9.866163254	**0.001071492**
J20	Vehicle	8	25.40407181	15.08697319	10.31709862	**0.000783870**
J20	Vehicle	8	23.57518959	15.06822872	8.506960869	**0.002748841**
J20	VX-765	8	25.64000511	15.41906929	10.22093582	**0.000837899**
J20	VX-765	8	24.43170929	15.31393862	9.117770672	**0.001800021**
J20	VX-765	8	23.48386765	15.37920666	8.104660988	**0.003632904**
J20	VX-765	8	23.67964172	15.08683872	8.592803001	**0.002590052**
WT	Vehicle	16	25.71224594	15.22953463	10.48271132	**0.000698859**
WT	Vehicle	16	23.10977173	15.27529335	7.834478378	**0.004381139**
WT	Vehicle	16	24.02802277	15.08020163	8.94782114	**0.002025058**
WT	Vehicle	16	25.6531868	15.46365452	10.18953228	**0.000856338**
J20	Vehicle	16	25.38663483	15.18231869	10.20431614	**0.000847607**

J20	Vehicle	16	24.90952873	15.49986172	9.409667015	**0.001470307**
J20	Vehicle	16	23.86299706	15.15642214	8.706574917	**0.002393645**
J20	Vehicle	16	22.85697746	14.93968391	7.917293549	**0.004136730**
J20	VX-765	16	24.95008659	14.99911451	9.95097208	**0.001010320**
J20	VX-765	16	25.10087585	15.05091619	10.04995966	**0.000943324**
J20	VX-765	16	25.23459244	15.29935551	9.935236931	**0.001021400**
J20	VX-765	16	23.63962746	15.02690887	8.612718582	**0.002554544**

Supplemental Fig 6e - Casp11 & Casp3 qPCR

				**Casp11 hippo**		
Genotype	Treatment	WO (Weeks)	Mean Ct Casp11	Mean Ct HPRT+18s	ΔCt	**$2^{-\Delta Ct}$**
WT	Vehicle	4	29.26938248	15.09490585	14.17447662	**5.40826E-05**
WT	Vehicle	4	27.3228569	15.37163448	11.95122242	**0.000252536**
WT	Vehicle	4	28.12957382	15.34946823	12.78010559	**0.000142169**
WT	Vehicle	4	27.59666824	15.17251396	12.42415428	**0.000181952**
J20	Vehicle	4	28.88598251	15.37090969	13.51507282	**8.54196E-05**
J20	Vehicle	4	27.04553223	15.28404999	11.76148224	**0.000288032**
J20	Vehicle	4	26.00058746	14.81592607	11.18466139	**0.000429617**
J20	VX-765	4	27.53212547	15.5962553	11.93587017	**0.000255238**
J20	VX-765	4	27.70116806	15.2533884	12.44777966	**0.000178997**
J20	VX-765	4	26.97019958	15.36834908	11.60185051	**0.000321733**
WT	Vehicle	8	26.92164803	15.28797054	11.63367748	**0.000314713**
WT	Vehicle	8	27.85887146	14.86557388	12.99329758	**0.000122639**
WT	Vehicle	8	26.38492584	15.546556	10.83836985	**0.000546167**
J20	Vehicle	8	26.71808243	15.26888847	11.44919395	**0.000357643**
J20	Vehicle	8	25.92523956	15.08697319	10.83826637	**0.000546206**
J20	Vehicle	8	25.98620224	15.06822872	10.91797352	**0.000516848**
J20	VX-765	8	26.06992149	15.08683872	10.98308277	**0.000494041**
J20	VX-765	8	28.00460815	15.41906929	12.58553886	**0.000162695**
J20	VX-765	8	26.68567848	15.31393862	11.37173986	**0.000377368**
J20	VX-765	8	26.80728912	15.37920666	11.42808247	**0.000362915**

WT	Vehicle	12	26.60956573	15.26136637	11.34819937	**0.000383576**
WT	Vehicle	12	27.54809761	14.97976685	12.56833076	**0.000164648**
WT	Vehicle	12	26.38115692	15.04685688	11.33430004	**0.000387289**
WT	Vehicle	12	26.82666206	15.26461792	11.56204414	**0.000330733**
J20	Vehicle	12	28.66493988	15.21114445	13.45379543	**8.91259E-05**
J20	Vehicle	12	27.06260681	15.20016241	11.8624444	**0.000268564**
J20	Vehicle	12	26.04178429	14.65073061	11.39105368	**0.000372350**
J20	VX-765	12	28.19555092	15.46437502	12.7311759	**0.000147073**
J20	VX-765	12	26.05050278	15.16145897	10.88904381	**0.000527316**
J20	VX-765	12	25.65250206	15.32771206	10.32479	**0.000779702**
WT	Vehicle	16	28.21355438	15.22953463	12.98401976	**0.00012343**
WT	Vehicle	16	26.96628761	15.27529335	11.69099426	**0.00030245**
WT	Vehicle	16	26.82403755	15.08020163	11.74383593	**0.00029158**
WT	Vehicle	16	28.01860046	15.46365452	12.55494595	**0.00016618**
J20	Vehicle	16	28.26598549	15.18231869	13.0836668	**0.00011519**
J20	Vehicle	16	26.12729645	15.49986172	10.62743473	**0.00063215**
J20	Vehicle	16	25.84825134	15.15642214	10.6918292	**0.00060456**
J20	Vehicle	16	24.92639923	14.93968391	9.986715317	**0.00098560**
J20	VX-765	16	25.6074028	15.02690887	10.58049393	**0.00065306**
J20	VX-765	16	26.83963013	14.99911451	11.84051561	**0.00027268**
J20	VX-765	16	26.57058716	15.05091619	11.51967096	**0.00034059**
J20	VX-765	16	26.25193024	15.29935551	10.95257473	**0.00050460**
WT	Vehicle	20	26.88454819	15.10013771	11.78441048	**0.00028349**
WT	Vehicle	20	27.99816895	15.26441288	12.73375607	**0.00014681**
WT	Vehicle	20	27.89271545	15.46088028	12.43183517	**0.00018099**
WT	Vehicle	20	27.37532425	15.31291771	12.06240654	**0.00023381**
J20	Vehicle	20	25.88990974	15.21928406	10.67062569	**0.00061351**
J20	Vehicle	20	26.65482712	15.23942089	11.41540623	**0.00036612**
J20	Vehicle	20	28.38585281	15.38573456	13.00011826	**0.00012206**
J20	Vehicle	20	25.49596024	15.03895283	10.45700741	**0.00071142**
J20	VX-765	20	26.14715195	15.5668292	10.58032274	**0.00065314**

J20	VX-765	20	27.30503845	15.04809523	12.25694323	**0.00020431**
J20	VX-765	20	27.65109253	15.19388771	12.45720482	**0.00017783**
J20	VX-765	20	26.93715286	15.09176922	11.84538364	**0.00027176**

Supplemental Fig 6f - Casp1 western blot in the cortex

Supplemental Fi

Antibody: Casp1 Genentech, clone 4B4.2.1 (1:1000)

Cortex

Casp1 at 20-week WO

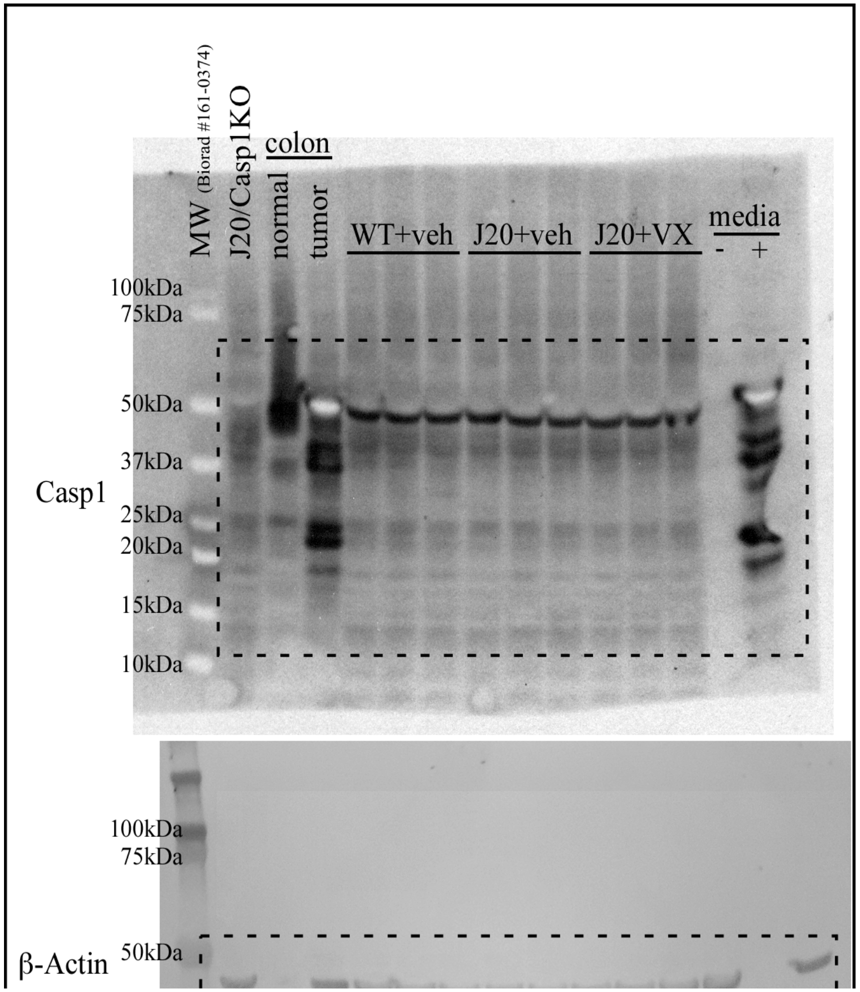

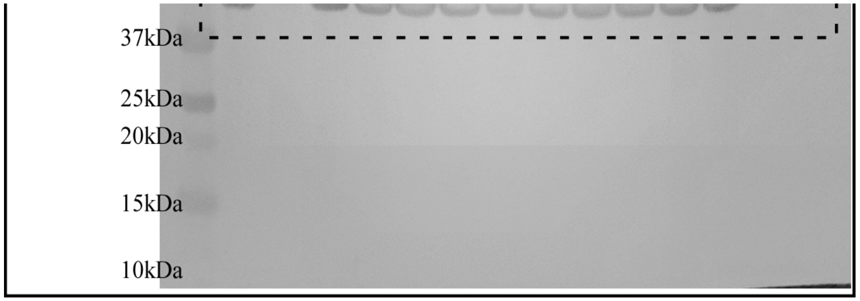

*Dotted area is what is shown in publication figure

				IL-18 hippo	
Genotype	Treatment	WO (Weeks)	Mean Ct IL-18	Mean Ct HPRT+18s	ΔCt
WT	Vehicle	8	19.92592621	15.28797054	4.637955666
WT	Vehicle	8	19.83255768	14.86557388	4.966983795
WT	Vehicle	8	20.46709633	15.546556	4.920540333
J20	Vehicle	8	19.94356346	15.26888847	4.674674988
J20	Vehicle	8	19.92775345	15.08697319	4.840780258
J20	Vehicle	8	19.43536377	15.06822872	4.367135048
J20	VX-765	8	20.47327042	15.41906929	5.054201126
J20	VX-765	8	20.16731262	15.31393862	4.853374004
J20	VX-765	8	19.86621284	15.37920666	4.487006187
J20	VX-765	8	19.37147522	15.08683872	4.284636497
WT	Vehicle	16	20.31093597	15.22953463	5.081401348
WT	Vehicle	16	19.78676605	15.27529335	4.511472702
WT	Vehicle	16	19.61853981	15.08020163	4.538338184
WT	Vehicle	16	20.47813797	15.46365452	5.014483452
J20	Vehicle	16	20.51074219	15.18231869	5.3284235
J20	Vehicle	16	20.23106003	15.49986172	4.731198311
J20	Vehicle	16	19.50312996	15.15642214	4.346707821
J20	Vehicle	16	19.3657608	14.93968391	4.426076889
J20	VX-765	16	20.08013725	14.99911451	5.081022739
J20	VX-765	16	19.77641869	15.05091619	4.725502491
J20	VX-765	16	20.39958572	15.29935551	5.100230217
J20	VX-765	16	19.34169388	15.02690887	4.314785004

**Final [conc] pg/mg**	Genotype	Treatment	WO (weeks)	Calc. Concentration	Sample Volume (ml)
**0.0032**	WT	vehicle	8	0.3966	0.025
**0.0000**	WT	vehicle	8	0.3534	0.025

**0.0065**	WT	vehicle	8	0.7286	0.025
**0.0130**	J20	vehicle	8	0.2333	0.025
**0.0000**	J20	vehicle	8	0.2033	0.025
**0.0000**	J20	vehicle	8	0.2233	0.025
**0.0042**	J20	VX-765	8	0.2760	0.025
**0.0000**	J20	VX-765	8	0.2418	0.025
**0.0000**	J20	VX-765	8	0.2548	0.025
**0.0000**	WT	vehicle	16	0.3837	0.025
**0.0528**	WT	vehicle	16	0.3620	0.025
**0.0154**	WT	vehicle	16	0.3103	0.025
**0.0000**	J20	vehicle	16	0.2674	0.025
**0.0142**	J20	vehicle	16	0.2589	0.025
**0.0267**	J20	vehicle	16	0.2503	0.025
**0.0475**	J20	VX-765	16	0.3017	0.025
**0.2324**	J20	VX-765	16	0.7022	0.025
**0.1105**	J20	VX-765	16	0.4399	0.025

Nlrp3 hippo

Mean Ct Nlrp3	Mean Ct HPRT+18s	ΔCt	$2^{-\Delta Ct}$	Mean Ct Asc
27.63767624	15.09490585	12.54277039	**0.000167591**	22.84293365
26.7940464	15.37163448	11.42241192	**0.000364344**	22.93770599
27.51598358	15.34946823	12.16651535	**0.000217527**	22.82434082
27.10115051	15.17251396	11.92863655	**0.000256521**	22.88940811
27.50805283	15.37090969	12.13714314	**0.000222001**	23.36496353
27.47592354	15.28404999	12.19187355	**0.000213737**	22.72709656
26.544384	14.81592607	11.72845793	**0.000294702**	22.43763733
27.75839615	15.5962553	12.16214085	**0.000218188**	23.49879837
26.66058731	15.2533884	11.40719891	**0.000368206**	22.49999809
26.62106514	15.36834908	11.25271606	**0.000409822**	22.46400452
26.27550888	15.28797054	10.98753834	**0.000492517**	22.72053719

28.07448196	14.86557388	13.20890808	**0.000105614**	22.93718338
27.12336731	15.546556	11.57681131	**0.000327365**	22.66900635
26.89203644	15.26888847	11.62314796	**0.000317018**	22.81354141
27.10165405	15.08697319	12.01468086	**0.000241669**	22.63045883
27.32245636	15.06822872	12.25422764	**0.000204696**	22.97602844
26.3721199	15.08683872	11.28528118	**0.000400675**	22.68986511
26.47071457	15.41906929	11.05164528	**0.000471111**	23.070858
26.99278641	15.31393862	11.67884779	**0.000305012**	22.07646751
27.0235672	15.37920666	11.64436054	**0.000312391**	22.89517593
26.78751373	15.26136637	11.52614737	**0.000339066**	22.53740692
27.22459412	14.97976685	12.24482727	**0.000206034**	22.78757858
27.23253632	15.04685688	12.18567944	**0.000214657**	22.6307354
26.91518784	15.26461792	11.65056992	**0.000311049**	23.19001389
27.74005699	15.21114445	12.52891254	**0.000169208**	23.54799271
26.7490387	15.20016241	11.54887629	**0.000333766**	22.72566795
26.2586174	14.65073061	11.60788679	**0.000320389**	22.4219799
27.47960472	15.46437502	12.0152297	**0.000241577**	22.79030609
27.40396118	15.16145897	12.24250221	**0.000206367**	22.57663727
26.47682381	15.32771206	11.14911175	**0.000440335**	21.93437195
26.85950851	15.22953463	11.62997389	**0.000315522**	23.10404587
27.68315315	15.27529335	12.4078598	**0.000184019**	22.790205
26.6716156	15.08020163	11.59141397	**0.000324068**	23.16490746
26.18686867	15.46365452	10.72321415	**0.000591549**	23.07961273
26.85370255	15.18231869	11.67138386	**0.000306594**	22.76075935
26.33197975	15.49986172	10.83211803	**0.000548539**	22.47505188
26.58490753	15.15642214	11.42848539	**0.000362813**	22.66278076
26.11110115	14.93968391	11.17141724	**0.000433579**	22.05827713
26.78466034	15.02690887	11.75775146	**0.000288778**	22.67975807
26.59373474	14.99911451	11.59462023	**0.000323349**	21.98915482
25.36767197	15.05091619	10.31675577	**0.000784056**	22.16104126
27.68932724	15.29935551	12.38997173	**0.000186315**	22.36182785

26.79719162	15.10013771	11.69705391	**0.000301187**		23.09697342
27.04623795	15.26441288	11.78182507	**0.000283999**		23.40105438
26.88305283	15.46088028	11.42217255	**0.000364404**		22.93511581
27.33500481	15.31291771	12.0220871	**0.000240431**		22.74847603
26.08164406	15.21928406	10.86236	**0.00053716**		21.96502495
26.82861137	15.23942089	11.58919048	**0.000324568**		22.4370842
27.29893303	15.38573456	11.91319847	**0.000259281**		23.19024086
26.89095688	15.03895283	11.85200405	**0.000270515**		22.52828598
26.37100601	15.5668292	10.80417681	**0.000559266**		23.00663376
27.50966454	15.04809523	12.46156931	**0.000177294**		22.93099976
26.53657532	15.19388771	11.34268761	**0.000385044**		22.44746017
26.79325104	15.09176922	11.70148182	**0.000300264**		22.55303192

				**Casp6 hippo**	
Genotype	Treatment	WO (Weeks)	Mean Ct Casp6	Mean Ct HPRT+18s	ΔCt
WT	Vehicle	8	23.37458038	15.28797054	8.08660984
WT	Vehicle	8	24.44509888	14.86557388	9.579524994
WT	Vehicle	8	23.79133034	15.546556	8.244774342
J20	Vehicle	8	23.32868004	15.26888847	8.059791565
J20	Vehicle	8	23.35689163	15.08697319	8.269918442
J20	Vehicle	8	23.0310421	15.06822872	7.962813377
J20	VX-765	8	22.90459633	15.08683872	7.817757607
J20	VX-765	8	23.63541794	15.41906929	8.216348648
J20	VX-765	8	23.84668541	15.31393862	8.532746792
J20	VX-765	8	23.82261658	15.37920666	8.44340992
WT	Vehicle	16	23.61372566	15.22953463	8.384191036
WT	Vehicle	16	23.84242249	15.27529335	8.567129135
WT	Vehicle	16	23.40550232	15.08020163	8.325300694
WT	Vehicle	16	23.33737373	15.46365452	7.873719215
J20	Vehicle	16	23.72105217	15.18231869	8.538733482

J20	Vehicle	16	23.47115517	15.49986172	7.971293449
J20	Vehicle	16	22.58581543	15.15642214	7.429393291
J20	Vehicle	16	22.59422302	14.93968391	7.654539108
J20	VX-765	16	22.60874939	15.02690887	7.581840515
J20	VX-765	16	23.08358765	14.99911451	8.084473133
J20	VX-765	16	23.6020546	15.05091619	8.551138401
J20	VX-765	16	23.57836914	15.29935551	8.279013634

Casp3 hippo

Mean Ct Casp3	Mean Ct HPRT+18s	ΔCt	$2^{-\Delta Ct}$
22.73989677	15.09490585	7.644990921	**0.004996069**
22.64310646	15.37163448	7.271471977	**0.006472452**
22.68971252	15.34946823	7.340244293	**0.006171153**
22.50415421	15.17251396	7.331640244	**0.006208067**
23.2991066	15.37090969	7.928196907	**0.004105584**
23.08381844	15.28404999	7.799768448	**0.004487823**
22.40679359	14.81592607	7.590867519	**0.005187059**
22.90016937	15.5962553	7.30391407	**0.006328529**
22.81847763	15.2533884	7.565089226	**0.005280575**
23.04213905	15.36834908	7.673789978	**0.004897326**
22.74172592	15.28797054	7.453755379	**0.005704217**
22.27225113	14.86557388	7.406677246	**0.005893428**
23.13497543	15.546556	7.588419437	**0.005195868**
22.53513718	15.26888847	7.266248703	**0.006495928**
23.05193329	15.08697319	7.964960098	**0.004002286**
22.49115372	15.06822872	7.422924995	**0.005827428**
21.92786026	15.08683872	6.841021538	**0.008722627**
23.24098015	15.41906929	7.821910858	**0.00441947**
23.18051529	15.31393862	7.866576672	**0.00428474**
22.57581711	15.37920666	7.196610451	**0.006817174**

22.90105247	15.26136637	7.639686108	**0.005014473**
22.51504326	14.97976685	7.535276413	**0.005390832**
22.81139946	15.04685688	7.76454258	**0.00459875**
22.67281342	15.26461792	7.408195496	**0.005887229**
22.82294655	15.21114445	7.611802101	**0.005112334**
22.51634979	15.20016241	7.316187382	**0.006274919**
22.06519699	14.65073061	7.414466381	**0.005861695**
23.06503868	15.46437502	7.600663662	**0.005151957**
22.34378815	15.16145897	7.182329178	**0.006884993**
22.59399414	15.32771206	7.266282082	**0.006495777**
22.72661018	15.22953463	7.497075558	**0.005535481**
22.58847427	15.27529335	7.313180923	**0.006288009**
22.78510284	15.08020163	7.704901218	**0.004792848**
23.27782059	15.46365452	7.814166069	**0.004443259**
22.92553711	15.18231869	7.743218422	**0.004667228**
22.70039368	15.49986172	7.20053196	**0.006798669**
22.3425827	15.15642214	7.186160564	**0.006866732**
21.89985275	14.93968391	6.960168839	**0.008031199**
22.25708771	15.02690887	7.230178833	**0.006660384**
22.71070862	14.99911451	7.711594105	**0.004770664**
22.78669548	15.05091619	7.735779285	**0.004691356**
23.24143219	15.29935551	7.942076683	**0.004066274**
22.80275726	15.10013771	7.702619553	**0.004800434**
22.8489151	15.26441288	7.58450222	**0.005209995**
23.02573776	15.46088028	7.564857483	**0.005281423**
21.90865135	15.31291771	6.595733643	**0.010339186**
22.85563278	15.21928406	7.636348724	**0.005026087**
23.09091568	15.23942089	7.851494789	**0.004329767**
23.32556152	15.38573456	7.939826965	**0.00407262**
21.81421471	15.03895283	6.775261879	**0.009129415**
22.30913925	15.5668292	6.742310047	**0.009340335**

22.55715942	15.04809523	7.509064198	**0.005489673**
22.94172287	15.19388771	7.747835159	**0.004652316**
22.68410492	15.09176922	7.592335701	**0.005181783**

g 6g - Casp1 western blot quantification (% of WT + veh)

	20-week WO	
WT + veh	J20+veh	J20+VX
**75.5**	**139.9**	**98.5**
**96.7**	**111.4**	**92.0**
**127.8**	**100.3**	**63.1**

$2^{-\Delta Ct}$
**0.0401639**
**0.0319734**
**0.0330194**
**0.0391546**
**0.0348963**
**0.0484575**
**0.0300977**
**0.0345930**
**0.0445940**
**0.0513093**
**0.0295356**
**0.0438441**
**0.0430352**
**0.0309378**
**0.0248877**
**0.0376502**
**0.0491485**
**0.0465177**
**0.0295434**
**0.0377991**
**0.0291526**
**0.0502482**

Cortex

Total Amount (pg)	Protein Loaded (ug)	**Final [conc] pg/mg**
0.0099	100	**0.0991**
0.0088	100	**0.0883**

0.0182	100	**0.1822**
0.0058	100	**0.0583**
0.0051	100	**0.0508**
0.0056	100	**0.0558**
0.0069	100	**0.0690**
0.0060	100	**0.0604**
0.0064	100	**0.0637**
0.0096	100	**0.0959**
0.0090	100	**0.0905**
0.0078	100	**0.0776**
0.0067	100	**0.0669**
0.0065	100	**0.0647**
0.0063	100	**0.0626**
0.0075	100	**0.0754**
0.0176	100	**0.1756**
0.0110	100	**0.1100**

Asc hippo

Mean Ct HPRT+18s	ΔCt	$2^{-\Delta Ct}$
15.09490585	7.748027802	**0.004651695**
15.37163448	7.56607151	**0.005276981**
15.34946823	7.474872589	**0.005621331**
15.17251396	7.71689415	**0.00475317**
15.37090969	7.994053841	**0.003922383**
15.28404999	7.44304657	**0.005746716**
14.81592607	7.621711254	**0.005077341**
15.5962553	7.902543068	**0.004179242**
15.2533884	7.246609688	**0.00658496**
15.36834908	7.095655441	**0.007311305**
15.28797054	7.432566643	**0.005788613**

14.86557388	8.071609497	**0.003717093**
15.546556	7.122450352	**0.007176766**
15.26888847	7.544652939	**0.005355909**
15.08697319	7.543485641	**0.005360244**
15.06822872	7.907799721	**0.004164042**
15.08683872	7.60302639	**0.005143527**
15.41906929	7.651788712	**0.004972583**
15.31393862	6.762528896	**0.009210346**
15.37920666	7.515969276	**0.00546346**
15.26136637	7.276040554	**0.006451988**
14.97976685	7.807811737	**0.004462872**
15.04685688	7.583878517	**0.005212248**
15.26461792	7.925395966	**0.004113562**
15.21114445	8.336848259	**0.003092848**
15.20016241	7.525505543	**0.005427466**
14.65073061	7.771249294	**0.004577421**
15.46437502	7.325931072	**0.006232682**
15.16145897	7.415178299	**0.005858803**
15.32771206	6.606659889	**0.010261178**
15.22953463	7.874511242	**0.004261239**
15.27529335	7.514911652	**0.005467467**
15.08020163	8.08470583	**0.003683503**
15.46365452	7.615958214	**0.005097628**
15.18231869	7.578440666	**0.005231931**
15.49986172	6.975190163	**0.007948012**
15.15642214	7.506358624	**0.005499977**
14.93968391	7.118593216	**0.007195979**
15.02690887	7.652849197	**0.00496893**
14.99911451	6.990040302	**0.00786662**
15.05091619	7.110125065	**0.007238342**
15.29935551	7.062472343	**0.007481419**

15.10013771	7.996835709	**0.003914827**
15.26441288	8.136641502	**0.003553259**
15.46088028	7.474235535	**0.005623813**
15.31291771	7.435558319	**0.005776621**
15.21928406	6.745740891	**0.009318149**
15.23942089	7.197663307	**0.006812201**
15.38573456	7.804506302	**0.004473109**
15.03895283	7.489333153	**0.005565268**
15.5668292	7.439804554	**0.005759644**
15.04809523	7.88290453	**0.00423652**
15.19388771	7.253572464	**0.006553256**
15.09176922	7.461262703	**0.005674611**

$2^{-\Delta Ct}$
**0.00367864**
**0.00130700**
**0.00329667**
**0.00374767**
**0.00323971**
**0.00400825**
**0.00443221**
**0.00336227**
**0.00270015**
**0.00287263**
**0.00299300**
**0.00263656**
**0.00311770**
**0.00426358**
**0.00268896**

0.00398475
0.00580136
0.00496311
0.00521962
0.00368410
0.00266594
0.00321935

Supplemental Fig 7b - Aβ staning density at 8- and 16-week WO

**Hippo**	Genotype	Treatment	SUM/Total Stained Area (um2)	SUM/Total Area Analyzed (converted to mm^2)	**SUM Surface Area Staining (um^2 staining/mm^2)**	**Cortex**	Genotype
8-week WO	J20	vehicle	16056	2.5561	**6281.4**	8-week WO	J20
	J20	vehicle	8752	2.5561	**3424.0**		J20
	J20	vehicle	8752	2.5561	**3424.0**		J20
	J20	VX-765	4135	2.5561	**1617.7**		J20
	J20	VX-765	4264	1.9171	**2224.2**		J20
	J20	VX-765	2519	1.2781	**1971.0**		J20
16-week WO	J20	vehicle	30482	2.5561	**11925.1**	16-week WO	J20
	J20	vehicle	40064	2.5561	**15673.8**		J20
	J20	vehicle	38101	2.5561	**14905.8**		J20
	J20	vehicle	121533	5.1122	**23773.0**		J20
	J20	VX-765	9162	1.2781	**7168.7**		J20
	J20	VX-765	39254	2.5561	**15356.9**		J20
	J20	VX-765	8343	1.2781	**6527.9**		J20

Treatment	SUM/Total Stained Area (um2)	SUM/Total Area Analyzed (converted to mm^2)	**SUM Surface Area Staining (um^2 staining/mm^2)**
vehicle	1333	5.1122	**260.7**
vehicle	473	1.2781	**370.1**
vehicle	470	1.2781	**367.7**
VX-765	1001	2.5561	**391.6**
VX-765	499	1.2781	**390.4**
VX-765	492	1.2781	**385.0**
vehicle	8362	2.5561	**3271.4**
vehicle	11509	2.5561	**4502.5**
vehicle	6719	2.5561	**2628.6**
vehicle	49208	5.1122	**9625.6**
VX-765	10520	2.5561	**4115.6**
VX-765	4048	1.2781	**3167.3**
VX-765	4612	1.2781	**3608.6**

Supplemental Fig 8a - Total Aβ RIPA levels and Aβ42/total Aβ ratio at 8- and 16-week WO

			RIPA			
**Hippo**		Treatment	**Total Aβ (pg/mg)**	**Aβ42/Total Aβ**	**Cortex**	
8-week WO	J20	vehicle	**258.35**	**0.11**	8-week WO	J20
	J20	vehicle	**250.67**	**0.11**		J20
	J20	vehicle	**253.97**	**0.11**		J20
	J20	VX-765	**258.67**	**0.10**		J20
	J20	VX-765	**245.44**	**0.11**		J20
	J20	VX-765	**251.17**	**0.10**		J20
16-week WO	J20	vehicle	**312.22**	**0.13**	16-week WO	J20
	J20	vehicle	**252.84**	**0.13**		J20
	J20	vehicle	**364.32**	**0.16**		J20
	J20	VX-765	**0.00**	**0.00**		J20
	J20	VX-765	**284.03**	**0.12**		J20
	J20	VX-765	**321.06**	**0.17**		J20

Supplemental Fig 8b - Aβ RIPA ELISA levels

					**Aβ38**	
**Hippo**		Treatment	Brain Region	Calc. Conc (pg)	Sample (ul)	Concentration (pg/ml)
4-Week Washout	J20	vehicle	Hippo	1051.2769	6.25	6.5705
	J20	vehicle	Hippo	1038.3870	6.25	6.4899
	J20	vehicle	Hippo	1029.4971	6.25	6.4344
	J20	vehicle	Hippo	214.0359	14.00	2.9965
	J20	vehicle	Hippo	194.3206	14.00	2.7205
	J20	vehicle	Hippo	251.1013	14.00	3.5154
	J20	vehicle	Hippo	207.5586	14.00	2.9058
	J20	VX-765	Hippo	1057.6621	6.25	6.6104
	J20	VX-765	Hippo	1046.5917	6.25	6.5412
	J20	VX-765	Hippo	1050.5630	6.25	6.5660
	J20	VX-765	Hippo	235.0655	14.00	3.2909
	J20	VX-765	Hippo	213.0671	14.00	2.9829

	J20	VX-765	Hippo	208.0813	14.00	2.9131
	J20	VX-765	Hippo	104.6096	14.00	1.4645
	J20	VX-765	Hippo	202.5810	14.00	2.8361
8-Week Washout	J20	VX-765	Hippo	840.0601	6.25	5.2504
	J20	VX-765	Hippo	803.9442	6.25	5.0247
	J20	VX-765	Hippo	818.0022	6.25	5.1125
	J20	vehicle	Hippo	809.4596	6.25	5.0591
	J20	vehicle	Hippo	799.0989	6.25	4.9944
	J20	vehicle	Hippo	800.2793	6.25	5.0017
12-Week Washout	J20	vehicle	Hippo	917.8487	6.25	5.7366
	J20	vehicle	Hippo	864.7105	6.25	5.4044
	J20	vehicle	Hippo	890.4000	6.25	5.5650
	J20	vehicle	Hippo	246.8183	14.00	3.4555
	J20	vehicle	Hippo	279.8686	14.00	3.9182
	J20	vehicle	Hippo	272.1943	14.00	3.8107
	J20	VX-765	Hippo	1227.1398	6.25	7.6696
	J20	VX-765	Hippo	1245.1226	6.25	7.7820
	J20	VX-765	Hippo	1236.2523	6.25	7.7266
	J20	VX-765	Hippo	258.6547	14.00	3.6212
	J20	VX-765	Hippo	308.9737	14.00	4.3256
	J20	VX-765	Hippo	142.5273	14.00	1.9954
	J20	VX-765	Hippo	131.0330	14.00	1.8345
16-Week Washout	J20	vehicle	Hippo	1067.8054	6.25	6.6738
	J20	vehicle	Hippo	768.4734	6.25	4.8030
	J20	vehicle	Hippo	1189.2222	6.25	7.4326
	J20	VX-765	Hippo	0.0000	6.25	0.0000
	J20	VX-765	Hippo	1056.5000	6.25	6.6031
	J20	VX-765	Hippo	1003.4281	6.25	6.2714
20-Week Washout	J20	vehicle	Hippo	605.5500	6.25	3.7847

J20	vehicle	Hippo	768.6267	6.25	4.8039
J20	vehicle	Hippo	934.9215	6.25	5.8433
J20	vehicle	Hippo	1057.4962	6.25	6.6094
J20	vehicle	Hippo	1030.0895	6.25	6.4381
J20	vehicle	Hippo	273.9145	14.00	3.8348
J20	vehicle	Hippo	219.9020	14.00	3.0786
J20	vehicle	Hippo	263.5281	14.00	3.6894
J20	VX-765	Hippo	1113.8766	6.25	6.9617
J20	VX-765	Hippo	1117.2587	6.25	6.9829
J20	VX-765	Hippo	1260.8569	6.25	7.8804
J20	VX-765	Hippo	1170.5378	6.25	7.3159
J20	VX-765	Hippo	210.8425	14.00	2.9518
J20	VX-765	Hippo	222.2537	14.00	3.1116
J20	VX-765	Hippo	189.1426	14.00	2.6480

Supplemental Fig 8c - Total Aβ FA levels and Aβ42/total Aβ ratio at 8- and 16-week WO

			Hippo FA			
**Hippo**		Treatment	**Total Aβ (pg/mg)**	**Aβ42/Total Aβ**	**Cortex**	
8-week WO	J20	vehicle	**1353.13**	**0.28**	8-week WO	J20
	J20	vehicle	**1304.27**	**0.28**		J20
	J20	VX-765	**1314.88**	**0.25**		J20
	J20	VX-765	**1266.79**	**0.25**		J20
16-week WO	J20	vehicle	**9189.71**	**0.27**	16-week WO	J20
	J20	vehicle	**19010.07**	**0.80**		J20
	J20	vehicle	**27619.90**	**0.33**		J20
	J20	VX-765	**10503.79**	**0.32**		J20
	J20	VX-765	**21390.32**	**0.08**		J20
	J20	VX-765	**20376.36**	**0.31**		J20

Supplemental Fig 8d - Aβ FA ELISA levels

Hippo		Treatment	Brain Region	Aβ38 (pg/mg)	Aβ40 (pg/mg)
4-week WO	J20	Vehicle	Hippo	**188.3705**	**228.1645**
	J20	Vehicle	Hippo	**210.4211**	**209.2246**
	J20	Vehicle	Hippo	**0.0000**	**84.8840**
	J20	Vehicle	Hippo	**0.0000**	**245.4889**
	J20	Vehicle	Hippo	**0.0000**	**206.8570**
	J20	Vehicle	Hippo	**0.0000**	**168.0162**
	J20	VX-765	Hippo	**167.2738**	**201.8665**
	J20	VX-765	Hippo	**191.6155**	**252.5324**
	J20	VX-765	Hippo	**0.0000**	**142.1422**
	J20	VX-765	Hippo	**0.0000**	**121.6261**
	J20	VX-765	Hippo	**0.0000**	**96.2096**
	J20	VX-765	Hippo	**0.0000**	**66.8183**
	J20	VX-765	Hippo	**0.0000**	**364.1865**
8-week WO	J20	Vehicle	Hippo	**368.8056**	**601.0783**
	J20	Vehicle	Hippo	**405.9228**	**532.5019**
	J20	VX-765	Hippo	**409.3302**	**573.7061**
	J20	VX-765	Hippo	**402.5153**	**541.7486**
12-week WO	J20	Vehicle	Hippo	**1047.9332**	**1344.2812**
	J20	Vehicle	Hippo	**1085.4062**	**1534.6552**
	J20	Vehicle	Hippo	**2254.8483**	**6058.6797**
	J20	Vehicle	Hippo	**2392.1620**	**5606.9017**
	J20	VX-765	Hippo	**932.6378**	**1260.1948**
	J20	VX-765	Hippo	**1001.3265**	**1373.1887**
	J20	VX-765	Hippo	**2036.3673**	**6321.1135**
	J20	VX-765	Hippo	**5665.9243**	**12403.9052**
	J20	VX-765	Hippo	**107.2664**	**538.9222**
16-week WO	J20	Vehicle	Hippo	**2660.6907**	**4003.9903**
	J20	Vehicle	Hippo	**1486.0464**	**2295.3961**

	J20	Vehicle	Hippo	**7997.2030**	**10569.1515**
	J20	VX-765	Hippo	**3014.5477**	**4133.4860**
	J20	VX-765	Hippo	**8838.0006**	**10873.0377**
	J20	VX-765	Hippo	**5687.2025**	**8469.0616**
20-week WO	J20	Vehicle	Hippo	**5255.7355**	**8188.6735**
	J20	Vehicle	Hippo	**4215.8959**	**5564.4598**
	J20	Vehicle	Hippo	**3462.1837**	**5385.2472**
	J20	Vehicle	Hippo	**3932.6997**	**5784.6461**
	J20	Vehicle	Hippo	**1558.1980**	**3988.6635**
	J20	Vehicle	Hippo	**3183.1470**	**10103.9538**
	J20	Vehicle	Hippo	**2611.0531**	**7337.3792**
	J20	VX-765	Hippo	**5059.1599**	**7724.0106**
	J20	VX-765	Hippo	**5483.7981**	**7212.3918**
	J20	VX-765	Hippo	**6653.4339**	**9603.0745**
	J20	VX-765	Hippo	**6707.3575**	**9445.3691**
	J20	VX-765	Hippo	**8730.3148**	**18385.1221**
	J20	VX-765	Hippo	**2315.3923**	**8214.3818**
	J20	VX-765	Hippo	**7139.6169**	**18002.9869**

	RIPA	
Treatment	**Total Aβ (pg/mg)**	**Aβ42/Total Aβ**
vehicle	**143.59**	**0.11**
vehicle	**138.72**	**0.11**
vehicle	**132.75**	**0.12**
VX-765	**123.44**	**0.11**
VX-765	**114.04**	**0.12**
VX-765	**112.73**	**0.11**
vehicle	**176.48**	**0.10**
vehicle	**119.57**	**0.13**
vehicle	**154.38**	**0.15**
VX-765	**0.00**	**0.00**
VX-765	**142.04**	**0.12**
VX-765	**126.61**	**0.13**

	**Aβ40**				**Aβ**	
**FINAL Conc (pg/mg)**	Calc. Conc (pg)	Sample (ul)	Concentration (pg/ml)	**FINAL Conc (pg/mg)**	Calc. Conc (pg)	Sample (ul)
**105.1277**	1828.4322	6.25	11.4277	**182.8432**	315.5585	6.25
**103.8387**	1861.5276	6.25	11.6345	**186.1528**	314.8765	6.25
**102.9497**	1897.4392	6.25	11.8590	**189.7439**	311.6710	6.25
**107.0179**	454.5767	14.00	6.3641	**227.2883**	105.3309	14
**97.1603**	430.6792	14.00	6.0295	**215.3396**	90.5524	14
**125.5507**	581.6705	14.00	8.1434	**290.8353**	90.6972	14
**103.7793**	423.8281	14.00	5.9336	**211.9141**	86.9808	14
**105.7662**	1881.7028	6.25	11.7606	**188.1703**	311.8041	6.25
**104.6592**	1877.6833	6.25	11.7355	**187.7683**	301.5938	6.25
**105.0563**	1727.6641	6.25	10.7979	**172.7664**	293.3385	6.25
**117.5327**	513.0385	14.00	7.1825	**256.5192**	87.7470	14
**106.5336**	487.0281	14.00	6.8184	**243.5140**	87.8340	14

**104.0407**	434.2949	14.00	6.0801	**217.1474**	81.8825	14
**52.3048**	318.9886	14.00	4.4658	**159.4943**	67.3767	14
**101.2905**	425.9836	14.00	5.9638	**212.9918**	83.7389	14
**84.0060**	1450.3087	6.25	9.0644	**145.0309**	293.1677	6.25
**80.3944**	1418.8770	6.25	8.8680	**141.8877**	283.8938	6.25
**81.8002**	1433.4554	6.25	8.9591	**143.3455**	288.2728	6.25
**80.9460**	1509.3294	6.25	9.4333	**150.9329**	267.9484	6.25
**79.9099**	1391.4292	6.25	8.6964	**139.1429**	263.9167	6.25
**80.0279**	1450.3913	6.25	9.0649	**145.0391**	261.0720	6.25
**91.7849**	1635.1908	6.25	10.2199	**163.5191**	372.4399	6.25
**86.4710**	1786.7406	6.25	11.1671	**178.6741**	380.8579	6.25
**89.0400**	1700.8174	6.25	10.6301	**170.0817**	373.5804	6.25
**123.4091**	535.9532	14.00	7.5033	**267.9766**	113.5193	14
**139.9343**	529.6963	14.00	7.4157	**264.8481**	142.0424	14
**136.0972**	548.3353	14.00	7.6767	**274.1677**	121.3375	14
**122.7140**	1971.7849	6.25	12.3237	**197.1785**	316.9531	6.25
**124.5123**	1973.5943	6.25	12.3350	**197.3594**	320.9062	6.25
**123.6252**	1972.7653	6.25	12.3298	**197.2765**	317.8764	6.25
**129.3274**	632.2254	14.00	8.8512	**316.1127**	131.9705	14
**154.4869**	652.1793	14.00	9.1305	**326.0897**	134.3601	14
**71.2636**	350.0306	14.00	4.9004	**175.0153**	95.6764	14
**65.5165**	325.2866	14.00	4.5540	**162.6433**	71.5609	14
**106.7805**	1658.8382	6.25	10.3677	**165.8838**	395.5475	6.25
**76.8473**	1420.2081	6.25	8.8763	**142.0208**	339.6727	6.25
**118.9222**	1886.1175	6.25	11.7882	**188.6118**	567.8415	6.25
**0.0000**	0.0000	6.25	0.0000	**0.0000**	0.0000	6.25
**105.6500**	1454.4115	6.25	9.0901	**145.4411**	329.4069	6.25
**100.3428**	1666.2914	6.25	10.4143	**166.6291**	540.8777	6.25
**60.5550**	1108.2597	6.25	6.9266	**110.8260**	435.3469	6.25

**76.8627**	1059.1208	6.25	6.6195	**105.9121**	437.2276	6.25
**93.4922**	1413.9905	6.25	8.8374	**141.3991**	571.2077	6.25
**105.7496**	1509.5224	6.25	9.4345	**150.9522**	412.9404	6.25
**103.0090**	1697.8473	6.25	10.6115	**169.7847**	580.3796	6.25
**136.9573**	654.7687	14.00	9.1668	**327.3843**	352.3791	14
**109.9510**	474.2611	14.00	6.6397	**237.1305**	382.7510	14
**131.7640**	610.7906	14.00	8.5511	**305.3953**	206.8260	14
**111.3877**	1930.5329	6.25	12.0658	**193.0533**	483.2104	6.25
**111.7259**	1840.9667	6.25	11.5060	**184.0967**	480.1852	6.25
**126.0857**	1853.9993	6.25	11.5875	**185.3999**	430.9335	6.25
**117.0538**	1877.6833	6.25	11.7355	**187.7683**	453.9631	6.25
**105.4212**	502.7754	14.00	7.0389	**251.3877**	198.6317	14
**111.1268**	512.9413	14.00	7.1812	**256.4706**	191.4194	14
**94.5713**	494.6475	14.00	6.9251	**247.3238**	136.3820	14

	Cortex FA	
Treatment	**Total Aβ (pg/mg)**	**Aβ42/Total Aβ**
vehicle	**207.53**	**0.32**
vehicle	**189.87**	**0.38**
VX-765	**71.66**	**0.24**
VX-765	**56.23**	**0.26**

vehicle	**2032.90**	**0.69**
vehicle	**6968.53**	**0.58**
vehicle	**551.89**	**0.18**
VX-765	**2702.91**	**0.53**
VX-765	**701.86**	**0.29**
VX-765	**835.83**	**0.22**

Aβ42 (pg/mg)	Cortex		Treatment	Brain Region
**56.3953**	4-week WO	J20	Vehicle	Cortex
**53.6941**		J20	Vehicle	Cortex
**57.2507**		J20	Vehicle	Cortex
**286.9931**		J20	Vehicle	Cortex
**213.7694**		J20	Vehicle	Cortex
**79.1924**		J20	Vehicle	Cortex
**61.1725**		J20	VX-765	Cortex
**177.7222**		J20	VX-765	Cortex
**63.5315**		J20	VX-765	Cortex
**118.7140**		J20	VX-765	Cortex
**47.2749**		J20	VX-765	Cortex
**49.7689**		J20	VX-765	Cortex
**212.0453**		J20	VX-765	Cortex
**383.2414**	8-week WO	J20	Vehicle	Cortex
**365.8418**		J20	Vehicle	Cortex
**331.8440**		J20	VX-765	Cortex
**322.5236**		J20	VX-765	Cortex
**1060.8839**	12-week WO	J20	Vehicle	Cortex
**1056.8107**		J20	Vehicle	Cortex
**9580.1180**		J20	Vehicle	Cortex
**7077.7336**		J20	Vehicle	Cortex
**997.7075**		J20	VX-765	Cortex
**1025.4788**		J20	VX-765	Cortex
**7435.9601**		J20	VX-765	Cortex
**47085.8729**		J20	VX-765	Cortex
**453.8541**		J20	VX-765	Cortex
**2525.0256**	16-week WO	J20	Vehicle	Cortex
**15228.6324**		J20	Vehicle	Cortex

**9053.5477**		J20	Vehicle	Cortex
**3355.7567**		J20	VX-765	Cortex
**1679.2838**		J20	VX-765	Cortex
**6220.0918**		J20	VX-765	Cortex
**7982.5067**	20-week WO	J20	Vehicle	Cortex
**5002.9147**		J20	Vehicle	Cortex
**5204.1872**		J20	Vehicle	Cortex
**6167.8372**		J20	Vehicle	Cortex
**2720.3263**		J20	Vehicle	Cortex
**49270.1084**		J20	Vehicle	Cortex
**5043.2137**		J20	Vehicle	Cortex
**6552.6082**		J20	VX-765	Cortex
**7290.5979**		J20	VX-765	Cortex
**8141.9067**		J20	VX-765	Cortex
**8676.2704**		J20	VX-765	Cortex
**48000.0000**		J20	VX-765	Cortex
**5388.8589**		J20	VX-765	Cortex
**47500.0000**		J20	VX-765	Cortex

42

Concentration (pg/ml)	**FINAL Conc (pg/mg)**	**Cortex**	Treatment	Brain Region	Calc. Conc (pg)
1.9722	**31.5559**	4-Week Washout	Vehicle	Cortex	429.6264
1.9680	**31.4877**		Vehicle	Cortex	429.4281
1.9479	**31.1671**		Vehicle	Cortex	455.4704
1.4746	**52.6654**		Vehicle	Cortex	0.0000
1.2677	**45.2762**		Vehicle	Cortex	21.2106
1.2698	**45.3486**		Vehicle	Cortex	0.0000
1.2177	**43.4904**		Vehicle	Cortex	42.3862
1.9488	**31.1804**		VX-765	Cortex	578.2744
1.8850	**30.1594**		VX-765	Cortex	397.0285
1.8334	**29.3339**		VX-765	Cortex	398.6316
1.2285	**43.8735**		VX-765	Cortex	24.6598
1.2297	**43.9170**		VX-765	Cortex	34.9639

1.1464	**40.9412**		VX-765	Cortex	131.6459
0.9433	**33.6883**		VX-765	Cortex	97.1326
1.1723	**41.8694**		VX-765	Cortex	0.0000
1.8323	**29.3168**	8-Week Washout	VX-765	Cortex	430.4477
1.7743	**28.3894**		VX-765	Cortex	430.1214
1.8017	**28.8273**		VX-765	Cortex	411.8569
1.6747	**26.7948**		Vehicle	Cortex	360.6209
1.6495	**26.3917**		Vehicle	Cortex	360.2516
1.6317	**26.1072**		Vehicle	Cortex	359.4823
2.3277	**37.2440**	12-Week Washout	Vehicle	Cortex	446.5629
2.3804	**38.0858**		Vehicle	Cortex	408.2595
2.3349	**37.3580**		Vehicle	Cortex	408.7076
1.5893	**56.7597**		Vehicle	Cortex	0.0000
1.9886	**71.0212**		Vehicle	Cortex	46.4866
1.6987	**60.6687**		Vehicle	Cortex	35.5234
1.9810	**31.6953**		VX-765	Cortex	595.9283
2.0057	**32.0906**		VX-765	Cortex	612.8707
1.9867	**31.7876**		VX-765	Cortex	629.4673
1.8476	**65.9853**		VX-765	Cortex	65.7956
1.8810	**67.1801**		VX-765	Cortex	77.5526
1.3395	**47.8382**		VX-765	Cortex	31.9525
1.0019	**35.7804**		VX-765	Cortex	0.0000
2.4722	**39.5547**	16-Week Washout	Vehicle	Cortex	591.2914
2.1230	**33.9673**		Vehicle	Cortex	405.5432
3.5490	**56.7842**		Vehicle	Cortex	445.0535
0.0000	**0.0000**		VX-765	Cortex	0.0000
2.0588	**32.9407**		VX-765	Cortex	442.5113
3.3805	**54.0878**		VX-765	Cortex	402.9648
2.7209	**43.5347**	20-Week Washout	Vehicle	Cortex	511.7678

2.7327	**43.7228**	Vehicle	Cortex	469.4855
3.5700	**57.1208**	Vehicle	Cortex	524.0801
2.5809	**41.2940**	Vehicle	Cortex	479.5146
3.6274	**58.0380**	Vehicle	Cortex	479.5146
4.9333	**176.1896**	Vehicle	Cortex	92.4513
5.3585	**191.3755**	Vehicle	Cortex	43.7726
2.8956	**103.4130**	Vehicle	Cortex	76.0706
3.0201	**48.3210**	VX-765	Cortex	523.2173
3.0012	**48.0185**	VX-765	Cortex	536.6463
2.6933	**43.0933**	VX-765	Cortex	629.4673
2.8373	**45.3963**	VX-765	Cortex	610.7937
2.7808	**99.3158**	VX-765	Cortex	129.4068
2.6799	**95.7097**	VX-765	Cortex	57.9352
1.9093	**68.1910**	VX-765	Cortex	0.0000

Aβ38 (pg/mg)	Aβ40 (pg/mg)	Aβ42 (pg/mg)
0.0000	94.0963	32.1395
0.0000	106.1323	35.8661
0.0000	46.6241	19.1357
0.0000	27.7507	19.4233
0.0000	63.8107	15.6331
0.0000	23.8581	10.9516
0.0000	12.0811	5.1689
0.0000	43.4170	17.4593
0.0000	84.1823	40.1415
0.0000	41.3146	20.9226
0.0000	47.7004	12.1203
0.0000	53.0628	57.0402
0.0000	41.3146	7.6008
26.2607	114.1335	67.1346
0.0000	118.1335	71.7399
0.0000	54.3668	17.2964
0.0000	41.8370	14.3979
30.8287	227.3926	311.4142
0.0000	235.8084	367.6823
0.0000	169.3074	366.6075
228.0998	1039.2695	2131.5112
0.0000	157.0780	210.8750
0.0000	185.8802	279.4314
0.0000	58.8419	29.9697
0.0000	185.2499	213.0248
457.2993	1927.9244	2825.5767
105.9926	533.3291	1393.5761
959.7899	1981.4633	4027.2805

191.6157	258.6275	101.6451
494.7675	783.5325	1424.6148
110.8046	388.5943	202.4620
165.9768	487.4958	182.3565
359.6609	857.4647	662.8774
38.1027	334.0735	564.0530
160.0798	344.3493	379.7315
91.2554	100.1769	10.8371
378.2178	1396.8820	1651.3994
138.7256	1040.8581	1650.6005
128.7383	414.2935	333.3890
372.0279	399.5237	12.0249
413.8312	425.3189	14.6677
244.0634	302.1906	7.5803
236.5059	297.5312	9.0630
585.3715	2971.6138	5050.6625
374.1010	1410.8920	3353.3264
0.0000	192.5378	465.2674

Aβ38				Aβ40		
Sample (ul)	Concentration (pg/ml)	**FINAL Conc (pg/mg)**	Calc. Conc (pg)	Sample (ul)	Concentration (pg/ml)	**FINAL Conc (pg/mg)**
6.25	2.6852	**42.9626**	900.6347	6.25	5.6290	**90.0635**
6.25	2.6839	**42.9428**	849.4684	6.25	5.3092	**84.9468**
6.25	2.8467	**45.5470**	787.6040	6.25	4.9225	**78.7604**
14.00	0.0000	**0.0000**	188.6203	14.00	2.6407	**94.3102**
14.00	0.2969	**10.6053**	163.2996	14.00	2.2862	**81.6498**
14.00	0.0000	**0.0000**	167.1775	14.00	2.3405	**83.5888**
14.00	0.5934	**21.1931**	195.2369	14.00	2.7333	**97.6185**
6.25	3.6142	**57.8274**	1037.7346	6.25	6.4858	**103.7735**
6.25	2.4814	**39.7029**	1031.3615	6.25	6.4460	**103.1362**
6.25	2.4914	**39.8632**	733.8603	6.25	4.5866	**73.3860**
14.00	0.3452	**12.3299**	187.3642	14.00	2.6231	**93.6821**
14.00	0.4895	**17.4819**	178.3690	14.00	2.4972	**89.1845**

14.00	1.8430	**65.8230**	155.2277	14.00	2.1732	**77.6139**
14.00	1.3599	**48.5663**	356.9784	14.00	4.9977	**178.4892**
14.00	0.0000	**0.0000**	276.4479	14.00	3.8703	**138.2240**
6.25	2.6903	**43.0448**	843.7150	6.25	5.2732	**84.3715**
6.25	2.6883	**43.0121**	803.1279	6.25	5.0195	**80.3128**
6.25	2.5741	**41.1857**	762.7458	6.25	4.7672	**76.2746**
6.25	2.2539	**36.0621**	733.3505	6.25	4.5834	**73.3351**
6.25	2.2516	**36.0252**	642.3806	6.25	4.0149	**64.2381**
6.25	2.2468	**35.9482**	642.1145	6.25	4.0132	**64.2114**
6.25	2.7910	**44.6563**	810.1052	6.25	5.0632	**81.0105**
6.25	2.5516	**40.8259**	780.5926	6.25	4.8787	**78.0593**
6.25	2.5544	**40.8708**	750.6577	6.25	4.6916	**75.0658**
14.00	0.0000	**0.0000**	179.7252	14.00	2.5162	**89.8626**
14.00	0.6508	**23.2433**	184.8456	14.00	2.5878	**92.4228**
14.00	0.4973	**17.7617**	184.9928	14.00	2.5899	**92.4964**
6.25	3.7246	**59.5928**	1055.0870	6.25	6.5943	**105.5087**
6.25	3.8304	**61.2871**	1070.0601	6.25	6.6879	**107.0060**
6.25	3.9342	**62.9467**	1071.0242	6.25	6.6939	**107.1024**
14.00	0.9211	**32.8978**	246.3970	14.00	3.4496	**123.1985**
14.00	1.0857	**38.7763**	220.8999	14.00	3.0926	**110.4500**
14.00	0.4473	**15.9762**	141.0634	14.00	1.9749	**70.5317**
14.00	0.0000	**0.0000**	168.3110	14.00	2.3564	**84.1555**
6.25	3.6956	**59.1291**	991.2818	6.25	6.1955	**99.1282**
6.25	2.5346	**40.5543**	637.6287	6.25	3.9852	**63.7629**
6.25	2.7816	**44.5054**	872.8743	6.25	5.4555	**87.2874**
6.25	0.0000	**0.0000**	0.0000	6.25	0.0000	**0.0000**
6.25	2.7657	**44.2511**	809.1311	6.25	5.0571	**80.9131**
6.25	2.5185	**40.2965**	702.1750	6.25	4.3886	**70.2175**
6.25	3.1985	**51.1768**	872.4169	6.25	5.4526	**87.2417**

6.25	2.9343	**46.9485**	741.5087	6.25	4.6344	**74.1509**
6.25	3.2755	**52.4080**	913.4846	6.25	5.7093	**91.3485**
6.25	2.9970	**47.9515**	736.4145	6.25	4.6026	**73.6415**
6.25	2.9970	**47.9515**	604.4445	6.25	3.7778	**60.4445**
14.00	1.2943	**46.2257**	288.3094	14.00	4.0363	**144.1547**
14.00	0.6128	**21.8863**	183.6689	14.00	2.5714	**91.8345**
14.00	1.0650	**38.0353**	287.6341	14.00	4.0269	**143.8171**
6.25	3.2701	**52.3217**	1026.2394	6.25	6.4140	**102.6239**
6.25	3.3540	**53.6646**	974.2345	6.25	6.0890	**97.4235**
6.25	3.9342	**62.9467**	1109.9128	6.25	6.9370	**110.9913**
6.25	3.8175	**61.0794**	1094.2813	6.25	6.8393	**109.4281**
14.00	1.8117	**64.7034**	327.7777	14.00	4.5889	**163.8888**
14.00	0.8111	28.9676	259.5424	14.00	3.6336	**129.7712**
14.00	0.0000	0.0000	148.3501	14.00	2.0769	**74.1751**
